# MYC dephosphorylation by the PP1/PNUTS phosphatase complex regulates chromatin binding and protein stability

**DOI:** 10.1038/s41467-018-05660-0

**Published:** 2018-08-29

**Authors:** Dharmendra Dingar, William B. Tu, Diana Resetca, Corey Lourenco, Aaliya Tamachi, Jason De Melo, Kathleen E. Houlahan, Manpreet Kalkat, Pak-Kei Chan, Paul C. Boutros, Brian Raught, Linda Z. Penn

**Affiliations:** 1 Princess Margaret Cancer Centre, University Health Network, Toronto, M5G 1L7 ON Canada; 2 Department of Medical Biophysics, University of Toronto, Toronto, M5G 1L7 Canada; 3 Ontario Institute for Cancer Research, Toronto, ON Canada M5G 0A3 Canada; 4 Department of Pharmacology and Toxicology, University of Toronto, Toronto, Canada M5S 1A8 Canada

## Abstract

The c-MYC (MYC) oncoprotein is deregulated in over 50% of cancers, yet regulatory mechanisms controlling MYC remain unclear. To this end, we interrogated the MYC interactome using BioID mass spectrometry (MS) and identified PP1 (protein phosphatase 1) and its regulatory subunit PNUTS (protein phosphatase-1 nuclear-targeting subunit) as MYC interactors. We demonstrate that endogenous MYC and PNUTS interact across multiple cell types and that they co-occupy MYC target gene promoters. Inhibiting PP1 by RNAi or pharmacological inhibition results in MYC hyperphosphorylation at multiple serine and threonine residues, leading to a decrease in MYC protein levels due to proteasomal degradation through the canonical SCF^FBXW7^ pathway. MYC hyperphosphorylation can be rescued specifically with exogenous PP1, but not other phosphatases. Hyperphosphorylated MYC retained interaction with its transcriptional partner MAX, but binding to chromatin is significantly compromised. Our work demonstrates that PP1/PNUTS stabilizes chromatin-bound MYC in proliferating cells.

## Abstract (web summary)

Deregulated MYC activity is oncogenic and is deregulated in a large fraction of human cancers. Here the authors find that protein phosphatase 1 and its regulatory subunit PNUTS controls MYC stability and its interaction with chromatin.

## Introduction

In non-transformed cells, MYC protein expression is highly regulated by both transcriptional and post-transcriptional mechanisms, but MYC expression is deregulated in the majority of cancers. Deregulation occurs by well-established mechanisms involving gross genetic abnormalities (e.g., gene amplification or translocations) or by less defined mechanisms that can involve activated signaling cascades constitutively deregulating MYC activity^1–4^. MYC is a potent oncogene, in part because it is a master regulator that integrates multiple signaling cascades to regulate a wide variety of biological activities, including cellular proliferation, apoptosis, metabolism, and differentiation^4–7^. MYC orchestrates these activities by modulating gene transcription in association with MAX. The MYC-MAX heterodimer binds to E-box and non-E-box containing regulatory regions of 10–15% of all mammalian genes to control their expression and in turn, various biological processes^8–10^. MYC is highly responsive to signaling cascades, in part because it is a short half-life protein (~30 min), that is primarily regulated by the well-characterized GSK3/SCF^FBXW7^ pathway. Mitogen regulated kinases phosphorylate MYC at serine 62 (Ser62). GSK3β then phosphorylates threonine 58 (Thr58), which triggers protein phosphatase 2A (PP2A)-mediated Ser62 dephosphorylation. This ultimately leads to SCF^FBXW7^ E3 ligase-mediated MYC ubiquitylation and subsequent proteasomal degradation^1,11^.

Evidence from mouse models show that targeting MYC preferentially triggers tumor cells to undergo differentiation and/or apoptosis, leading to tumor regression^6,12,13^. Developing MYC inhibitors would significantly benefit cancer patient care and outcome, yet targeting MYC directly using traditional approaches has not been successful^14,15^. More recently, inhibitors such as JQ1, targeting a bromodomain protein (BRD4), were shown to down-regulate expression of several genes important for tumor maintenance, including MYC^16,17^. Indeed, clinical grade BRD4 inhibitors have been fast-tracked to phase I clinical trials in a wide variety of malignancies in which MYC plays a role^18^. This paradigm of targeting essential MYC regulators is promising and suggests that building an arsenal of MYC inhibitors at multiple levels of regulation will increase efficacy through use in combination therapy.

To this end, our goal was to better understand the post-translational modifications (PTMs) and regulators of MYC by interrogating the MYC interactome using BioID. We describe here the interaction of MYC with the PP1/PNUTS holoenzyme protein complex. MYC can induce PNUTS expression, suggesting a feed-forward co-operative regulatory loop. This is further supported by the co-localization of MYC and PNUTS to the promoters of MYC target genes. Inhibition of PP1/PNUTS triggers hyperphosphorylation of MYC, leading to chromatin eviction and degradation by the canonical SCF^FBXW7^ pathway. PP1/PNUTS is amplified  in multiple cancer types, suggesting a model in which elevated PP1/PNUTS expression confers a growth advantage by increasing MYC protein stability.

## Results

### MYC BioID identifies the PP1/PNUTS heterodimeric complex

**Fig. 1:**
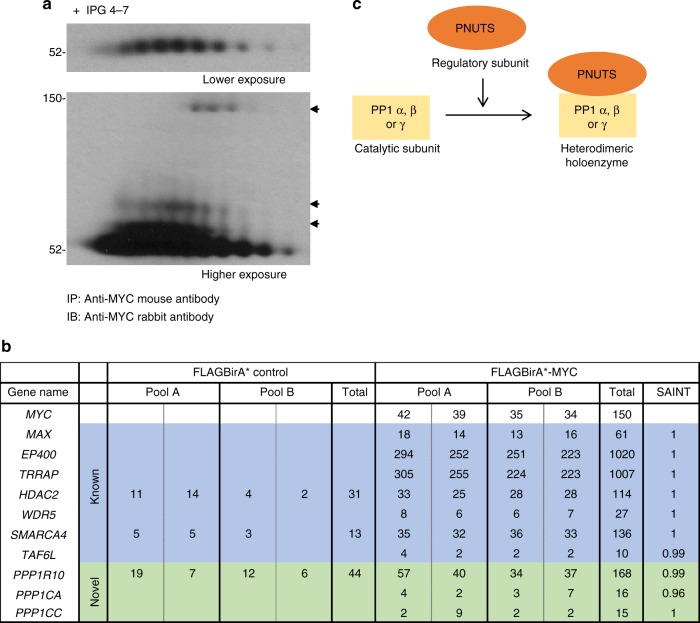
MYC is post-translationally modified and interacts with the PP1/PNUTS phosphatase complex. **a** Cell lysate from growing MCF10A cells was immunoprecipitated with MYC monoclonal mouse antibody, resolved by 2D gel electrophoresis (7 cm, pH 4–7 IPG strip; 10% SDS-PAGE), transferred onto nitrocellulose membrane and immunoblotted with MYC polyclonal rabbit antibody. Representative image of *n* = 3. Shown is lower exposure (top panel) and higher exposure (bottom panel) x-ray film images. Arrows show numerous high molecular weight MYC spots. **b** Shown are the gene names and spectral counts of selected known and novel MYC interacting proteins identified by conducting MYC-BioID MS in HeLa cells. A and B are biological replicates with two technical replicates shown for each. Total spectral counts and SAINT analysis score are presented. Significance is achieved with SAINT > 0.8. **c** Schematics of protein phosphatase 1 (PP1) and protein phosphatase 1 nuclear-targeting subunit (PNUTS). The catalytic subunit of PP1 α, β, or γ interacts with a regulatory subunit such as PNUTS, which forms a heterodimeric holoenzyme that interacts with PP1:PNUTS substrates

To investigate the regulation of MYC beyond the level of transcription, we evaluated PTMs and protein interactors of MYC. To assess MYC PTMs in a MYC-dependent transformation system, we used MCF10A cells. This is a genomically stable, non-transformed breast epithelial cell line that becomes transformed in response to ectopic deregulated MYC expression^19^. To determine whether MYC was post-translationally modified, we established a 2D electrophoresis assay in which MYC was immunoprecipitated from MCF10A cells, then separated by 2D electrophoresis, and immunoblotted. Interestingly, MYC migrated as several distinct “spots” suggesting that MYC harbors numerous PTMs in these growing cells (Fig. 1a). These several distinct MYC spots could be the result of many PTMs, including phosphorylation, acetylation, methylation, and/or glycosylation.

To identify MYC regulators, we used a proximity-dependent biotinylation technique, BioID^20,21^. Many of the technical problems associated with identifying MYC interactors using traditional approaches^22^ have been overcome with our recent development of MYC-BioID^20^. The N-terminus of full length MYC was fused in-frame with a BirA R118G (BirA*), an *E. coli* mutant protein biotin ligase. Ectopic expression of FLAGBirA*-MYC in cells supplemented with exogenous biotin allows proteins that are in close proximity to FLAGBirA*-MYC to be biotinylated. These biotinylated proteins are then enriched by affinity purification using streptavidin and identified by mass spectrometry (MS). This technique significantly increases the sensitivity and specificity of the detection of MYC interactors compared to previous MS based methods^20,22^. We generated HeLa cells to express FLAGBirA* control or FLAGBirA*-MYC and performed MYC-BioID as described^20^. Consistent with our previously identified MYC protein interactome conducted in HEK293 cells grown in tissue culture or as a tumor xenograft^20^, we identified many known (e.g., MAX, TRRAP, WDR5)^23–25^ and novel protein interactors using BioID (Fig. 1b; Supplementary Table 1).

Given that MYC is highly post-translationally modified (Fig. 1a), we prioritized our analysis on novel enzymatic interactors that were identified using MYC-BioID (Fig. 1b). To better understand phosphorylation-mediated regulation of MYC^1,19,26–30^, we focused on several novel MYC interactors that together form the PP1/PNUTS phosphatase complex, including the protein phosphatase 1 catalytic subunit alpha (PP1α; gene name: *PPP1CA*), protein phosphatase 1 catalytic subunit gamma (PP1γ; gene name:* PPP1CC*), and protein phosphatase-1 nuclear-targeting subunit (PNUTS; gene name:* PPP1R10*) (Fig. 1c). PP1 is a member of the PPP family of Ser/Thr phosphatases. Their substrate specificity, activity, and subcellular localization are controlled by interactions with regulatory subunits. The PP1 catalytic subunit has three isoforms (α, β, and γ), which interacts with a number of regulatory subunits and works as a heterodimeric holoenzyme^31^. PNUTS is a well characterized regulatory subunit of PP1 and targets PP1 to its substrates^32,33^. Using MYC-BioID, we identified the PP1/PNUTS enzyme complex as a potential novel regulator of MYC.

### MYC and PP1/PNUTS are amplified in cancer

**Fig. 2:**
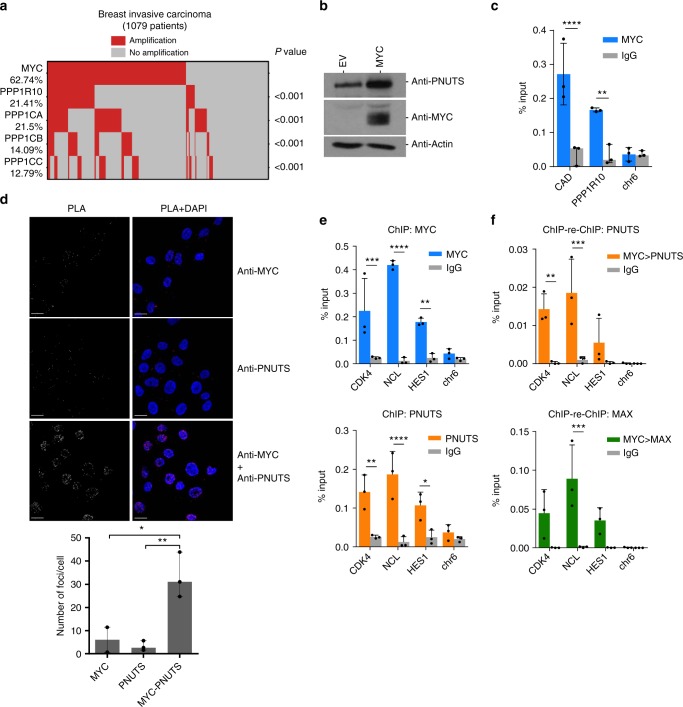
MYC and PP1/PNUTS are co-amplified in cancer and co-bind to promoters of MYC target genes. **a** *MYC* and *PPP1R10*, *PPP1CA*, *PPP1CB*, or *PPP1CC* gene amplification is evident and demonstrates significant co-occurrence in breast invasive carcinoma. **b** HO15.19 *MYC* knockout cells (EV) or cells ectopically expressing MYC were lysed and immunoblotted with antibodies to PNUTS, MYC, and actin. Shown are representative images of *n* = 3. **c** MCF10A cells were analyzed by chromatin immunoprecipitation (ChIP) using MYC or IgG antibody. ChIP’d DNA was quantified by qPCR for MYC or IgG binding to *CAD* and *PPP1R10* promoters or chr6 (negative control). **d** MCF10A cells probed with MYC rabbit antibody and/or a PNUTS mouse antibody. The proximity of MYC and PNUTS was assayed using the Duolink Proximity Ligation Assay (PLA) In Situ Red Starter kit (Sigma) as per manufacturer’s instructions. Representative PLA signal (red or white) and nuclear staining (DAPI; blue) are shown for cells probed with anti-MYC alone (top row), anti-PNUTS alone (middle row), or anti-MYC and anti-PNUTS (bottom row). PLA signal was quantified and shown as median number of foci per cell with range (*n* = 3). *p < 0.05, **p < 0.01, one-way ANOVA with Bonferroni test. Scale bars represent 20 µm. **e** MCF10A cells analyzed by ChIP with MYC, PNUTS or IgG antibody. ChIP’d DNA was quantified using qPCR for MYC, PNUTS or IgG binding to MYC target genes promoters, *CDK4, NCL*, and *HES1*, or chr6 (negative control). **f** For ChIP-re-ChIP analysis, MCF10A cells lysate incubated with MYC rabbit antibody followed by PNUTS (top panel), or MAX (bottom panel) antibody along with IgG antibody as a control. ChIP’d DNA was quantified similarly as above for ChIP experiment. For **c**, **e**, **f**: shown are % input mean (*n* = 3). *p < 0.05, **p < 0.01, ***p < 0.001, ****p < 0.0001, two-way ANOVA with Bonferroni test. Error bars represent s.d.

To determine whether MYC and its putative interactor, PP1/PNUTS, are both expressed in cancer, we examine the amplification status of the *MYC* gene, as well as genes of the PP1/PNUTS holoenzyme (*PPP1CA*, *PPP1CB*, *PPP1CC*, and *PPP1R10*) in The Cancer Genome Atlas (TCGA) datasets. Significant co-amplification for *MYC* and genes encoding the PP1/PNUTS complex was evident in patient tumor samples of several solid cancer types that severely impact human health, including breast (Fig. 2a), lung (adeno and squamous), and uterine carcinomas (Supplementary Fig. 1a-c). This suggests that the PP1/PNUTS phosphatase complex may contribute to the regulation of MYC in cancer, providing relevance for further investigation.

MYC has been previously shown to induce expression of its regulators, as part of a feed-forward regulatory loop^34^. To determine whether MYC can induce PP1/PNUTS expression, we evaluated whether MYC is bound to these gene regulatory regions. First, we analyzed ENCODE MYC and MAX ChIP-seq datasets, which revealed that MYC and MAX are both bound to the *PPP1R10, PPP1CA, PPP1CB*, and *PPP1CC* gene promoters (Supplementary Fig. 2). We also evaluated the expression of PNUTS in HO15.19 *MYC* knockout cells, with and without ectopic MYC expression and found that PNUTS protein expression is increased in response to ectopic MYC expression (Fig. 2b). Additionally, our own MYC ChIP analysis validated that MYC is bound to the *PPP1R10* promoter, as well as a known MYC-induced gene (*CAD*), but not to a negative control region (chr6) (Fig. 2c). Taken together, our data suggest MYC controls the expression of its interactors PP1/PNUTS through a feed-forward regulatory loop.

### MYC:PNUTS interaction is supported by PLA and ChIP-qPCR

The regulatory subunit (PNUTS) is the substrate specifying factor for the catalytic PP1 subunit. Therefore, we validated the MYC-PNUTS interaction by proximity ligation assay (PLA) in MCF10A cells. Addition of the rabbit MYC- or mouse PNUTS-specific antibodies alone yielded only background levels of fluorescence in cells (Fig. 2d); however, a specific nuclear fluorescence signal was detected when cells were probed with both MYC and PNUTS antibodies (Fig. 2d images (above) and histogram (below)). To further evaluate MYC-PNUTS interaction beyond the MCF10A cell line, we also evaluated A549 (lung adenocarcinoma), Daudi (lymphoma) (Supplementary Fig 3a images (left) and histogram (right)), OCI-AML3 (AML3) (leukemia), and H520 (squamous cell lung cancer) (Supplementary Fig. 3b images (left) and histogram (right)) cells and observed MYC-PNUTS interaction. To validate the specificity of the MYC PLA signal, we also performed PLA in HO15.19 *MYC* knockout cells. The MYC-null cells did not produce any fluorescence signal, but when MYC expression was restored there was an increase in signal with the MYC-PNUTS antibody pair (Supplementary Fig. 4a), demonstrating that the PLA signal was specific to MYC. Additionally, we also tested the specificity of PNUTS antibody in HEK293 cells transiently transfected with PNUTS sgRNA. PNUTS antibody detected a single band at the expected molecular weight in empty vector transfected cells, but this band was not evident in PNUTS sgRNA cells (Supplementary Fig. 4b), indicating that the PNUTS antibody is specific to its target. Taken together these data suggest that endogenous MYC and PNUTS interact in intact cells (Fig. 2d), and this interaction can be detected in a variety of cell lines by PLA (Supplementary Fig. 3 and 4).

To test whether MYC and PNUTS bind to MYC target gene promoters, MYC or PNUTS was subjected to ChIP-qPCR with target specific antibodies in MCF10A cells. MYC (Fig. 2e, top) or PNUTS (Fig. 2e, bottom) both displayed significant enrichment at promoters of known MYC target genes: *CDK4* (Cyclin Dependent Kinase 4), *NCL* (Nucleolin), and *HES1* (Hes Family BHLH Transcription Factor 1) compared to IgG control. A chromosome 6 (chr6) non-E box containing region was used as negative control. To evaluate the MYC and PNUTS co-binding to MYC target gene promoters, we performed MYC ChIP-re-ChIP with PNUTS (Fig. 2f, top) or MAX as a positive control (Fig. 2f, bottom) antibody. Both ChIP-re-ChIP analyses showed enrichment of MYC-MAX and MYC-PNUTS on MYC target gene promoters. These data indicate that MYC and PNUTS are bound to promoters of the same set of MYC target genes.

### MYC hyperphosphorylation and degradation

**Fig. 3:**
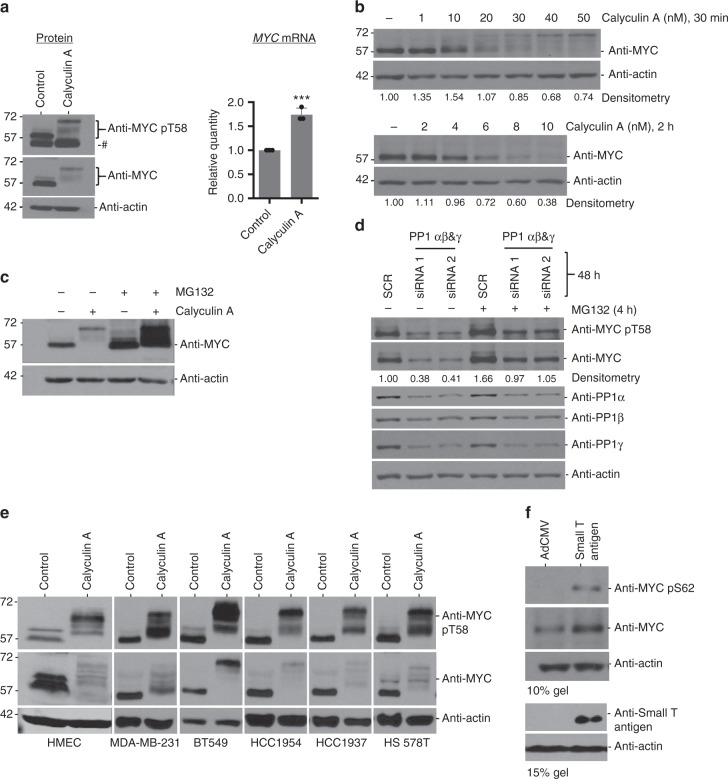
Inhibition of PP1 results in decreased MYC protein levels. **a** MCF10A cells treated with Calyculin A (50 nM) for 30 min and lysed for protein or RNA extraction. Immunoblots of lysates were probed with MYC mouse, MYC pT58 rabbit, or actin rabbit antibody (left panel). # indicates non-specific band. cDNA was made from extracted RNA and qRT-PCR was performed with primers against MYC or RPLP0 as an internal control. Data are from three biological replicates, mean is shown. ***p < 0.001; unpaired *t*-test, error bars represent s.d. **b** MCF10A cells were treated with Calyculin A at 1, 10, 20, 30, 40, 50 nM for 30 min (top panel) or 2, 4, 6, 8, 10 nM for 2 h (bottom panel) and lysed. Immunoblots were probed with MYC mouse or actin rabbit antibody. **c** Growing MCF10A cells were treated with MG132 (10 µM) for 4 h ± Calyculin A (50 nM) for 30 min and lysed. Immunoblots were probed with MYC mouse or actin rabbit antibody. **d** Growing MCF10A cells transfected with 10 nM siRNA directed against PP1α, β, and γ or universal scramble negative control by calcium phosphate. After 44 h transfection, cells were treated with 10 µM MG132 or vehicle control for 4 h and lysed. Immunoblots were probed with shown antibodies. **e** Growing HMEC, MDA-MB-231, BT549, HCC1954, HCC1937, or HS578T cells were treated with Calyculin A (50 nM) for 30 min and lysed. Immunoblots of lysates were probed with shown antibodies. **f** MCF10A cells expressing ectopic MYC were seeded, grown overnight, serum starved for 24 h, and then infected with 200 MOI of empty vector adenovirus (AdCMV) or adenovirus carrying small T antigen. Cells were grown for 22 h in 0.25% serum media, lysed, and lysates immunoblotted with indicated antibodies. Shown are representative images of *n* = 3

To explore the functional relevance of the MYC interaction with PP1/PNUTS, we inhibited PP1 using Calyculin A, a pharmacological PP1 inhibitor. MCF10A cells exposed to Calyculin A for 30 min were harvested and analyzed by immunoblot using an antibody to MYC-pT58 or MYC (Fig. 3a, left). In response to PP1 inhibition, multiple phospho-MYC species (pMYC), as indicated using anti-MYC pT58, migrated at higher molecular weight (Fig. 3a, top blot) and the abundance of total MYC was reduced (Fig. 3a, middle blot). The decreased level of MYC protein was not due to reduction in total *MYC* mRNA (Fig. 3a, right), as *MYC* mRNA levels, by contrast, increased after Calyculin A treatment. This may be due to a homeostatic feedback response, in an attempt to rescue the decreased MYC protein levels. The reduction in total MYC protein abundance in response to Calyculin A was both dose-dependent and time-dependent (Fig. 3b). The hyperphosphorylated MYC species were captured and visualized only in response to a relatively acute exposure (30 min) (Fig. 3b, top) but not to a longer exposure (2 h) (Fig. 3b, bottom) to Calyculin A. This suggests that MYC hyperphosphorylation occurs prior to degradation and does not accumulate in sufficient abundance for detection at the lower doses and longer time points of Calyculin A treatment due to resultant degradation of MYC (Fig. 3b). The cells did not appear to be undergoing apoptosis in response to Calyculin A as we did not observe PARP cleavage after a 30-min treatment (Supplementary Fig. 5a). MYC degradation in response to Calyculin A was blocked by treatment with the proteasomal inhibitor MG132, enabling the higher migrating hyperphosphorylated species of MYC to be readily visualized (Fig. 3c). To test the specificity of PP1 inhibition on MYC levels, two different siRNA cocktails targeting all PP1 catalytic subunits (PP1 α, β, and γ) were evaluated. Consistently, PP1 knockdown resulted in lower levels of MYC protein compared to control. MYC protein levels, but not PP1, could be partially rescued with 4-h MG132 treatment (Fig. 3d). PP1 knockdown did not significantly change *MYC* mRNA levels (Supplementary Fig. 5b).

To evaluate these results beyond MCF10A cells, we assayed the response to PP1 inhibition across a panel of cell lines. These include primary non-transformed, hTERT-immortalized human mammary epithelial cells (HMEC), breast cancer (MDA-MB-231, BT549, HCC1954, HCC1937, HS578T) (Fig. 3e), leukemia (OCI-AML2, OCI-AML3, referred to as AML2 and AML3 respectively), lymphoma (Raji, Daudi), lung adenocarcinoma (A549, HCC827), squamous cell lung cancer (H520, H2170), and uterine corpus endometrial carcinoma (HEC-1-A) cell lines (Supplementary Fig. 5c). These different cells lines universally displayed a similar response to Calyculin A treatment, which demonstrates that PP1/PNUTS regulation of MYC phosphorylation and degradation occurs across multiple cell types. As the Raji B-cell lymphoma cells carry a mutation of MYC T58^35^, the pT58 signal was not detectable in these cells (Supplementary Fig. 5c). This data reinforces the specificity of MYC phospho antibody to pT58 and importantly also demonstrates the involvement of phospho residues in addition to T58 in the Calyculin A-induced MYC molecular weight shift.

Calyculin A inhibits both PP1 and PP2A^36^, therefore it was important to determine whether inhibition of PP2A showed the same effect on MYC stability as Calyculin A. To this end, we transduced MCF10A cells with recombinant adenovirus that expressed SV40 small T antigen, a specific inhibitor of PP2A^37^. Small T antigen increased Ser62 phosphorylation as expected and increased MYC levels (Fig. 3f), but did not result in MYC degradation as was seen with Calyculin A-dependent PP1 inhibition (Fig. 3a). Additionally, we also treated cells with Okadaic acid, a PP2A inhibitor, which increased Ser62 phosphorylation (Supplementary Fig. 5d), but did not result in MYC degradation as was seen with PP1 inhibition. Taken together, these data suggest that an increase in MYC phosphorylation at multiple sites triggers MYC degradation, and PP1 is required for MYC stability.

### PP1 dephosphorylates MYC

**Fig. 4:**
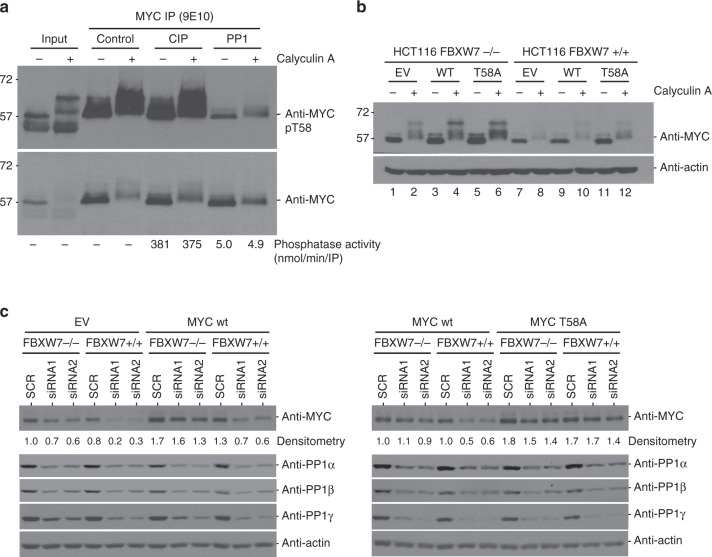
PP1 dephosphorylates MYC and rescues it from SCF^FBXW7^-dependent degradation. **a** MCF10A cells were treated with Calyculin A (50 nM) or DMSO control for 30 min, lysed, and immunoprecipitated with MYC mouse antibody. The IP was treated with an enzyme control, calf intestinal alkaline phosphatase (CIP) or protein phosphatase 1 (PP1) for 2 h at 30 °C. Total CIP or PP1 enzyme activity in the IP reaction after the 2-h incubation was measured using the substrate p-Nitrophenyl phosphate (PNP). The IP was loaded on a 10% SDS-PAGE, transferred onto nitrocellulose membrane and immunoblotted with MYC pT58 rabbit or MYC rabbit antibody. Shown are representative images of *n* = 3. **b** HCT116 FBXW7 knockout (−/−) or wild-type (+/+) cells expressing inducible ectopic empty vector (EV), wildtype MYC (wt) or MYC T58A (T58A) following 4-hydroxytamoxifen (4-OHT) (48 h) were treated with vehicle control (−) or  Calyculin A (50 nM) (+) for 30 min or **c** transfected with 10 nM siRNA directed against PP1α, β, and γ or universal scrambled negative control by calcium phosphate for 48 h and lysed. Protein lysates (20 µg) were loaded onto 10% SDS-PAGE, transferred onto nitrocellulose membrane and immunoblotted with indicated antibodies. Normalized MYC densitometry values are shown below the MYC blot. Shown are representative images of *n* = 3

We next evaluated whether exogenous PP1 or another phosphatase could remove Calyculin A-induced hyperphosphorylation of MYC. Briefly, MYC was immunoprecipitated from lysates of growing MCF10A cells treated with or without Calyculin A. The immunoprecipitated material was treated with exogeneous PP1 or calf intestinal alkaline phosphatase (CIP), and then immunoblotted with MYC pT58 or MYC antibody (Fig. 4a). The results demonstrated that introduction of PP1, but not CIP, resulted in dephosphorylation of both basal and PP1 inhibitor-induced MYC phosphorylation in control and Calyculin A-treated cells, respectively, showing that exogenous PP1 could reverse the hyperphosphorylation of MYC in response to Calyculin A. The lack of CIP effect was not due to loss of its enzyme activity. We tested CIP and PP1 phosphatase activity at the end of the 2-h incubation period with p-Nitrophenyl phosphate (PNP) as a substrate (Fig. 4a bottom). Indeed, CIP activity was several fold higher than PP1 but still CIP could not dephosphorylate MYC. This suggests that the slower migrating bands were indeed the result of accumulated phosphorylation of MYC and that PP1 can dephosphorylate MYC.

MYC degradation is primarily regulated by the SCF^FBXW7^ E3 ligase^1,11^; however several additional E3 ligases have recently been shown to regulate MYC activity^1^. To determine the role of the canonical SCF^FBXW7^ pathway, we studied the effect of PP1 inhibition by Calyculin A in HCT116 *FBXW7* knockout or wild-type cells; knockout cells carry the *FBXW7* gene exon 5 deletion which encodes for an inactive enzyme^38^. These cells were virally transduced and selected to express empty vector, MYC wildtype or the MYC T58A mutant and treated with Calyculin A or vehicle control. Consistently, PP1 inhibition by Calyculin A treatment resulted in lower levels of MYC in *FBXW7* wild-type cells compared to *FBXW7* knockout cells (Fig. 4b; compare lanes 7–12 to 1–6). The *FBXW7* knockout status in HCT116 cells was validated by PCR (Supplementary Fig. 5e). MYC T58A levels were also decreased but not to the same extent as MYC wildtype, suggesting additional E3 ligases can contribute to Calyculin A induced degradation of MYC (Fig. 4b; compare lanes 10 and 12). Similarly, the important functional role of FBXW7 to MYC degradation following PP1 inhibition was also evident in these cells following siRNA knockdown of PP1 (Fig. 4c). This demonstrates that MYC degradation in response to PP1 inhibition is primarily due to the canonical SCF^FBXW7^ pathway with additional, minor contributions from other unknown mechanisms.

### PP1 regulates several MYC serine/threonine residues

**Fig. 5:**
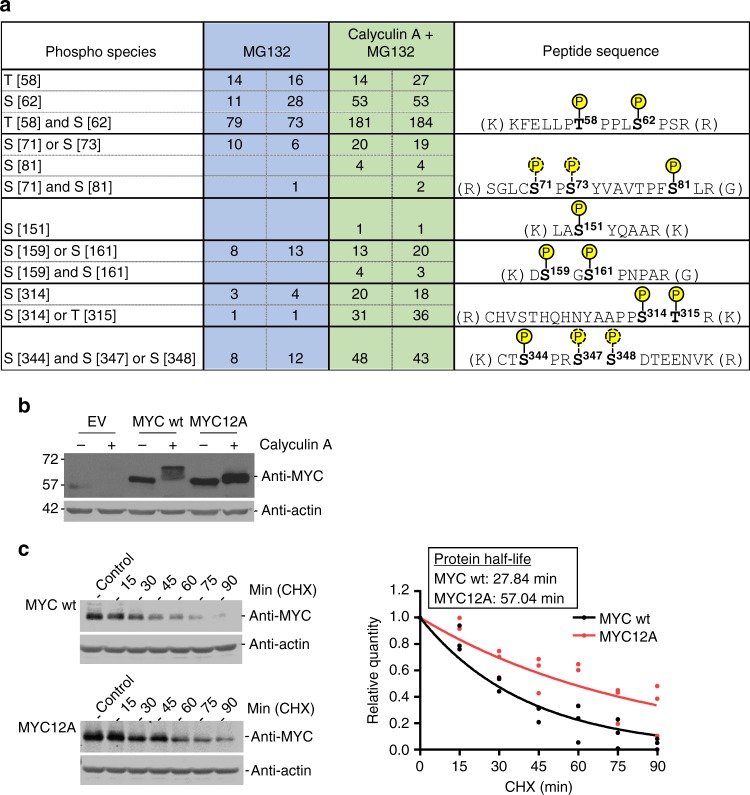
PP1 dephosphorylates multiple serine and threonine residues on MYC. **a** Growing MCF10A cells were treated with MG132 (10 µM) for 4 h ± Calyculin A (50 nM) for 30 min, lysed, and MYC immunoprecipitated and then subjected to trypsin digestion, phospho-enrichment and analysis by MS. Shown are the peptide counts from two technical replicates as well as the peptide with the indicated phospho-sites. **b** MCF10A cells treated with 4-OHT (48 h) to induce expression of empty vector (EV) wildtype MYC (MYC wt) or a MYC mutant in which the 12 Ser and Thr residues identified in **a** were mutated to Ala (MYC12A), were treated with Calyculin A (50 nM) for 30 min and lysed. Protein lysate (20 µg) was loaded on 10% SDS-PAGE, transferred onto nitrocellulose membrane and immunoblotted with MYC mouse or actin rabbit antibody. Shown are representative images of *n* = 3. **c** MCF10A cells expressing MYC wt or MYC12A after 4-OHT (24 h) induction were treated with cycloheximide (CHX) (10 µg/mL), harvested at 15-min intervals, lysed, resolved by SDS-PAGE and immunoblots probed with MYC or actin antibody (left). The MYC band intensities were quantified and signal normalized to actin. Shown are the normalized MYC signals for three replicates with protein half-life determinations (right)

We next determined which of the many potential phosphorylation sites of MYC were dephosphorylated by PP1. MCF10A cells were exposed to MG132 to block MYC degradation and then treated with vehicle control or Calyculin A (Fig. 3c). Equal amounts of MYC were immunoprecipitated, subjected to tryptic digestion followed by immobilized metal affinity chromatography (IMAC)-based phosphopeptide enrichment, and phosphopeptides were identified by MS. Spectral counts for specific MYC phosphopeptides were increased in response to Calyculin A treatment (Fig. 5a). Increased phosphorylation levels were observed at previously characterized (Thr58, Ser62, Ser71, Ser81, Ser344, Ser347, and Ser348) and novel phospho-residues (Ser151, Ser159, Ser161, Ser314, and Thr315). Thus, the phosphorylation of numerous residues was regulated by PP1, suggesting their potential role in regulating MYC degradation.

To evaluate the role of the PP1 modified residues identified by MS, we performed site directed mutagenesis of the Ser/Thr residues phosphorylated in response to PP1 inhibition. Interestingly, mutating these twelve Ser/Thr residues (Thr58, Ser62, Ser71, Ser81, Ser151, Ser159, Ser161, Ser314, Thr315, Ser344, Ser347, and Ser348) to Ala (MYC12A) inhibited the majority of the Calyculin A triggered shift and degradation (Fig. 5b). The lack of complete rescue suggests that additional residues not detected by MS may be modified in response to PP1 inhibition.

To evaluate the effect of these 12 phosphorylated residues on MYC stability, we evaluated the MYC wildtype and MYC12A protein half-life by treating cells with cycloheximide. Consistently, mutating these twelve Ser/Thr MYC residues resulted in an increased protein half-life from 27.84 to 57.04 min (Fig. 5c). Thus, after PP1 inhibition, MYC is phosphorylated on several Ser/Thr residues. Mutation of 12 of these residues to Ala precluded the majority of MYC hyperphosphorylation and degradation that were evident following PP1 inhibition, and increased MYC half-life.

### MYC hyperphosphorylation reduces chromatin binding

**Fig. 6:**
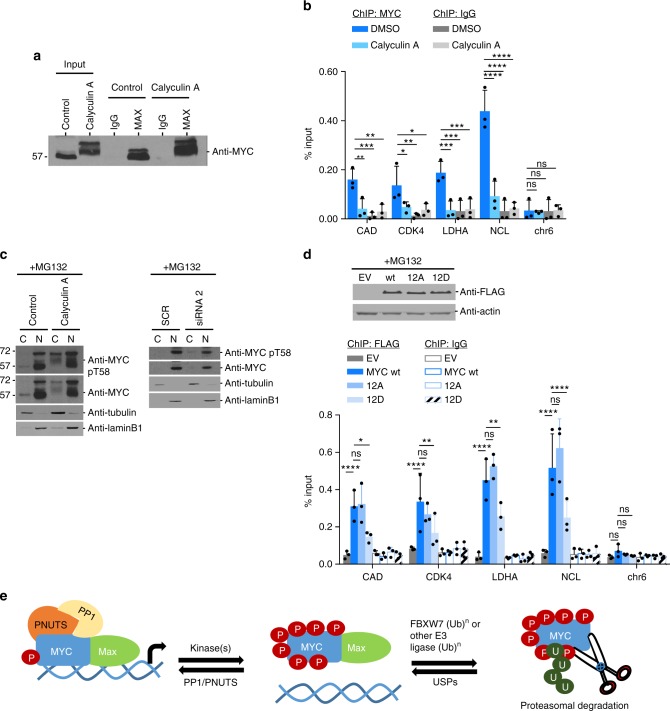
Hyperphosphorylated MYC retains interaction with MAX but is compromised for chromatin binding. **a** MCF10A cells were treated with Calyculin A (50 nM) or vehicle control for 30 min, lysed, and immunoprecipitated with MAX rabbit or IgG rabbit antibody. The input and IP was resolved by SDS-PAGE and immunoblotted with MYC mouse antibody. Shown is a representative image of *n* = 3. **b** For ChIP analysis, MCF10A cells were treated with MG132 (10 µM) for 4 h ± Calyculin A (50 nM) for 30 min, and lysate incubated with MYC rabbit antibody along with IgG antibody as a control. ChIP’d DNA was quantified by qPCR for MYC target genes promoters of *CAD, CDK4, LDHA*, and *NCL*, and chr6 (negative control). **c** For nuclear and cytoplasmic fractionation, MCF10A cells were treated with MG132 (10 µM) for 4 h ± Calyculin A (50 nM) for 30 min (left panel), or transfected with 10 nM siRNA directed against PP1α, β, and γ or universal scramble negative control by calcium phosphate for 44 h and then treated with MG132 (10 µM) for 4 h (right panel). Lysates were used for fractionation, and immunoblotted with indicated antibodies. **d** MCF10A cells carrying inducible FLAG-tagged EV, MYC wt, MYC12A, or MYC12D were treated with 4-OHT (24 h) to induce expression, treated with MG132 (10 µM) for 4 h, and then lysed for immunoblot analysis (top) or for ChIP-qPCR with FLAG or IgG antibody (bottom) as outlined in **b** above. For **b**, **d**: shown are % input mean (MYC and FLAG ChIP: *n* = 3). *p < 0.05, **p < 0.01, ***p < 0.001, ****p < 0.0001, two-way ANOVA with Bonferroni test. Not statistically significant = ns. Error bars represent s.d. **e** Proposed model of PP1/PNUTS regulation of MYC hyperphosphorylation in the control of chromatin binding and degradation

As MYC interaction with MAX is required for MYC transcription^3,9^, we examined the ability of hyperphosphorylated MYC to interact with MAX. MAX was immunoprecipitated from MCF10A cells with or without Calyculin A exposure and immunoblotted for MYC (Fig. 6a). Hyperphosphorylated MYC and MAX interaction was intact, suggesting that MYC hyperphosphorylation does not interfere with MAX binding. To evaluate the ability of hyperphosphorylated MYC to bind chromatin, MCF10A cells were treated with MG132 and either vehicle control or Calyculin A, and then ChIP-qPCR was conducted using anti-MYC or IgG control to evaluate binding to MYC target gene promoters. MYC ChIP under hyperphosphorylated conditions showed significant reduction in MYC binding to MYC target genes, including *CAD, CDK4*, *NCL*, and *LDHA* gene promoters compared to MYC ChIP under control conditions (Fig. 6b). By contrast, PNUTS binding to these MYC target gene promoters was unaffected under similar conditions (Supplementary Fig. 6a). IgG control ChIP showed little difference between the two conditions on any of the MYC target genes. Additionally, to test chromatin integrity and control for any global effects of Calyculin A, we performed ChIP-qPCR analysis using a Histone H3 antibody and observed no significant differences in chromatin integrity (Supplementary Fig. 6b). To further evaluate the subcellular localization of hyperphosphorylated MYC, nuclear-cytoplasmic fractionation was performed in MCF10A cells treated with MG132 and either vehicle control or Calyculin A (Fig. 6c left), or transfected with PP1 siRNA (Fig. 6c right). Irrespective of Calyculin A treatment or PP1 knockdown, MYC pT58 or total MYC was predominantly detected in nuclear fractions.

To further evaluate the effect of MYC hyperphosphorylation on chromatin binding by an alternative approach, we mutated the 12 Ser/Thr MYC phospho-residues to either Ala (MYC12A) or the phosphomimetic Asp (MYC12D) and introduced inducible expression vectors carrying EV, N-terminally FLAG-tagged MYC or these MYC mutants into MCF10A cells. Cells induced with 4-OHT and MG132 treatment showed comparable FLAG-tagged MYC wildtype, MYC12A, or MYC12D expression (Fig. 6d top). However, ChIP with anti-FLAG or IgG control showed significant reduction in MYC12D binding to MYC target genes, including *CAD, CDK4*, *LDHA*, and *NCL* gene promoters, compared to MYC wildtype or MYC12A (Fig. 6d below). This suggests that the phosphomimetic MYC12D mutant mimics MYC phosphorylation on these 12 Ser/Thr residues and antagonizes chromatin binding. In summary, MYC hyperphosphorylation on twelve Ser/Thr residues has no effect on MAX binding, but significantly alters MYC binding to target gene promoters and demonstrates a novel post-translational mechanism to control MYC activity.

## Discussion

We conducted an unbiased MYC-BioID MS protein–protein interaction screen and identified the PP1/PNUTS holoenzyme as a MYC interactor. PLA was used to validate the endogenous MYC-PNUTS interaction and sequential ChIP-re-ChIP of MYC followed-by PNUTS revealed that both proteins bind to MYC-bound gene promoters. MYC can induce PP1/PNUTS expression, suggesting a feed-forward loop in which MYC drives PP1/PNUTS expression to enable their interaction and regulate gene transcription. Consistent with these results, recent genome-wide DamID analysis demonstrated that PP1 and PNUTS promoter-binding significantly overlaps with MYC ChIP-seq datasets from the ENCODE consortium^39^. This is consistent with our ChIP results and suggests that MYC and PP1/PNUTS promoter occupancy can co-occur genome-wide. RNAi and pharmacological inhibitors of PP1 trigger the hyperphosphorylation of MYC, resulting in chromatin dissociation and MYC protein degradation through the canonical SCF^FBXW7^ pathway. Importantly, exogenous introduction of PP1 can rescue MYC from this hyperphosphorylated state. PP1/PNUTS genes are co-amplified in several cancer types, suggesting that one role of this phosphatase is to dephosphorylate MYC to keep it in a stable, chromatin-bound configuration.

At first glance, it is somewhat surprising that MYC activity and stability are enhanced by PP1/PNUTS, considering MYC is one of the mitogen-stimulated, immediate early genes whose expression is routinely up-regulated in response to a wide-variety of pro-growth stimuli^2^. It was anticipated that these mitogen-activated kinase signaling cascades would lead to MYC phosphorylation, which would promote MYC activity. However, our data in conjunction with the literature paint a different picture in which phosphorylation can both promote and restrict MYC activity. The specific residues that are modified and the degree of phosphorylation appear to determine the net effect on MYC function. For example, phosphorylation of MYC Ser62 by MAPK increases MYC activity, but also primes Thr58 for phosphorylation, which promotes MYC ubiquitylation and degradation^1^. This phosphorylation is highly regulated as a recent study showed that Thr58 phosphorylation could be removed by the EYA1 phosphatase^40^. Mutating MYC Thr58 to Ala resulted in increased MYC stability and transformation^19^ and mutation of this site is evident in Burkitt Lymphoma^41^, thus locking MYC into a more stable and active form. Concomitant regulation of multiple phosphorylation sites can also impact MYC stability and/or activity. For example, we have previously shown that mutating Ser71 or Ser81 to Ala individually had no effect on MYC dependent transformation, but mutating both S71/S81 or another cluster of MYC phospho-sites, Thr343/Ser344/Ser347/Ser348, to Ala increased the ability of MYC to drive transformation^19^. MYC phosphorylation on Thr358, Ser373, and Thr400 by the stress-responsive kinase Pak2 reduces MYC activity by interfering with MAX dimerization or DNA binding^28^. MYC phosphorylation on Ser373 by PKCζ reduces MYC activity^42^ and phosphorylation on Thr244 results in reduced stability^29^. In the mitotic phase of the cell cycle, MYC hyperphosphorylation has been suggested to reduce MYC binding to chromatin during chromatin condensation^43^. Thus, MYC regulation by site-specific phosphorylation involves single or multiple, often clustered, residues and primarily leads to reduced activity and/or stability.

Based on previous studies and our results, we propose a simple model: when MYC is bound to chromatin and regulating gene transcription, it resides in a relatively less phosphorylated state. Upon hyperphosphorylation, MYC no longer binds chromatin and is then either dephosphorylated by PP1/PNUTS and recycled to regulate gene transcription or is degraded through the SCF^FBXW7^ or other E3 ligase mediated pathways (Fig. 6e). These multiple phosphorylation events may lead to a change in overall charge that may then alter MYC interaction with proteins and/or DNA. Thus, phosphorylation of MYC can negatively regulate MYC activity.

The detailed interaction of PP1/PNUTS with most of its substrates remains unclear. Interestingly, RNA polymerase II (RNAPII) has been established as an endogenous substrate for the PP1/PNUTS holoenzyme to regulate the dephosphorylation of the C-terminal domain at residue Ser5^33,44^. Given that MYC has been shown to play a role in both the initiation and elongation of gene transcription, it is tempting to speculate that PP1/PNUTS may regulate both MYC and RNAPII to control gene transcription. Further studies to better define the molecular basis of PP1/PNUTS with MYC in the context of transcription control will provide insight as to how PP1/PNUTS regulates MYC phosphorylation and function. This information will also guide the design of inhibitors targeting the PP1/PNUTS and MYC interaction to drive the inactivation and degradation of MYC.

## Methods

### Cell lines

MCF10A cells were cultured as previously described^45^, HO15.19 (kindly provided by Dr. John Sedivy), MDA-MB-231 (HTB-26), HS 578T (HTB-126), HCT116 (CCL-247), and HeLa (CCL-2) cells were cultured in DMEM supplemented with 10% FBS, HCC1937 (CRL-2336), BT549 (HTB-122), HCC1954 (CRL-2338), A549 (CRM-CCL-185), HCC827 (CRL-2868), Raji (CCL-86, kindly provided by Dr. Eleanor Fish), H520 (HTB-182, kindly provided by Dr. Christine Allen), H2170 (CRL-5928), and Daudi (CCL-213) cells were cultured in RPMI-1640 supplemented with 10% FBS, OCI-AML2 (ACC-99) and OCI-AML3 (ACC-582) cells were cultured in Alpha-MEM supplemented with 10% FBS, HEC-1-A cells (HTB-112) were cultured in McCoy’s 5A supplemented with 10% FBS, and HMEC (CRL-3243) cells were cultured in MEGM with BPE, hEGF, hydrocortisone, GA-1000, and insulin (Lonza CC-3150). HCT116 FBXW7−/− or +/+ cells were obtained from Dr. Lars-Gunner Larson with permission from Dr. Bert Vogelstein.

### BioID in cell culture

HeLa cells at ~70% confluency, carrying tetracycline-inducible FLAGBirA* or FLAGBirA*-MYC, were treated for 24 h with 1 μg/mL tetracycline, 5 μM MG132, and 50 μM biotin, to induce the expression of the transgene and facilitate biotin labelling. Cells were then scraped into phosphate buffered saline (PBS), washed two times with 25 mL of PBS, and centrifuged at 1000 × *g* for 5 min at 4 °C. Cell pellets were lysed in 10 mL of cold modified RIPA buffer (w:v; 1% NP-40, 50 mM Tris–HCl pH 7.5, 150 mM NaCl, 1 mM EDTA, 1 mM EGTA, 0.1% SDS, 1:500 protease inhibitor cocktail (Sigma) 0.5% sodium deoxycholate), supplemented with 250 U of benzonase (EMD). Lysate was end-over-end rotated for 1 h at 4 °C, sonicated 3 × 30 s (Fisher Scientific D100 Sonic Dismembrator), and centrifuged at 27,000 × *g* for 30 min at 4 °C. Biotinylated proteins were isolated by affinity purification with 30 µg of (RIPA-equilibrated) streptavidin-sepharose beads (GE) with end-over-end rotation for 2 h at 4 °C. Beads were washed 7 × 1 mL 50 mM ammonium bicarbonate (pH 8.0) prior to tryptic digest.

### Mass spectrometry for BioID

Tryptic digestion was performed with 1 μg of MS-grade TPCK trypsin (Promega, Madison, WI) dissolved in 100 μL of 50 mM ammonium bicarbonate (pH 8.0) which was added to the streptavidin-sepharose beads and incubated at 37 °C overnight. The eluate was collected and beads were washed twice in 150 µL of 50 mM ammonium bicarbonate. Eluate and washes were pooled, lyophilized and reconstituted in 0.1% formic acid. Liquid chromatography analysis was performed on an in-house analytical column (75 µm inner diameter) and pre-column (150 µm inner diameter), made from fused silica capillary tubing from InnovaQuartz (Phoenix, AZ), and packed with 100 Å C18-coated silica particles (Magic, Michrom Bioresources, Auburn, CA).

Peptides were resolved and identified using reversed phase (120 min buffer gradient 10–40% acetonitrile, 0.1% formic acid) nanoflow liquid chromatography-electrospray ionization-tandem mass spectrometry (nLC-ESI-MS/MS), running at 250 nL/min on a Proxeon EASY-nLC pump in-line with a hybrid linear quadrupole ion trap Orbitrap mass spectrometer, Velos LTQ (ThermoFisher Scientific, Waltham, MA). A parent ion scan was performed in the Orbitrap using resolving power of 60,000. Up to 20 most intense peaks (minimum ion count of 1000) were selected for MS/MS using standard CID fragmentation. Fragment ions were detected in the LTQ. Dynamic exclusion was activated, where MS/MS of the same *m/z* (within a 10 ppm window, exclusion list size 500) detected two times within 15 s were excluded from analysis for 30 s. For protein identification, Proteowizard^46^ was used to convert Thermo.RAW files to the.mzXML, and then searched using X!Tandem^47^ against Human RefSeq Version 45 (appended with cRAP and reversed decoy database based on RefSeq v45). Search parameters specified a parent MS tolerance of 15 ppm and an MS/MS fragment ion tolerance of 0.4 Da, with up to two missed cleavages allowed for trypsin. Oxidation of methionine and ubiquitylation of lysine residues were allowed as variable modifications. Data were analyzed using trans-proteomic pipeline^48^ via the ProHits software suite^49^. Proteins identified with ProteinProphet cut-off of 0.8 (corresponding to FDR < 1%) were analyzed with SAINT Express v. 3.3^50,51^.

### Mass spectrometry for PTMs

PTM analysis of MYC was performed in MCF10A cells. Briefly, 2 × 10^8^ MCF10A cells were grown to approximately 60% confluency and treated with 10 µM of MG132 for 4 h or with 10 µM MG132 for 4 h and 50 nM Calyculin A for 30 min. Cell were then scraped into PBS, pooled and washed once with 50 mL of PBS with centrifugation at 1000 × *g* for 5 min at 4 °C. Cell pellets were lysed in 1 mL of modified Cell Lysis Buffer (w:v; 1% SDS, 100 mM Tris–HCl pH 6.8, 1x protease inhibitor cocktail (Sigma)), supplemented with 250 U of benzonase (EMD), 250 µM sodium orthovanadate, 10 mM NaF and 50 mM beta-glycerolphosphate, pH7.5. Lysed cells were then collected into a 50 mL Falcon tube, boiled for 10 min at 95 °C and sonicated 3 × 30 s. Solution was reconstituted to 20 mL with PBS and centrifuged at 27,000 × g for 30 min at 4 °C. Cell lysates were then used for MYC IP using 60 µL commercially crosslinked 9E10 antibody beads (Sigma). Samples were incubated overnight at 4 °C. Beads were washed 7 × 1 mL of 50 mM ammonium bicarbonate (pH 8.0). MYC protein was eluted off the beads in two steps using 300 µL of MS Elution Buffer (0.5 M Ammonium hydroxide). Eluates were pooled and lyophilized. Samples were resuspended in 200 µL of 50 mM ammonium bicarbonate buffer for reduction and alkylation. DTT was added to samples to a final concentration of 5 mM for the reduction step, and samples were incubated for 30 min at 55 °C. After samples were cooled to room temperature, iodoacetomide solution was added to each sample to a final concentration of 15 mM. Samples were incubated in the dark for 1 h at room temperature. Tryptic digestion was performed with 1 µg of MS-grade TPCK trypsin, which was added to the samples followed by an incubation at 37 °C overnight. Samples were split into two fractions and lyophilized prior to phosphopeptide enrichment. Phosphopeptide enrichment was performed using PHOS-Select Iron Affinity Gel (Sigma) or Titansphere Phos-TiO (GL Sciences Inc.) kits using manufacturer protocols. Samples were reconstituted in 0.1% formic acid and subjected to nLC-ESI-MS/MS on a Proxeon EASY-nLC pump in-line with a hybrid linear quadrupole ion trap Orbitrap mass spectrometer, Velos LTQ, as outlined earlier. For protein identification, Proteowizard was used to convert Thermo.RAW files to the.mzXML, and then searched using X!Tandem on the GPM interface^52^. Search parameters specified a parent MS tolerance of 15 ppm and an MS/MS fragment ion tolerance of 0.4 Da, with up to two missed cleavages allowed for trypsin. Oxidation of methionine, ubiquitylation of lysine and carbomidomethylation of cystine residues, phosphorylation of serine and threonine were allowed as variable modifications.

### Proximity ligation assay (PLA)

Cell lines grown in suspension were centrifuged at 200 × *g* for 5 min at room temperature, washed in cold PBS with 2% FBS, centrifuged at 200 × g for 5 min at 4 °C, and finally resuspended in 100 µL PBS with 2% FBS. Cells were then centrifuged onto Superfrost® Plus Micro Slides (48311-703, VWR) using the Thermo Scientific™ Cytospin™ 4 Cytocentrifuge at 600 rpm for 10 min. For adherent cell lines, cells were grown on coverslips in 12-well plates. PLA was performed with antibodies against MYC (06–340, Millipore) and/or PNUTS (611060, BD Biosciences) as described previously^20^.

### Chromatin immunoprecipitation (ChIP) and ChIP-re-ChIP

*ChIP-qPCR*. ChIP was performed as described previously^53^ and as follows. ChIP-qPCR for MYC (homemade N262) and PNUTS (A300-440A, Bethyl Laboratories) was performed with 10 × 10^6^ cells and for histone H3 antibody (ab1791, Abcam) was performed with 5 × 10^6^ cells crosslinked in 1% formaldehyde per ChIP reaction. Following lysis in Cell Lysis Buffer (1% SDS, 10 mM EDTA, 50 mM Tris-HCl pH 8.1), samples were sonicated with Bioruptor Pico (Diagenode) and diluted in Dilution Buffer (1% Triton X-100, 2 mM EDTA, 150 mM NaCl, 20 mM Tris-HCl pH 8.1) with pre-incubated mixture of antibody and Dynabeads (Invitrogen). After overnight incubation, samples were washed in Washing Buffer (50 mM HEPES pH 7.6, 1 mM EDTA, 0.7% sodium deoxycholate, 1% NP-40, 0.5 M LiCl) then TE Buffer. DNA was eluted in Decrosslinking Buffer (1% SDS, 0.1 M NaHCO_3_) overnight, purified with QIAquick PCR Purification Kit (Qiagen), and analyzed by qPCR. Species matched IgG (Santa Cruz) was used as control for all ChIP experiments.

ChIP-re-ChIP was performed with 40 × 10^6^ cells per reaction. The first IP using the MYC antibody was described above. After washes, chromatin was released using Release Buffer (TE pH 7.5, 1% SDS, 10 mM DTT) at 37 °C for 30 min and diluted in Dilution Buffer with pre-incubated mixture of Dynabeads and MAX (73C5a, Abcam) or PNUTS antibodies or IgG. Following washes and decrosslinking, DNA was purified and analyzed by qPCR.

### Immunoprecipitation

Cell lysate was incubated with MYC (9E10, homemade) or IgG (sc-2027, Santa Cruz Biotechnology) antibodies with 15 μL protein G-sepharose beads (GE Healthcare) overnight to end-over-end rotation at 4 °C. Next day, beads were pelleted (425 × *g* for 2 min at 4 °C) and washed three times with 0.1% NP40 in PBS. The IP beads were resuspended in suitable buffers as described.

### 2D electrophoresis and immunoblotting

MYC was immunoprecipitated as described above and the MYC IP beads then resuspended in rehydration buffer (8 M Urea, 2% CHAPS, 25 mM DTT, 20 µg/mL IPG buffer pH 4–7 (17-6000-86, GE Healthcare), bromophenol blue), centrifuged and 125 µL of supernatant incubated with immobiline drystrip pH 4–7, 7 cm (17-6001-10, GE Healthcare). The IPG strip was covered with drystrip cover fluid (17-1335-01, GE healthcare) overnight in a reswelling tray. Next day, IPG strips were focused on Ettan IPGphor 3 (GE healthcare) at 300 V for 30 min, 300–10,000 V for 3 h, and 10,000 V for 4 h. The IPG strips were then washed with equilibration buffer I (6 M Urea, 0.375 M Tris pH 8.8, 2% SDS, 20% glycerol, and 20 mg/mL DTT) and equilibration buffer II (6 M Urea, 0.375 M Tris pH 8.8, 2% SDS, 20% glycerol, and 25 mg/mL Iodoacetamide) for 10 min on shaker. The IPG strips were then rinsed with SDS PAGE running buffer and overlaid onto a 10% SDS-PAGE with 0.5% agarose. The SDS-PAGE and immunoblotting was conducted as described earlier^54^.

### Phosphatase treatment

MYC was immunoprecipitated as described above and then the MYC IP beads were treated with 12.5 U protein phosphatase 1 (P07545, New England BioLabs) in NEB buffer and MnCl_2_ or 12.5 U CIP (M0290L, New England BioLabs) in NEB buffer 3 for 2 h at 30 °C. The beads were washed once with 0.1% NP40 in PBS, eluted with 2X sample buffer, incubated at 95 °C for 5 min, and loaded onto a 10% SDS-PAGE. To measure enzyme activity 1 μL of the CIP or 5 μL of the PP1 sample was removed at the end of the 2 h incubation period and prior to bead washing. These samples were then incubated with the substrate 50 mM PNP (P0757, New England BioLabs) in the above mentioned buffer for CIP or PP1 for 5 min or 30 min respectively at 30 °C in a 50 μL reaction volume. The reaction was stopped with 1 mL 1 N NaOH and the amount of *p-*Nitrophenol formed was determined by spectrophotometer absorbance at 405 nM (molar extinction coefficient 18,000 M-1 cm-1).

### Cellular fractionation

Cells grown in a tissue culture dish were washed once with PBS, scraped, collected into eppendorf tubes, and centrifuged at 1400 rpm for 2 min at 4 °C. Cell pellets were then resuspended in buffer A (10 mM HEPES pH 7.9, 10 mM KCl, 0.1 mM EDTA, 0.1 mM EGTA, 1 mM DTT, protease inhibitor cocktail (P8340, Sigma-Millipore), incubated on ice for 15 min, vortexed, and centrifuged at 3000 rpm for 5 min at 4 °C. The supernatant (cytoplasmic fraction) was collected in an eppendorf tube and pellet (nuclear fraction) was washed once with buffer A, centrifuged at 3000 rpm for 5 min at 4 °C. The pellet was lysed in SDS lysis buffer (0.05 M Tris pH 6.8, 1% SDS, 10% β-mercaptoethanol, and 10% glycerol) and passed through 25 G needle syringe a few times. The cytoplasmic fraction was also mixed with 4 × SDS lysis buffer. The nuclear and cytoplasmic extracts were heated at 95 °C for 5 min and loaded onto an SDS-PAGE.

### Antibodies, primers, and siRNAs

Antibodies for MYC pT58 (04-217, Millipore, used at 1:2000), MYC p62 (ab78318, abcam, used at 1:1000), MAX for ChIP (sc-765, Santa Cruz, 10 µg used for ChIP), PP1α isoform (438100, Invitrogen, used at 1:2000), PP1β isoform (ab53315, abcam, used at 1:1000), PP1γ isoform (A300-906A, Bethyl laboratories, used at 1:5000), biotin anti-mouse SV40 large T and small T antigen (554151, BD Biosciences, used at 1:1000), PNUTS for immunoblotting (A300-439A, Bethyl Laboratories, used at 1:15,000 to 1:20,000), MYC for ChIP (homemade N262, 2 µg used for ChIP, 4 µg used for re-ChIP), PNUTS for ChIP (A300-440A, Bethyl Laboratories, 4 µg for ChIP, 10 µg for re-ChIP), Actin (A2066, Sigma, used at 1:10000), Anti-mouse HRP (NA931V, GE healthcare, used at 1:10,000), Anti-tubulin (DM1A, Calbiochem, used at 1:2000), Anti-laminB1 (ab16048, Abcam, used at 1:1000) and Anti-rabbit HRP (NA934V, Sigma, used at 1:10,000).

The qPCR primers for PP1α isoform (fwd GTTCCTCCACAAGCACGACT, rev GTTCCTCCACAAGCACGACT), PP1β isoform (fwd GAAGATCTTCTGTTGTCATG, rev GCACATCCTTATCTGGATCAGAC), PP1γ isoform (fwd ACTAGAACTTGAAGCACCACT, rev CGCAGCAAATCATAGTATTGTCC), and RPLPO (fwd CAGATTGGCTACCCAACTGTT, rev GGGAAGGTGTAATCCGTCTCC). The qPCR primers for ChIP for CAD (fwd ACGTGGACCGACTCCGG, rev CCATGGGAAGGGAACTCAGA), CDK4 (fwd AGGCATGTGTCATGTGTGATCTT, rev CCGCTCCCAGTCTTCCTTG, HES1 (fwd AATGAGATCCGGAATCGGCG, rev TCATCCGTAGGCTTTAGGTTCTG), LDHA (fwd ACGTCAGCATAGCTGTTCCA, rev AATGAGATCCGGAATCGGCG) NCL (fwd TTGCGACGCGTACGAGCTGG, rev ACTCCGACTAGGGCCGATAC), *PPP1R10* (fwd TGCTAGTGAAACGCCCTTGT, rev GCCAATCAGTTGGCGAGTTG), Chr6 (fwd TGGCATTGTCCTAATACTTCAGTGAT, rev TTTCTGAAGTGCTGCTACCTCTCA)

The protein phosphatase catalytic subunit siRNAs were from OriGene; *PPP1CA*- SR303671A (GAGACGCUACAACAUCAAACUGUGG) and SR303671B (AGACGGCUACGAGUUCUUUGCCAAG), *PPP1CB*- SR303672A (CUAAUAGAAAGAUGUGCUACACUGT) and SR303672B (GCUUUGUAGUGAAGUAUAGUAGCAA), *PPP1CC*- SR303673A (GCUUCAGGAGAAUGAAAUCAGAGGA) and SR303673B (CGAUGGCGGAUUUAGAUAAACUCAA).

### Co-amplification heatmap

The occurrence of co-amplification of *MYC* with *PPP1R10, PPP1CA, PPP1CB*, and *PPP1CC* was assessed in multiple cancer types using datasets provided by The Cancer Genome Atlas (TCGA). DNA genome-wide somatic copy number and clinical profiles were downloaded from Broad GDAC Firehouse (https://gdac.broadinstitute.org/), release 2014-10-17. GISTIC v223 level 4 data was used for somatic copy-number analysis and all data pre-processing was completed using the R statistical environment (v.3.1.3). The Chi-square test of independence was applied to test the dependence of *PPP1R10* amplification, along with PP1 subunits, on *MYC* amplification using the R statistical environment (v.3.2.5). All visualizations were completed using lattice (v.0.20-33) and latticeExtra (v.0.6-28).

### Statistical analysis

All the graphs show median with range or mean with error bars representing s.d. of a minimum of three biological replicates. Differences in groups for bar graphs were analyzed using one-way ANOVA with Bonferroni test or for grouped bar graphs using two-way ANOVA with Bonferroni test using Graphpad Prism Software. For BioID data, proteins identified with a cut-off of 0.8 using ProteinProphet (corresponding to FDR < 1%) were analyzed with SAINT Express v. 3.3^50,51^.

### Data availability

The BioID and PTM mass spectrometry datasets generated and analysed during the current study are available in the MassIVE repository (accession number: MSV000082424). The data that support the findings of this study are available from the corresponding author upon request.

## Electronic supplementary material

Supplementary Information
