# Supplementary material for: MYC dephosphorylation by the PP1/PNUTS phosphatase complex regulates chromatin binding and protein stability
Source: Nat Commun. 2018 Aug 29;9:3502. doi: 10.1038/s41467-018-05660-0 (PMC6115416; doi:10.1038/s41467-018-05660-0)
Supplement: Supplementary file 1 — Supplementary Information [file 41467_2018_5660_MOESM1_ESM.pdf]

Supplementary Dataset

MYC dephosphorylation by the PP1/PNUTS phosphatase complex regulates chromatin binding and protein stability

Dingar *et al.*

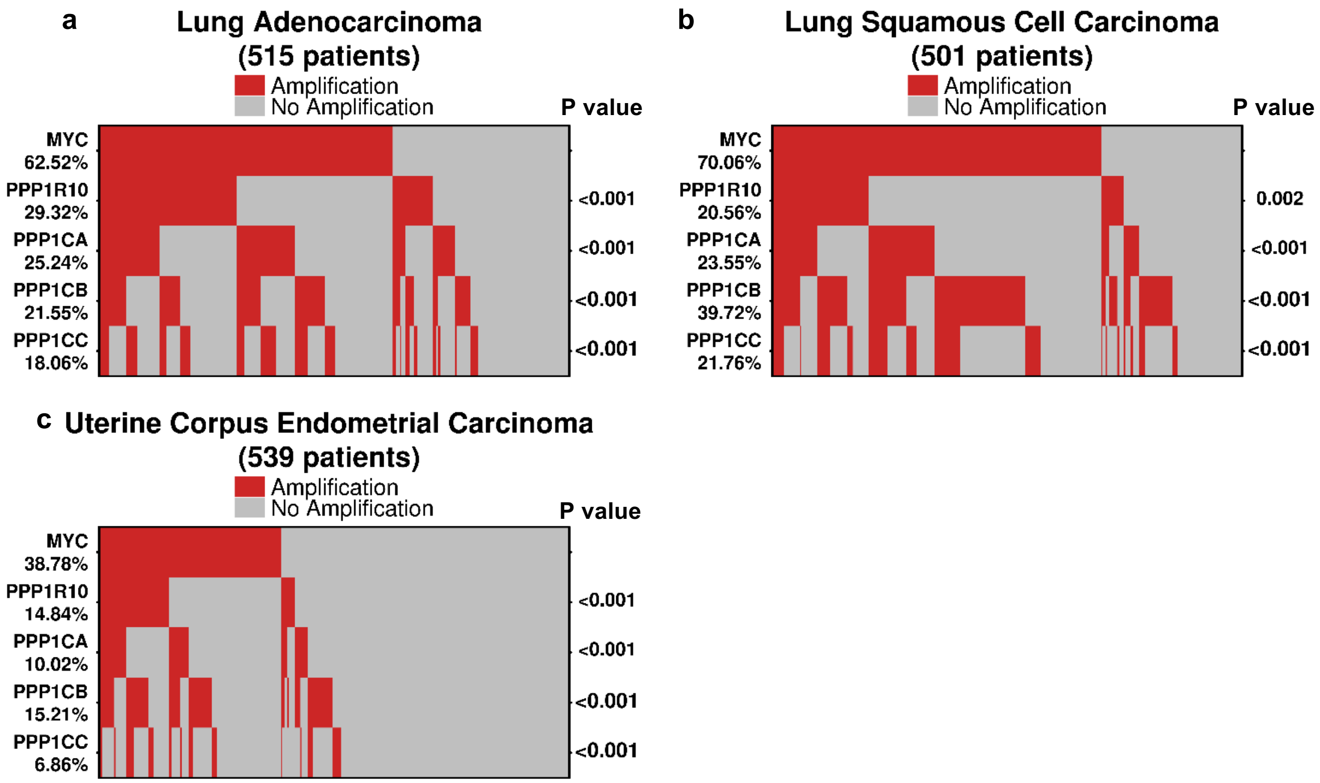

Supplementary Figure 1. MYC and PP1/PNUTS gene amplification in cancer. *MYC* and *PPP1R10*, *PPP1CA*, *PPP1CB*, or *PPP1CC* gene amplification is evident and demonstrates co-occurrence in (**a**) lung adenocarcinoma, (**b**) lung squamous cell carcinoma, and (**c**) uterine corpus endometrial carcinoma.

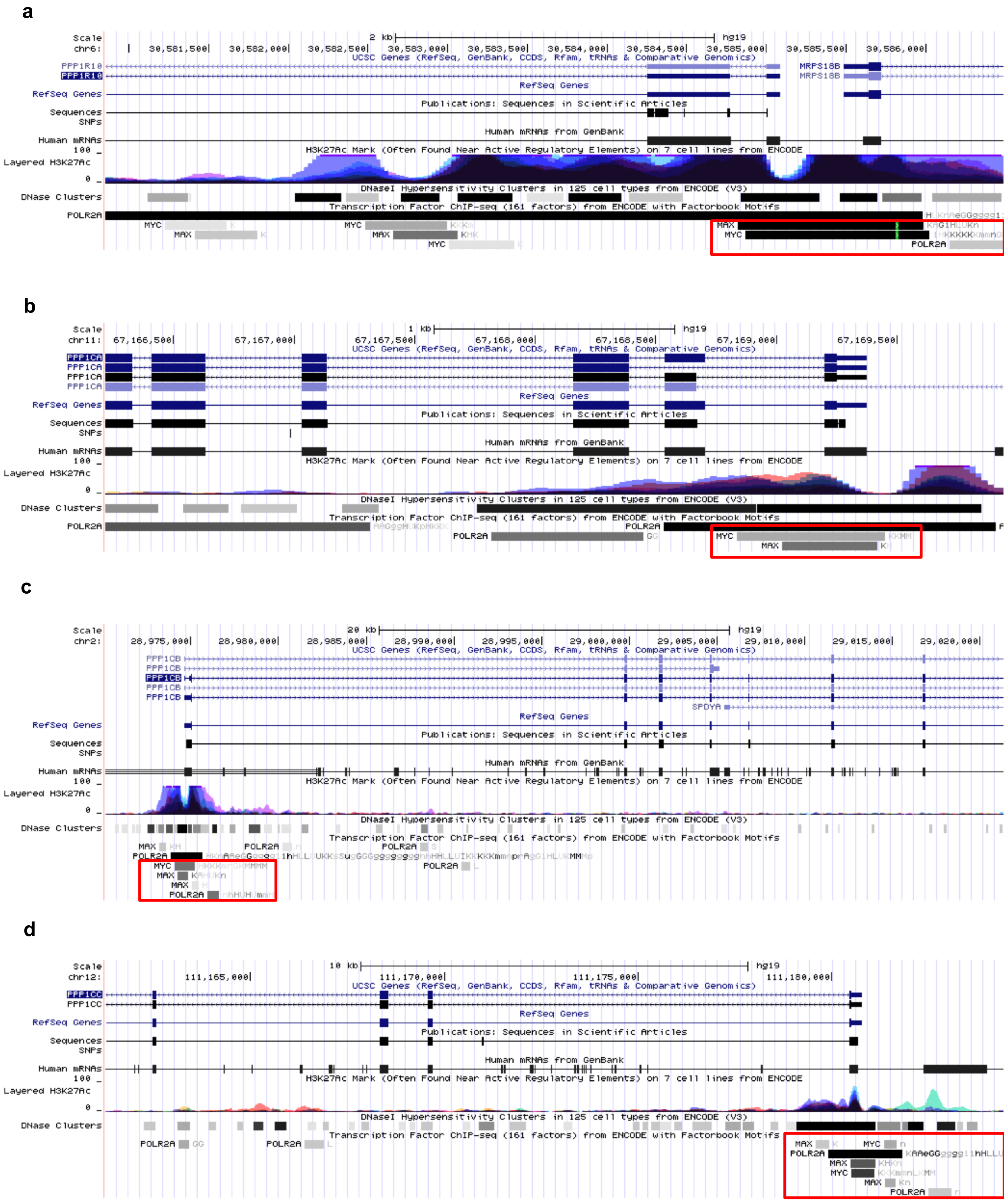

Supplementary Figure 2. MYC binding to *PPP1R10*, *PPP1CA*, *PPP1CB*, and *PPP1CC* gene promoters. Analysis of MYC ChIP-seq data set from ENCODE shows binding of MYC and MAX to (**a**) *PPP1R10*, (**b**) *PPP1CA*, (**c**) *PPP1CB*, and (**d**) *PPP1CC* gene promoters. Red boxes show regions of MYC and MAX binding, and green vertical bar shows the E-box motif. POLR2A (RNA Polymerase II Subunit A) is one of the subunits of RNA polymerase II, which is involved in gene transcription.

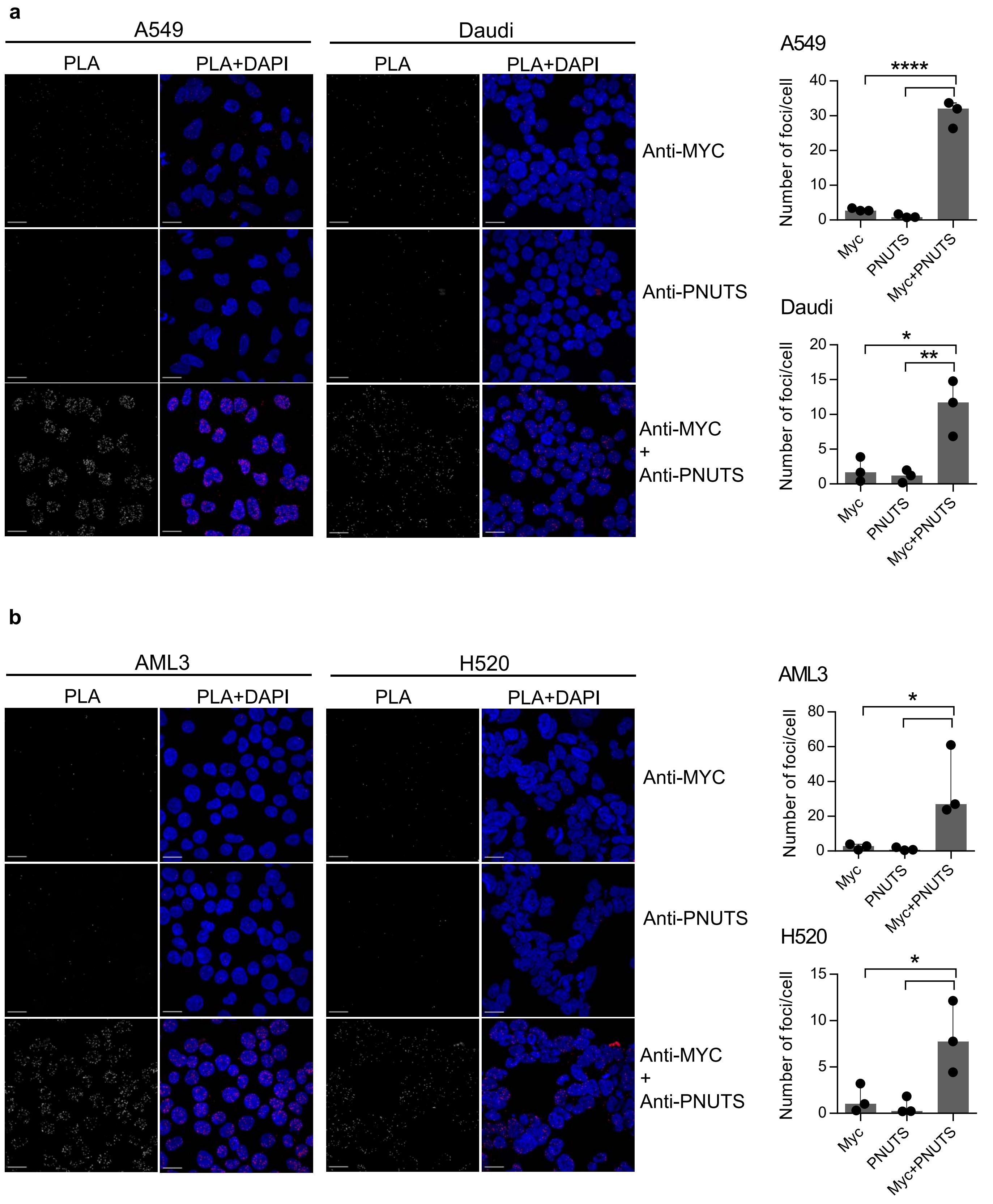

Supplementary Figure 3. MYC and PNUTS interact in multiple cell types. (**a**) A549 (left panel), Daudi (right panel) (**b)** AML3 (left panel), H520 (right panel) cells were fixed, permeabilized, blocked with BSA, and probed with a primary MYC rabbit antibody and/or a PNUTS mouse antibody. The proximity of MYC and PNUTS was assayed using the Duolink Proximity Ligation Assay (PLA) In Situ Red Starter kit (Sigma) as per manufacturer's instructions. Shown are representative images of n=3. PLA signal (white or red) and nuclear staining (DAPI) (blue) are shown for cells probed with anti-MYC alone (top row), anti-PNUTS alone (middle row), or anti-MYC and anti-PNUTS (bottom row). PLA signal was quantified and shown as median number of foci per cell with range (n=3). * $p<0.05$, ** $p<0.01$, **** $p<0.0001$, one-way ANOVA with Bonferroni test. Scale bars represent 20μm.

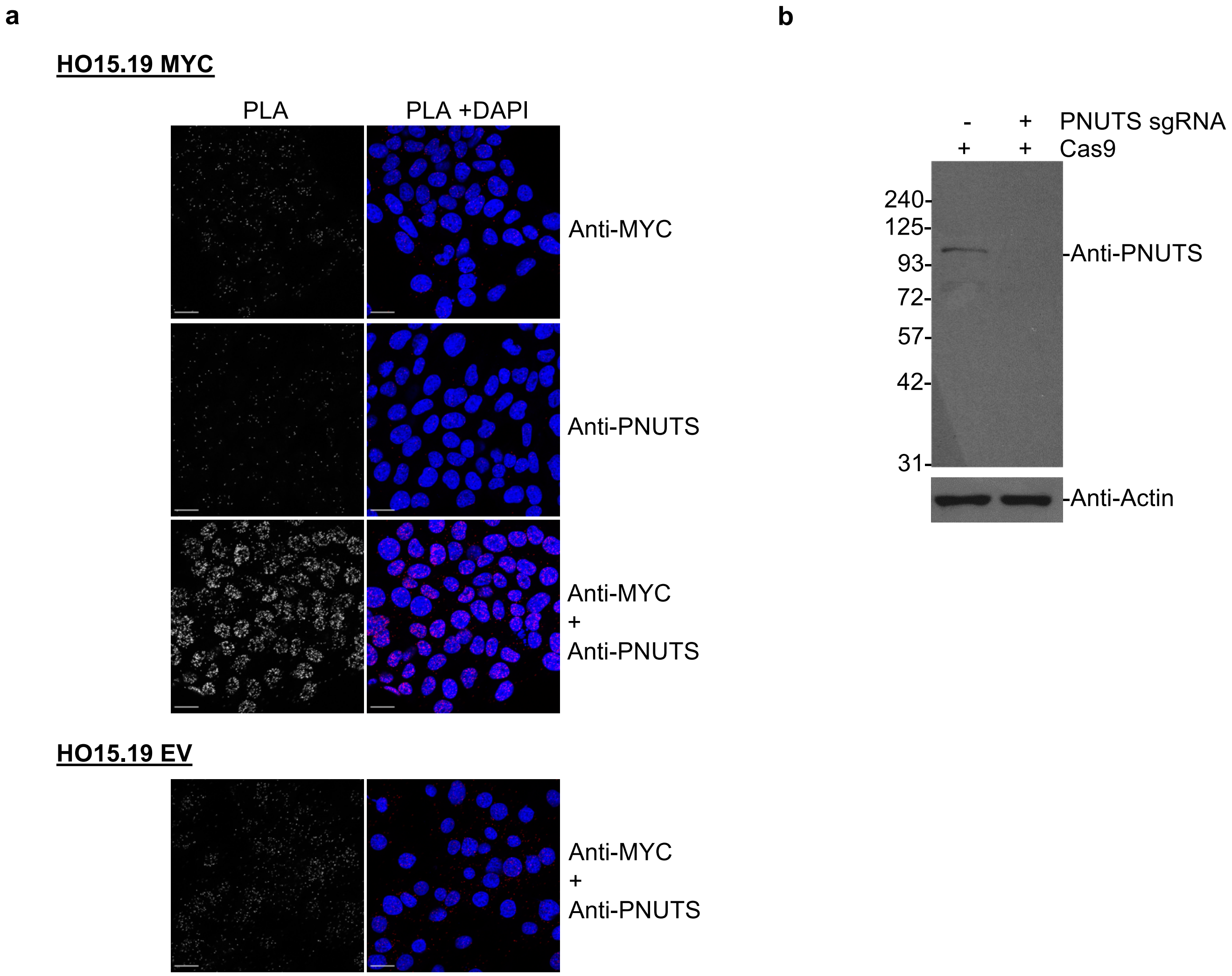

Supplementary Figure 4. MYC and PNUTS interact in HO15.19 cells. (**a**) HO15.19 MYC knockout or ectopic MYC expressing cells were fixed, permeabilized, blocked with BSA, and probed with a primary MYC rabbit antibody and/or a PNUTS mouse antibody. The proximity of MYC and PNUTS was assayed using the Duolink PLA In Situ Red Starter kit (Sigma) as per manufacturer's instructions. Shown are representative images of n=3. PLA signal (white or red) and nuclear staining (DAPI) (blue) are shown for cells probed with anti-MYC alone (top row), anti-PNUTS alone (middle row), or anti-MYC and anti-PNUTS (bottom row). Scale bars represent 20µm. (**b**) HEK293 cells were transfected with the empty PX459 vector containing Cas9 or PX459 with PNUTS sgRNA plasmid by calcium phosphate and cells selected with 1 µg/mL Puromycin. Antibiotic resistant cells were grown in tissue culture and lysed after 48 hours. Protein lysate (20 µg) was separated on 10% SDS-PAGE, transferred onto nitrocellulose membrane, and immunoblotted with PNUTS mouse or actin antibody. Error bars represent s.d.

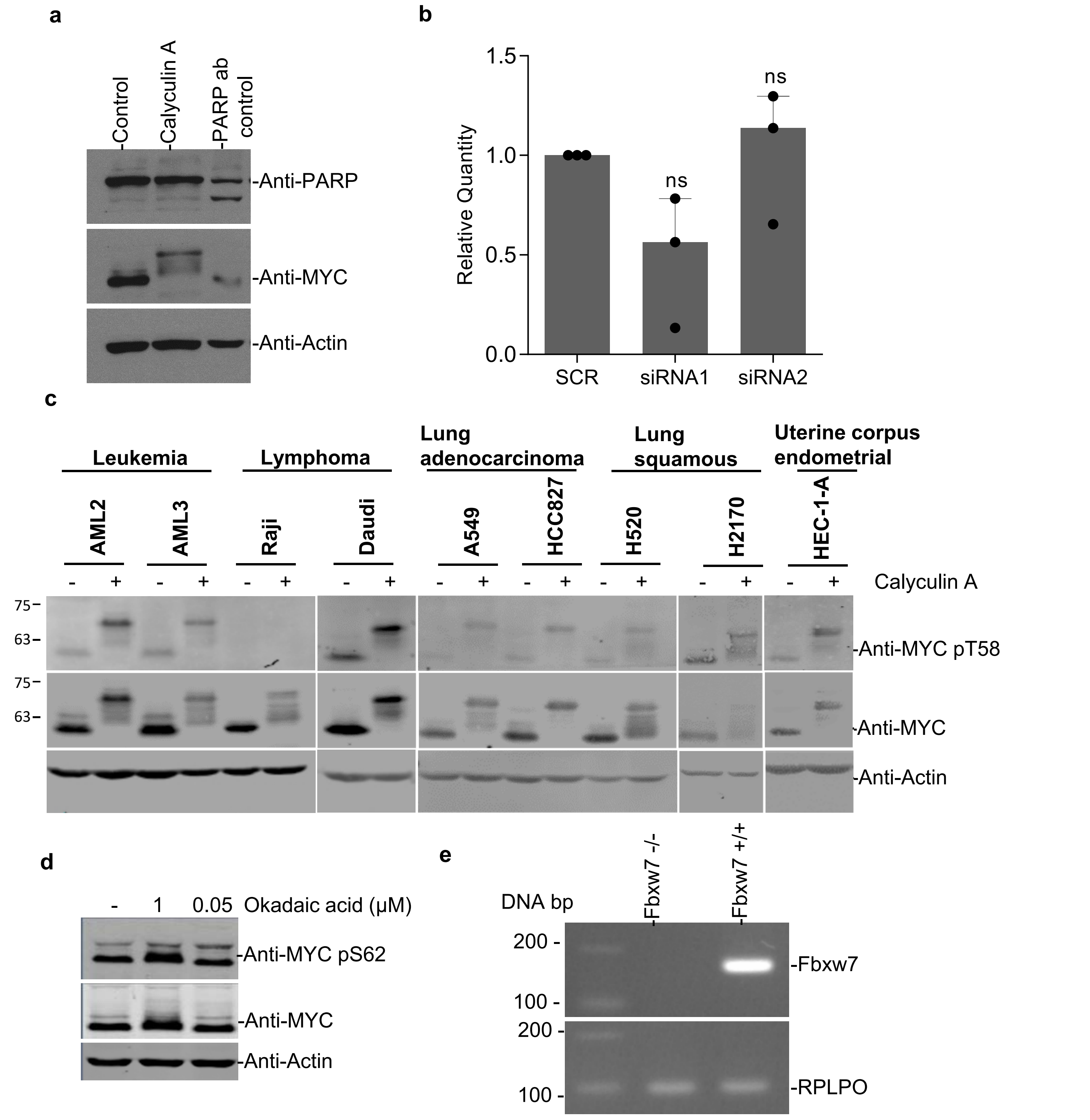

Supplementary Figure 5. Calyculin A does not trigger PARP cleavage, PP1 knockdown does not affect MYC mRNA level, and Calyculin A but not other phosphatase inhibitors triggers MYC hyperphosphorylation and degradation across multiple cell types. (**a**) MCF10A cells were treated with DMSO or Calyculin A (50 nM) for 30 minutes, and lysed. MCF10A lysate or apoptotic cell lysate used as a positive antibody control were separated on 10% SDS PAGE and immunoblotted with indicated antibodies. (**b**) Growing MCF10A cells were transfected with 10 nM siRNA directed against PP1α, β, and γ or universal scrambled negative control by calcium phosphate, then lysed in TRIzol after 48 hours, RNA was extracted, cDNA prepared and qPCR performed with gene specific primers. *RPLP0* was used as an internal control. Shown are relative quantity with median and range (n=3). ns: not significant, one-way ANOVA with Bonferroni test. (**c**) AML2, AML3, Raji, Daudi, A549, HCC827, H520, H2170, or HEC-1-A cells treated with DMSO or Calyculin A (50 nM) for 30 minutes, lysed, and immunoblotted with indicated antibodies. (**d**) MCF10A cells were treated with vehicle control or okadaic acid 1 μM for 30 minutes or 50 nM for 4 hours, lysed, and immunoblotted with indicated antibodies. (**e**) The *FBXW7* knockout status in HCT116 cells was validated by PCR with primers designed to the deleted exon region. HCT116 *FBXW7* knockout (-/-) or wildtype (+/+) cells were lysed, genomic DNA extracted, and PCR performed with *FBXW7* or *RPLP0* gene specific primers. PCR products were separated on a 3% agarose gel and imaged using a gel doc system.

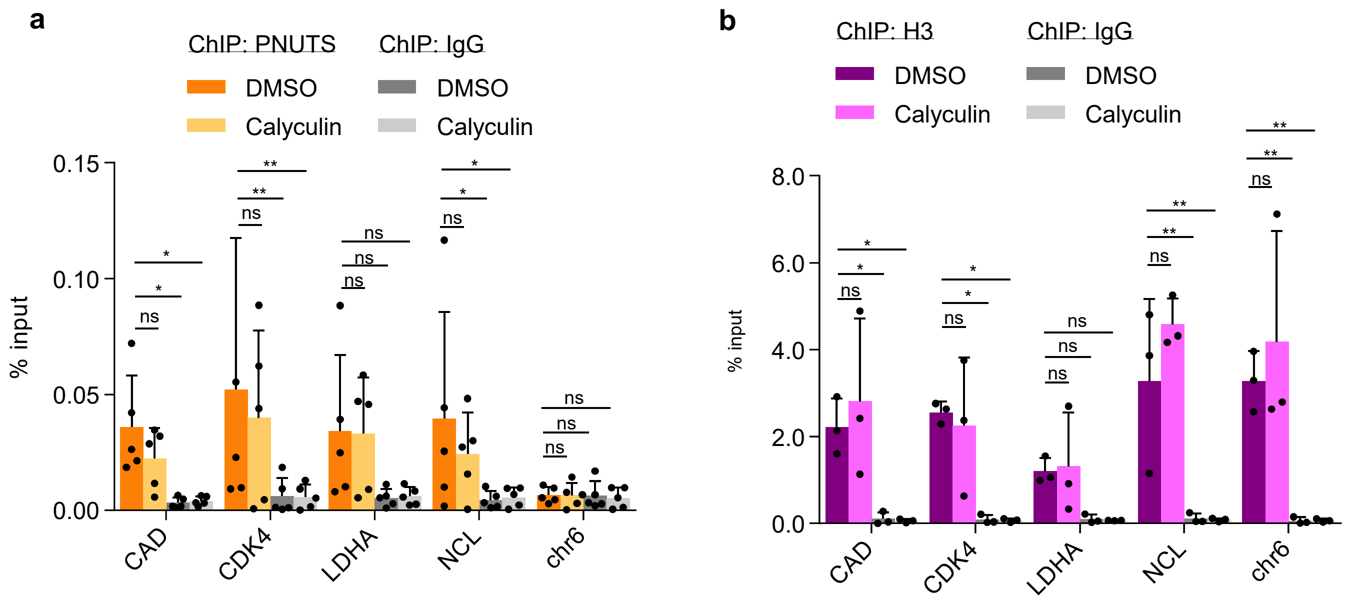

Supplementary Figure 6. PNUTS binding or chromatin integrity is not changed with Calyculin A treatment. For ChIP analysis, MCF10A cells were treated with MG132 (10 μM) for 4 hours ± Calyculin A (50 nM) for 30 minutes, and ChIP analysis performed with (**a**) PNUTS or (**b**) Histone H3 antibody or IgG antibody as a control. ChIP'd DNA was quantified for MYC target genes promoters, *CAD, CDK4, LDHA*, and *NCL*, and chr6 (negative control). Shown are % input mean (PNUTS ChIP: n=5; Histone ChIP: n=3). * p<0.05, ** p<0.01, *** p<0.001, **** p<0.0001, two-way ANOVA with Bonferroni test. Error bars represent s.d.

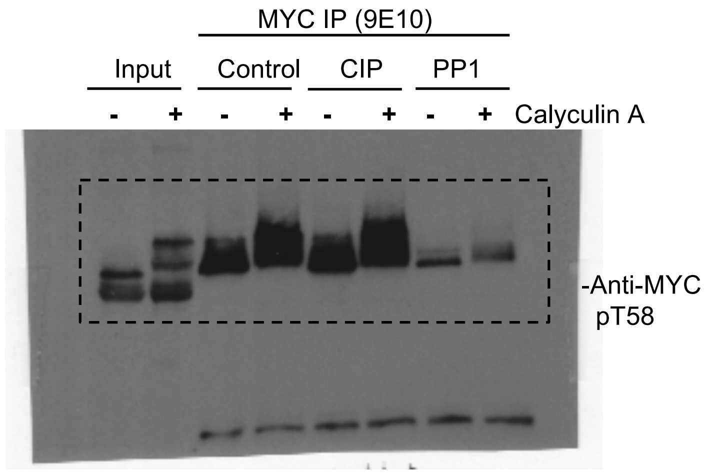

Figure 4A

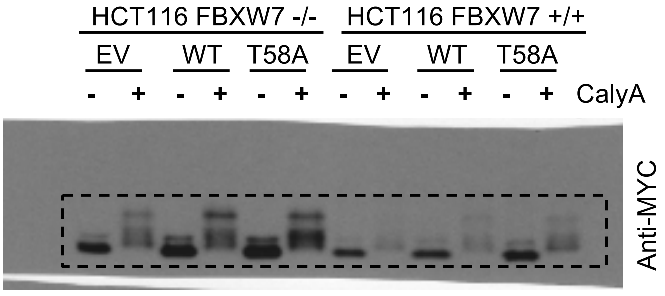

Figure 4B

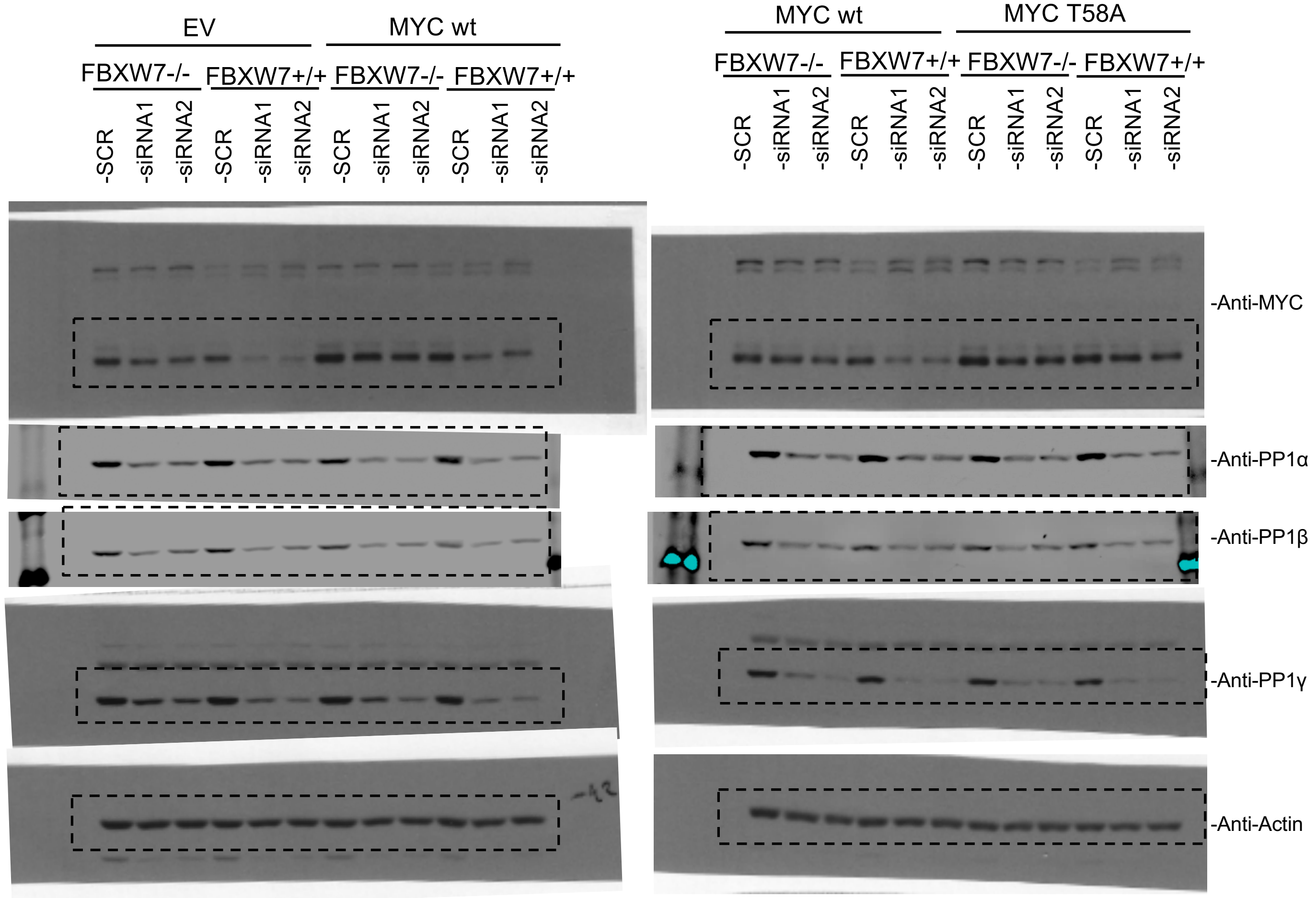

Figure 4C

Supplementary Figure 7 - Original Western blots for Figure 4. Anti-MYC pT58 blots from Figure 4A and Anti-MYC blots from Figure 4B. Anti-MYC, PP1α, PPIβ, PPIγ, and actin blots from Figure 4C.

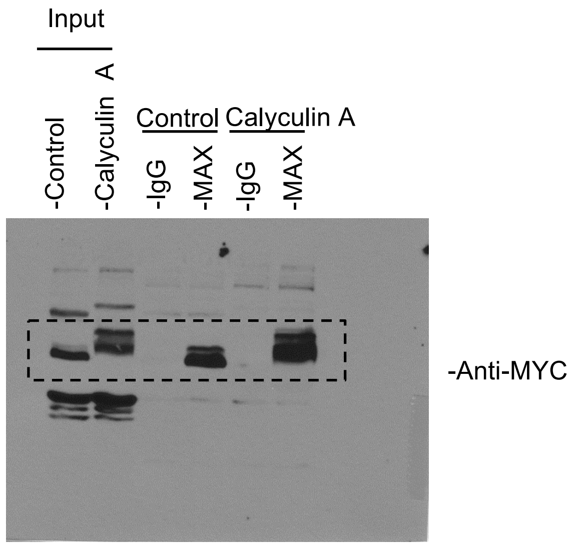

Figure 6A

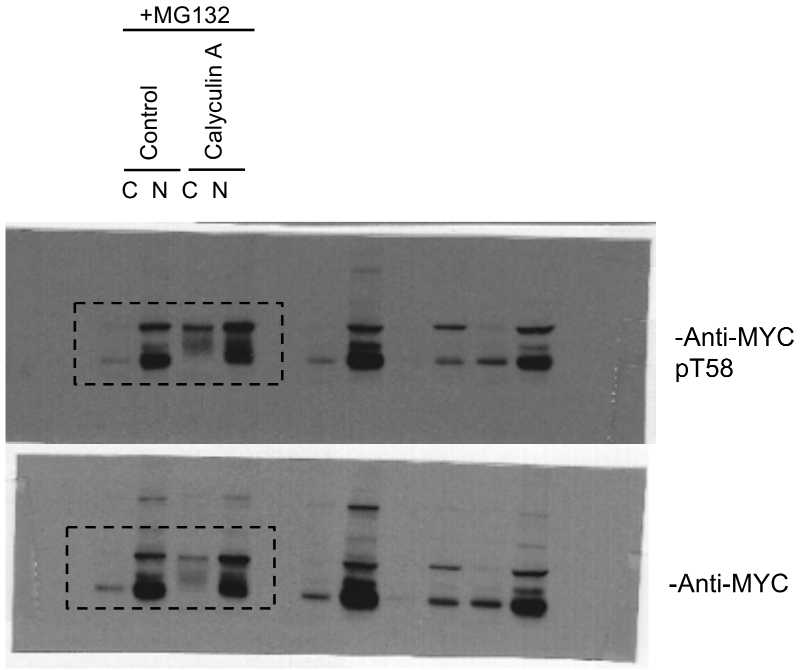

Figure 6C, Left

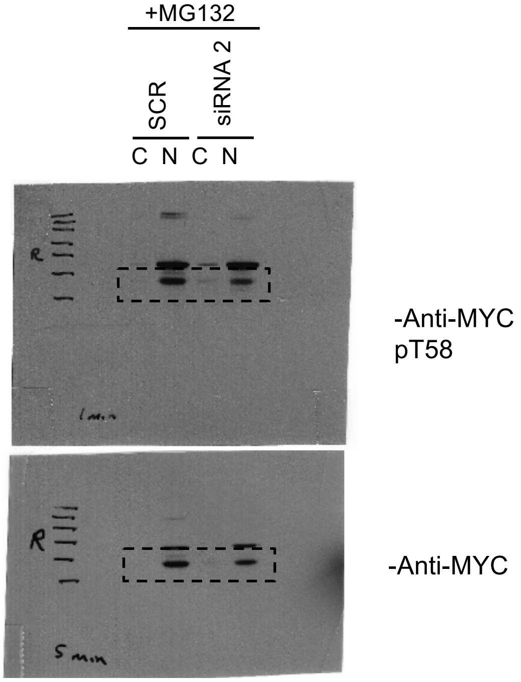

Figure 6C, Right

Supplementary Figure 8 – Original Western blots for Figure 6. Anti-MYC blot from Figure 6A. Anti-MYC pT58A and Anti-MYC blots from Figure 6C.

Supplementary Table 1: raw BioID interactome data for MYC transcription factor. (**a**) High-confidence proximity interactors for MYC transcription factor (TPP> 0.8, unique peptides ≥2, FDR ~1%). Table containing data from two technical and two biological replicates, which were analyzed for the bait and compared to controls (FlagBirA* alone, used for SAINT analysis). (**b**) Unfiltered summary of all the peptide counts derived via search engine TTP XTandem and protein-associated SAINT score are shown.

Supplementary Table 1 a.

				MYC 4609	2573 N-FlagBirA-HeLa_ONLY_Ctrl_B_v1(N-FlagBirA) 460	2574 N-FlagBirA-HeLa_ONLY_Ctrl_B_v2(N-FlagBirA) 460	2575 N-FlagBirA-HeLa_ONLY_Ctrl_C_v1(N-FlagBirA) 460	2576 N-FlagBirA-HeLa_ONLY_Ctrl_C_v2(N-FlagBirA) 460	2546 N-FlagBirA-HeLa_Myc_WT_B_v1(N-FlagBirA) 4609	2547 N-FlagBirA-HeLa_Myc_WT_B_v2(N-FlagBirA) 4609	2548 N-FlagBirA-HeLa_Myc_WT_C_v1(N-FlagBirA) 4609	2549 N-FlagBirA-HeLa_Myc_WT_C_v2(N-FlagBirA) 4609
Gene ID	Gene Name	LocusTag	Protein ID	SaintScore	Total Peptide	Total Peptide	Total Peptide	Total Peptide	Total Peptide	Total Peptide	Total Peptide	Total Peptide
8295	TRRAP		4507691	1					305	255	224	223
57634	EP400		56549696	1					294	252	251	223
4288	MKI67		103472005	1	46	29	28	18	167	129	110	114
56922	MCCC1		116805327	1	36	30	29	12	146	131	116	112
3054	HCFC1		98986457	1	23	15	13	5	133	108	98	105
1108	CHD4		51599156	1	31	20	17	5	133	100	92	80
3313	HSPA9		24234688	1	29	20	18	9	100	90	87	78
1912	PHC2		37595528	1					87	57	31	27
23394	ADNP		12229217	1	13	11	7		84	54	63	55
221037	JMJD1C		118600981	1	12	9	7	3	82	63	72	59
64087	MCCC2		11545863	0.99	26	26	16	9	75	63	67	64
10594	PRPF8		91208426	1	8	8			70	56	54	49
23451	SF3B1		54112117	1	22	16	11	6	69	56	48	41
10992	SF3B2		55749531	1	16	8	12	8	66	57	52	39
498	ATP5A1		4757810	1	25	20	19	9	66	61	58	56
23269	MGA		256017163	1					65	48	55	42
10009	ZBTB33		296179376	1	16	11	17	10	64	51	51	49
57680	CHD8		282165704	1					62	44	54	50
9188	DDX21		50659095	1	19	11	5	7	60	50	46	40
5411	PNN		33356174	1	21	15	11	2	59	58	47	43
9092	SART1		10863889	1	12	14	11	4	58	52	41	40
5514	PPP1R10		25777671	0.99	19	7	12	6	57	40	34	37
7284	TUFM		34147630	1	16	15	7	3	53	49	35	34
1662	DDX10		13514831	1					52	48	49	36
4670	HNRNPM		14141152	1	15	13	6	2	49	36	34	31
8607	RUVBL1		4506753	1	15	15	14	6	48	46	38	35
55929	DMAP1		13123776	1					47	34	36	34
56946	C11orf30		19923559	1					46	37	38	34
23020	SNRNP200		40217847	1	12				45	44	27	31
80314	EPC1		13376810	1					44	36	41	36
57805	KIAA1967		24432106	1	7				44	32	28	23
871	SERPINH1		32454741	1	13	11	13	6	44	29	29	20
6599	SMARCC1		188536047	1	7	3			42	24	31	31
2975	GTF3C1		101943240	1					42	37	25	31
292	SLC25A5		156071459	1	13	10	11	5	41	36	29	32
1665	DHX15		68509926	0.99	17	11	8		41	38	33	34
54439	RBM27		168229174	1	8		9		40	33	29	27
731751	LOC731751		113430845	1	9				39	26	25	
8473	OGT		32307148	1	5				39	26	20	22
27339	PRPF19		7657381	1	7	9	10	2	38	29	32	24
5394	EXOSC10		4505917	1	2				38	27	21	20
2969	GTF2I		14670350	1	7	11	7	3	38	36	26	33
29889	GNL2		7019419	1	10	7	6		37	24	28	26
9878	TOX4		7662274	0.98	13	11	13	3	37	33	27	26
23450	SF3B3		54112121	1	15	13	10	8	35	33	27	26
6597	SMARCA4		192807312	1	5	5	3		35	32	36	33
3550	IK		125988409	1	2	7	4	3	35	35	26	24
988	CDC5L		11067747	1	8	4	4		35	25	21	28
506	ATP5B		32189394	1	13	10	8	4	35	32	28	29
9343	EFTUD2		217272892	0.99	12	8	4	3	35	23	15	13
23223	RRP12		223278379	1					34	23	28	20
10524	KAT5		36287049	1					34	28	36	28
56949	XAB2		55770906	1	5	3	4	5	33	32	19	15
1655	DDX5		4758138	1	9	9	9	7	33	28	27	22
57062	DDX24		9966805	1					32	30	23	21
9219	MTA2		14141170	1	6	7	7	4	32	29	25	27
3065	HDAC1	f	13128860	1	3				32	23	23	15
6124	RPL4		16579885	1	10	10	7		31	23	24	24
117246	FTSJ3		194097365	1		2			30	27	28	27
55388	MCM10		33383235	1					30	28	31	20
54908	CCDC99		157388975	1	5	4	6	3	30	18	22	20
23309	SIN3B		52138513	1		2			30	26	24	22
3301	DNAJA1		4504511	1	3	5	4	4	30	25	21	21
86	ACTL6A		30089997	1					30	23	21	13
55689	YEATS2		33620755	1					29	28	25	21
23272	FAM208A		163838631	1					29	18	17	15
10128	LRPPRC		31621305	1	4				29	17	21	19

4691	NCL		55956788	1	7	6	7		29	32	26	31
1660	DHX9		100913206	1	14	7	7	5	29	32	24	24
10813	UTP14A		21361348	1					28	26	29	19
6594	SMARCA1		164419749	1					28	20	19	
56897	WRNIP1		18426902	1					26	28	30	28
6950	TCP1		57863257	1	4	2	11	3	26	28	29	28
22938	SNW1		6912676	1	7	7	5	5	25	27	21	20
10856	RUVBL2		5730023	1	4	5	3		25	17	20	15
9875	URB1		194394141	1					25	22	15	15
6122	RPL3		4506649	1	9	8	7	3	25	18	25	16
55791	LRIF1		55743124	1					24	28	25	19
5725	PTBP1		14165464	1	3	3	5	3	24	20	16	11
55636	CHD7		54112403	1	4		2		23	20	20	18
51602	NOP58		7706254	1	3	3	3		23	19	22	13
30836	DNTTIP2		54633315	1	3	2	2		23	24	22	20
25942	SIN3A		223941782	1	3				23	18	18	12
6191	RPS4X		4506725	1	3	4			23	14	20	15
5832	ALDH18A1		21361368	1	4	2	5		23	23	17	13
11176	BAZ2A		91176325	0.99	7	8	3		23	19	18	13
26122	EPC2		194272186	1					22	19	18	19
23560	GTPBP4		55953087	1					22	12	19	20
6138	RPL15		15431293	1	2	3	4		22	15	16	17
26354	GNL3		45593130	1	3	2			21	17	15	19
7385	UQCRC2		50592988	1	5	4			21	11	17	15
6130	RPL7A		4506661	1	5	7	6	3	21	20	19	19
2547	XRCC6		4503841	1					21	16	13	14
259266	ASPM		126116596	1					20	19	13	10
134430	WDR36		21281677	1	2		3		20	19	19	17
81608	FIP1L1		201023339	1	9	6	5	2	20	23	16	16
55299	BRIX1		55770900	1	3				20	15	17	12
10199	MPHOSPH10		31317305	1					20	13	15	15
8402	SLC25A11		21361114	1	7	4	6		20	19	13	14
64324	NSD1		19923586	1					19	12	11	11
10399	GNB2L1		5174447	1	9	7	5	3	19	19	20	14
9584	RBM39		35493811	1	6	4			19	16	17	17
6605	SMARCE1		21264355	1		2	3		19	16	17	15
6601	SMARCC2		194363725	1					19	9	17	14
654364	NME1-NME2		66392203	1					18	11	11	12
23528	ZNF281		6912752	1					18	18	16	18
23521	RPL13A		6912634	1	6	4	5		18	14	14	12
22803	XRN2		18860916	1					18	15	11	13
10523	CHERP		119226260	1	3	2			18	10	9	8
10445	MCRS1		29893564	1					18	12	18	13
10147	SUGP2		224282117	1	4				18	17	6	3
9330	GTF3C3		6912398	1					18	15	11	6
6202	RPS8		4506743	1	7	4	6	4	18	15	19	21
6129	RPL7		15431301	1	9	4	3	2	18	16	10	16
4149	MAX		21704263	1					18	14	13	16
54880	BCOR		183396783	1					17	9	15	14
6944	VPS72		5174715	1					17	16	14	12
2023	ENO1		4503571	1	6	5	4		17	18	17	11
1398	CRK		41327712	1	5				17	14	17	14
904	CCNT1		17978466	1	2		2		17	14	10	11
5478	PPIA		10863927	0.99	8	4	4		17	17	18	16
5315	PKM2		33286418	0.99		5			17	10	9	
157313	CDCA2		44681484	0.96	8	6	5		17	19	15	16
79718	TBL1XR1		19913371	1					16	11	8	16
79577	CDC73		40018640	1					16	15	15	14
58525	WIZ		151301215	1					16	11	14	16
10949	HNRNPA0		5803036	1	2	2	3		16	18	15	15
8089	YEATS4		5729838	1					16	16	15	15
6879	TAF7		14717407	1	4	2	2	3	16	11	10	6
6125	RPL5		14591909	1	6	6	2	2	16	16	17	15
3096	HIVEP1		116805342	1					16	20	10	12
2091	FBL		12056465	1					16	14	15	14
10236	HNRNPR		156151392	0.99	6				16	10	6	7
132660	LIN54		169234719	1					15	10	11	11
83746	L3MBTL2		20149698	1					15	12	12	16
64318	NOC3L		20806097	1					15	10	13	12
57167	SALL4		10047144	1					15	14	10	9
51366	UBR5		15147337	1					15	8	10	8
24144	TFIP11		56788356	1					15	11	6	4
6184	RPN1		4506675	1	4	3			15	15	14	8
1107	CHD3		158420731	1					15	16	11	11
10574	CCT7		261399877	0.98	4				15	7	4	7
57187	THOC2		125656165	0.97	7	3			15	12	12	8
57459	GATAD2B		21218438	1					14	12	13	9
55210	ATAD3A		283436222	1					14	12	16	16
51575	ESF1		18093112	1					14	12	9	10
9877	ZC3H11A		114842410	1					14	10	13	8
6185	RPN2		209413738	1	3				14	12	10	11
4999	ORC2		5453830	1			3		14	10	7	4
205860	TRIML2		27734883	0.99	5	3	6		14	16	13	11
23279	NUP160		54859722	0.99					14	6	7	7
254225	RNF169		148839382	1					13	10	2	5
221504	ZBTB9		23308693	1					13	10	7	9
220988	HNRNPA3		34740329	1					13	9	6	3
57050	UTP3		9966799	1					13	8	10	14
54556	ING3		38201655	1					13	14	12	11
25926	NOL11		21361468	1	2				13	7	11	9
24149	ZNF318		120587019	1					13	9	8	7
24148	PRPF6		40807485	1					13	13	13	
23481	PES1		7657455	1	3				13	11	9	8
23186	RCOR1		7661892	1					13	11	12	8
23013	SPEN		14790190	1					13	8	8	5

10286	BCAS2		5031653	1	3	3			13	9	9	6
9349	RPL23		4506605	1					13	10	10	10
9328	GTF3C5		170763506	1					13	12	10	11
6209	RPS15		4506687	1					13	9	20	13
6144	RPL21		18104948	1	2	3	4	3	13	11	11	11
1452	CSNK1A1		68303572	1	2				13	15	12	9
79882	ZC3H14		231570121	0.99	5	5	3		13	16	12	10
55683	KANSL3		169234787	1					12	11	6	8
54881	TEX10		239787838	1					12	12	7	10
22984	PDCD11		70980549	1					12	3	5	5
10514	MYBBP1A		157694492	1					12	10	9	13
10165	SLC25A13		237649019	1					12	12	7	6
9212	AURKB		83776600	1			2		12	8	7	3
9128	PRPF4		24431950	1	2				12	7	6	6
9112	MTA1		115527080	1	3				12	12	10	7
7520	XRCC5		10863945	1					12		4	5
6201	RPS7		4506741	1					12	10	12	12
6169	RPL38		4506645	1		2	2		12	8	6	9
6136	RPL12		4506597	1	2		4		12	9	8	7
5931	RBBP7		4506439	1		4			12	6	9	15
488	ATP2A2		24638454	1	2		3		12	12	6	7
10389	SCML2		5174669	0.99	3	3			12	6	8	8
6228	RPS23		4506701	0.99	7	4	4	2	12	13	12	14
6208	RPS14		5032051	0.99	6	3	4		12	12	12	10
10527	IPO7		5453998	0.98					12	4	4	4
9532	BAG2		4757834	0.98	6	4	4		12	11	12	9
9169	SCAF11		117676384	0.98	3	5			12	13	9	11
6133	RPL9		15431303	0.98	4	3	5	3	12	12	11	11
103910	MYL12B		15809016	1					11	8	7	7
84991	RBM17		14249678	1		2			11	10	7	5
57798	GATAD1		88759346	1					11	6	4	4
55661	DDX27		224593278	1					11	9	15	13
54815	GATAD2A		164519146	1	3		2	2	11	13	11	12
51645	PPIL1		7706339	1					11	10	10	9
9790	BMS1		224589071	1					11	11	12	6
9329	GTF3C4		156119605	1					11	6	6	8
6189	RPS3A		4506723	1	6	3	3		11	9	13	13
6142	RPL18A		11415026	1	3	2	3		11	10	12	9
6117	RPA1		4506583	1					11	9	10	7
4839	NOP2		76150623	1					11	9	11	12
4297	MLL		308199413	1					11	12	7	7
2186	BPTF		38788260	1					11	12	7	8
11168	PSIP1		190014586	0.99	3	6	2		11	14	5	4
10432	RBM14		5454064	0.99	3	2			11	6	8	5
3619	INCENP		102467235	0.98	4				11	6	8	7
23469	PHF3		7662018	0.97		3	2		11	7	5	2
196528	ARID2		56549668	1					10	4	3	4
79915	ATAD5		26080431	1					10	4	5	2
56987	BBX		18378731	1					10	4	6	2
55646	LYAR		224591430	1					10		4	5
55193	PBRM1		30794372	1					10	9	3	4
29028	ATAD2		24497618	1					10	9	9	7
23246	BOP1		21327667	1					10	8	7	8
23165	NUP205		57634534	1					10	7	5	4
10969	EBNA1BP2		237649012	1					10	11	11	12
10724	MGEA5		11024698	1					10	10	9	7
7707	ZNF148		145386566	1					10	11	8	6
7690	ZNF131		84872173	1					10	4	8	3
7343	UBTF		115529449	1					10	9	8	8
6229	RPS24		14916501	1	4	2	2		10	10	10	10
5160	PDHA1		291084742	1					10	6	4	4
9757	MLL4		7662046	0.99					10			2
55183	RIF1		295054210	0.98	3	5			10	13	6	4
9524	TECR		24475816	0.98	2	2	4		10	8	6	5
509	ATP5C1		4885079	0.98	7	4	2		10	18	13	10
284058	KANSL1		301500643	1					9	10	6	4
112939	NACC1		16418383	1					9	10	6	7
81930	KIF18A		148612831	1					9	8	5	7
58509	C19orf29		122937392	1					9	6	5	8
51639	SF3B14		7706326	1					9	13	9	10
23511	NUP188		62955803	1					9	6	4	2
10902	BRD8		256223315	1					9	9	10	11
10556	RPP30		157151755	1					9	6	7	7
9824	ARHGAP11A		7661858	1					9	5	6	7
9611	NCOR1		22538461	1					9	9	13	7
6880	TAF9		4507351	1					9	13	14	12
6878	TAF6		21536359	1					9	9	10	8
6154	RPL26		4506621	1	2	3			9	11	10	8
1459	CSNK2A2		4503097	1	3				9	7	5	3
1457	CSNK2A1		29570791	1		3			9	5	9	6
57492	ARID1B		297139703	0.99					9			4
5981	RFC1		32528306	0.99	4				9	13	7	9
53615	MBD3		4505119	0.98	5				9	10	9	7
151246	SGOL2		229892197	1					8		5	5
84916	CIRH1A		186928847	1					8	8	7	6
55320	MIS18BP1		42415492	1					8	11	8	7
54443	ANLN		31657094	1					8	7	8	4
51780	KDM3B		38372909	1		2			8	8	10	5
51616	TAF9B		20070280	1					8	9	12	
51562	MBIP		222080053	1					8	7	4	4
29997	GLTSCR2		239787829	1					8	5	3	5
27340	UTP20		120587023	1					8	7	3	5
26993	AKAP8L		49472841	1					8	8	3	3
26574	AATF		7657013	1					8	8	6	4
25902	MTHFD1L		36796743	1					8	3	3	

23126	POGZ		302699211	1					8	6	6	7
11091	WDR5		16554627	1					8	6	6	7
10933	MORF4L1		45643135	1					8	10	8	5
10569	SLU7		27477111	1					8	9	4	7
10528	NOP56		32483374	1	2	2			8	6	4	3
10452	TOMM40		193083120	1	2				8	12	5	3
9987	HNRPDL		14110407	1					8	8	3	7
9646	CTR9		7661950	1					8		6	2
9126	SMC3		4885399	1					8	6	3	5
6877	TAF5		21071067	1					8	4	3	4
6294	SAFB		21264343	1					8	6		
6232	RPS27		4506711	1		2			8	7	6	6
5700	PSMC1		24430151	1					8	6	3	5
5356	PLRG1		320118865	1	2				8	5	4	3
3725	JUN		4758616	1		2	3		8	10	6	8
2961	GTF2E2		4504195	1					8		8	5
1968	EIF2S3		4503507	1			2		8	6	5	2
1460	CSNK2B		23503295	1					8	7	7	2
1063	CENPF		55770834	1					8	5		3
80335	WDR82		147904340	0.99	3	3			8	6	7	8
11169	WDHD1		5901892	0.99					8	4	3	5
7203	CCT3		58761484	0.99	2	4			8	7	10	8
6135	RPL11		15431290	0.99	2	2	4		8	10	6	7
3182	HNRNPAB		55956919	0.99	3				8	10	8	6
54856	GON4L		82830424	0.98	4				8	11	8	8
7531	YWHAE		5803225	0.98		4			8	6	7	4
65083	NOL6		18644728	1					7	8	5	4
57649	PHF12		75677357	1					7	12	8	7
56993	TOMM22		9910382	1					7	7	4	6
55143	CDCA8		8922438	1					7	4	3	6
55127	HEATR1		73695475	1					7	4		3
11177	BAZ1A		32967603	1					7	3	2	
10939	AFG3L2		300192933	1					7	6		2
10919	EHMT2		156142197	1					7	4	2	
10898	CPSF4		125987603	1					7	7	5	5
10657	KHDRBS1		5730027	1					7	5	7	
9070	ASH2L		157412280	1					7	4	3	3
8602	NOP14		55769587	1					7	6	6	7
7150	TOP1		11225260	1					7	9	10	11
6224	RPS20		226246671	1	2	3	2		7	3	4	7
5985	RFC5		194306567	1					7	3	3	
5520	PPP2R2A		294832006	1					7	3	2	
4236	MFAP1		50726968	1					7	6	3	6
641	BLM		4557365	1					7	5	5	2
103	ADAR		301601658	1	2				7	8	9	7
9588	PRDX6		4758638	0.99					7	4	2	2
6134	RPL10		223890243	0.98		3			7	6	6	5
3028	HSD17B10		4758504	0.98		4			7	10	8	8
100529239	RPS10-NUDT3		321117084	1					6	4	4	4
222229	LRWD1		23097240	1					6	4	3	4
91748	C14orf43		112807226	1					6	5	5	4
81887	LAS1L		13654270	1					6	5	3	4
64794	DDX31		17505907	1					6	2	6	5
57794	SUGP1		33469964	1					6	5	5	2
57510	XPO5		22748937	1					6	7	4	4
55602	CDKN2AIP		8923040	1					6	7	2	5
55578	FAM48A		8923735	1					6	5	6	
54934	KANSL2		154426300	1					6	10	7	7
54623	PAF1		42476169	1					6	5	6	7
27043	PELP1		155030232	1					6	3	4	
26054	SENP6		156105701	1					6		4	2
10915	TCERG1		21327715	1					6	3		4
9493	KIF23		20143967	1					6	5	5	2
9352	TXNL1		4759274	1					6	4	4	4
9123	SLC16A3		109288010	1					6	4		
8886	DDX18		38327634	1					6	7	8	6
8813	DPM1		4503363	1					6	7	2	3
8451	CUL4A		11140811	1					6	6		4
7290	HIRA		21536485	1					6	6	6	4
7023	TFAP4		4507447	1					6	7		
6472	SHMT2		19923315	1					6	6	6	
6428	SRSF3		4506901	1					6	6	5	5
6230	RPS25		4506707	1					6	7	7	5
6155	RPL27		4506623	1					6	6	4	5
3836	KPNA1		222144293	1					6	6	4	9
3185	HNRNPF		148470397	1					6	8	8	6
84444	DOT1L		22094135	0.99					6			
23264	ZC3H7B		27881484	0.99					6	7	4	
22880	MORC2		7662340	0.99					6	2	2	
7879	RAB7A		34147513	0.99			2		6	8	5	5
6418	SET		170763498	0.98			2		6	3	2	4
643752	RAP1BL		310114787	0.97		2			6	6	3	4
25836	NIPBL		47578105	0.97	2				6	4	4	4
113130	CDCA5		18087845	1					5	5	4	4
84154	RPF2		39930469	1					5	5	3	
84108	PCGF6		58761530	1					5	5	5	6
79595	SAP130		19923597	1					5	2	3	
64397	ZFP106		11968023	1					5	7	6	
55922	NKRF		291084505	1					5	6	4	3
55035	NOL8		46048234	1					5	4		7
29127	RACGAP1		186910300	1					5	7	4	5
26155	NOC2L		157694511	1					5	8	9	7
25792	CIZ1		196115141	1					5		4	
23613	ZMYND8		34335262	1					5	10	8	5
23517	SKIV2L2		193211480	1					5	5	2	3

23212	RRS1		14719402	1					5	5	6	5
23028	KDM1A		58761544	1					5	5	3	
10847	SRCAP		146219843	1					5	7	4	3
10412	NSA2		7662677	1					5	4	3	3
10153	CEBPZ		42542392	1					5	8	5	2
9439	MED23		28558969	1					5	5		
9320	TRIP12		10863903	1					5	3	3	
8243	SMC1A		30581135	1					5	4		
8061	FOSL1		4885243	1					5	3		4
5929	RBBP5		300796323	1					5	4		
5870	RAB6A		19923231	1					5	5	5	3
4798	NFRKB		219802034	1					5	3		
4779	NFE2L1		4505379	1					5	6	3	2
4175	MCM6		7427519	1					5	3	3	4
1642	DDB1		148529014	1					5	3	3	
1029	CDKN2A		300863096	1					5	3		4
191	AHCY		9951915	1					5	2	3	2
58	ACTA1		4501881	1					5	3		3
100510073	LOC100510073		310127973	0.99		2			5	6		2
54888	NSUN2		301336155	0.99					5	2		
7227	TRPS1		90652851	0.99					5	2	2	2
4176	MCM7		33469968	0.99					5	6	5	2
23	ABCF1		10947135	0.99	2				5	5	3	
51747	LUC7L3		19923485	0.98	2	2			5	7		
153443	SRFBP1		103471995	1					4	4	2	
125950	RAVER1		123173757	1					4	3	4	3
114823	LENG8		24308382	1					4	2	3	2
113251	LARP4		283046701	1					4	3	4	3
84365	MKI67IP		222352111	1					4	2	5	2
84318	CCDC77		14150165	1					4	5	6	3
84146	ZNF644		41152093	1					4	5	6	5
79954	NOL10		171460958	1					4	4		4
79707	NOL9		40217805	1					4	8	5	6
79228	THOC6		215272341	1					4	3		
63967	CLSPN		21735569	1					4	4		
56915	EXOSC5		47174864	1					4		3	3
55621	TRMT1		209862871	1					4	3		2
55252	ASXL2		153792780	1					4	5	6	3
54906	FAM208B		296011010	1					4	6		
54799	MBTD1		158508476	1					4	4	4	5
54555	DDX49		31542656	1					4	5	2	3
51574	LARP7		109809739	1					4	3		2
51322	WAC		18379328	1					4	4		2
27341	RRP7A		25092725	1					4	3	5	3
26121	PRPF31		221136939	1					4	3		
10952	SEC61B		5803165	1					4	3		
10945	KDELR1		5803048	1					4	5		
10212	DDX39A		21040371	1					4	5	5	4
10112	KIF20A		5032013	1					4	4		
9810	RNF40		7662230	1					4	3		
9785	DHX38		17999539	1					4		3	2
9775	EIF4A3		7661920	1					4	6	4	4
9425	CDYL		221136753	1					4	5	3	2
7874	USP7		150378533	1					4	5	4	3
6874	TAF4		110832843	1					4	3	4	2
6239	RREB1		270132929	1					4		5	
6218	RPS17L		312284072	1					4	2	3	4
5393	EXOSC9		77812670	1					4	5	5	4
4704	NDUFA9		6681764	1					4	5		5
4298	MLLT1		21361272	1					4	3	2	3
4100	MAGEA1		148276977	1					4	2	4	3
3839	KPNA3		34485722	1					4	4		2
38	ACAT1		4557237	1					4	4	3	2
84467	FBN3		56237021	0.99					4			
65993	MRPS34		13027604	0.99					4			
64710	NUCKS1		56118310	0.99					4			
51637	C14orf166		7706322	0.99					4	2	2	
10940	POP1		225007648	0.99					4	2	2	2
10629	TAF6L		5453844	0.99					4	2	2	2
9585	KIF20B		46049114	0.99					4	2		
9410	SNRNP40		115298668	0.99					4	2		
1871	E2F3		4503433	0.99					4			
23160	WDR43		157743245	0.97	2				4		3	5
286826	LIN9		32996737	1					3	4	3	
116092	DNTTIP1		16418441	1					3	3		
112869	CCDC101		19923935	1					3			
92609	TIMM50		48526509	1					3	6	3	3
84678	KDM2B		54112380	1					3	4	5	2
84148	KAT8		226371636	1					3	4	5	5
83759	RBM4B		13899354	1					3	3		2
79039	DDX54		164419743	1					3	3	5	3
57619	SHROOM3		203098098	1					3		4	
57109	REXO4		76781492	1					3		5	4
56254	RNF20		34878777	1					3	3	3	3
55257	C20orf20		8922764	1					3	3	4	2
51535	PPHLN1		219842247	1					3	5	7	
51434	ANAPC7		212549736	1					3	2	2	4
29117	BRD7		291219913	1					3	4		
27107	ZBTB11		166235167	1					3	3	2	
23347	SMCHD1		148839305	1					3	6	4	3
23244	PDS5A		155030216	1					3	6		3
23016	EXOSC7		189083688	1					3	3	3	
10989	IMMT		154354962	1					3	3		2
10171	RCL1		157426877	1					3	3	3	4
9643	MORF4L2		215490021	1					3			4

9577	BRE		21361171	1					3		4	
9055	PRC1		40807443	1					3		3	
7171	TPM4		4507651	1					3	2	6	5
7020	TFAP2A		109389358	1					3			3
6732	SRPK1		47419936	1					3	3	2	2
6638	SNRPN		13027644	1					3		3	
6603	SMARCD2		148536864	1					3	4	4	3
6434	TRA2B		4759098	1					3	6	3	3
5878	RAB5C		41393545	1					3	3		
5707	PSMD1		25777600	1					3	3	3	
4931	NVL		45643123	1					3	3		
3070	HELLS		21914927	1					3	6		
2355	FOSL2		4885245	1					3	4	2	2
1998	ELF2		42544172	1					3		2	3
124245	ZC3H18		255652953	0.99					3	2	2	
117143	TADA1		16596696	0.99					3	2		
79002	C19orf43		13128992	0.99					3	7	2	
64426	SUDS3		75677351	0.99					3	2		
10981	RAB32		5803133	0.99					3		2	
10885	WDR3		5803221	0.99					3	2		
9904	RBM19		226497574	0.99					3	2	2	2
8535	CBX4		55770830	0.99					3	2		
8289	ARID1A		21264565	0.99					3		2	
6176	RPLP1		4506669	0.99					3	2	2	2
4904	YBX1		34098946	0.99					3	6	2	3
4698	NDUFA5		4826848	0.99					3	2	2	
4513	COX2		251831110	0.99					3			
3833	KIFC1		167555110	0.99			2		3	9	6	6
2778	GNAS		117938759	0.99					3	6		4
1650	DDOST		20070197	0.99					3	4	2	
1523	CUX1		148277064	0.99					3	7		
1431	CS		38327625	0.99					3		2	
9589	WTAP		21361159	0.98					3	2	3	2
25824	PRDX5		32455260	0.96					3	6	3	2
85395	FAM207A		17158023	1					2	3	4	
64425	POLR1E		11968047	1					2	4	3	
51497	TH1L		39812492	1					2	3	4	
51010	EXOSC3		50511939	1					2	3	3	
23530	NNT		122939153	1					2	4		3
23310	NCAPD3		45356151	1					2	2	4	3
23076	RRP1B		57863269	1					2	6	5	
10935	PRDX3		32483377	1					2	4	2	3
6838	SURF6		19557702	1					2	2	3	3
6223	RPS19		4506695	1					2		8	8
5705	PSMC5		24497435	1					2	4	6	
5501	PPP1CC		4506007	1					2	9	2	2
4605	MYBL2		4505293	1					2	5	4	4
2108	ETFA		189181759	1					2	3	3	
1022	CDK7		4502743	1					2		4	3
692312	PPAN-P2RY11		310923196	0.99					2		4	
653252	TIMM23B		310120500	0.99					2	2	2	
400506	C16orf88		111607441	0.99					2		2	2
140876	FAM65C		109689705	0.99					2			2
118460	EXOSC6		17402904	0.99					2		2	
88745	RRP36		24308350	0.99					2			2
84946	LTV1		21361875	0.99					2			2
84321	THOC3		14150171	0.99					2	2	3	2
83855	KLF16		13994287	0.99					2	4		2
81565	NDEL1		13540600	0.99					2			
64965	MRPS9		33188463	0.99					2	2	2	
64834	ELOVL1		13489093	0.99					2			
57332	CBX8		10190682	0.99					2	2	2	
57117	INTS12		21361851	0.99					2	3		
55726	ASUN		155030185	0.99					2		2	
55272	IMP3		8922794	0.99					2	2		
55215	FANCI		164607124	0.99					2			3
55157	DARS2		40789249	0.99					2			3
55131	RBM28		187960109	0.99					2	2		3
54617	INO80		38708321	0.99					2	2		4
54499	TMCO1		24308133	0.99					2			
51118	UTP11L		156415994	0.99					2			
51003	MED31		7705592	0.99					2	2	2	2
29789	OLA1		58761500	0.99					2		5	2
29079	MED4		7661788	0.99					2			2
26168	SENP3		21361499	0.99					2	3		
25873	RPL36		16117794	0.99					2	2	4	
23144	ZC3H3		155722994	0.99					2			
11338	U2AF2		6005926	0.99					2	2		
11047	ADRM1		28373192	0.99					2	2		4
11021	RAB35		5803135	0.99					2	2	2	
10979	FERMT2		201861813	0.99					2	2		
10772	SRSF10		16905517	0.99					2	3	2	2
10607	TBL3		19913369	0.99					2	2	2	2
10469	TIMM44		33636719	0.99					2	2	2	
10465	PPIH		5454154	0.99					2			
9968	MED12		110347429	0.99					2	4		
9933	KIAA0020		109948283	0.99					2	2	2	2
8872	CDC123		221316620	0.99					2			
8841	HDAC3		13128862	0.99					2	3	2	
8805	TRIM24		47419909	0.99					2	2	4	2
8667	EIF3H		4503515	0.99					2		2	
8565	YARS		4507947	0.99					2	2	3	
8086	AAAS		12962937	0.99					2	2		
7917	BAG6		149158692	0.99					2			2
7532	YWHAG		21464101	0.99					2	2		

7514	XPO1		4507943	0.99					2	2		2
7027	TFDP1		6005900	0.99					2		2	
6749	SSRP1		4507241	0.99					2	2	2	3
6602	SMARCD1		133908629	0.99					2		4	3
6389	SDHA		156416003	0.99					2	2	2	
6168	RPL37A		4506643	0.99					2	4		2
6156	RPL30		4506631	0.99					2		3	2
5702	PSMC3		21361144	0.99					2		3	2
5558	PRIM2		41349495	0.99					2	2	2	
5347	PLK1		21359873	0.99					2	2		
5073	PARN		197333695	0.99					2	3		
4774	NFIA		224465182	0.99					2	2	2	2
4728	NDUFS8		4505371	0.99					2			
4536	ND2		251831108	0.99					2			
4508	ATP6		251831112	0.99					2	2	2	
4292	MLH1		4557757	0.99					2			2
3251	HPRT1		4504483	0.99					2	2	2	
3015	H2AFZ		4504255	0.99					2		2	2
2963	GTF2F2		4758488	0.99					2	2		
2744	GLS		156104878	0.99					2		2	
1778	DYNC1H1		33350932	0.99					2			
1717	DHCR7		119943112	0.99					2	2	2	2
1629	DBT		110671329	0.99					2	2		2
1434	CSE1L		29029559	0.99					2			2
1191	CLU		283806712	0.99					2	2		
123624	AGBL1		120444924	1						3		3
90737	PAGE5		19743940	1								10
54904	WHSC1L1		13699811	1						4	4	
22913	RALY		21396480	1						4	3	
10623	POLR3C		21359969	1						6	5	
8930	MBD4		4505121	1						3	3	3
7133	TNFRSF1B		4507577	1						9	18	
6161	RPL32		4506635	1								2
5861	RAB1A		4758988	1						8		5
5162	PDHB		156564403	1								3
1468	SLC25A10		20149598	1						3	3	
253143	PRR14L		209969819	0.99							3	
221937	FOXK1		82546824	0.99						2	2	
196385	DNAH10		198442844	0.99						3		
93661	CAPZA3		15277417	0.99						2		
80145	THOC7		156151381	0.99						2	3	
80063	ATF7IP2		38569430	0.99							2	
64978	MRPL38		169636418	0.99								3
64783	RBM15		319996623	0.99						2		
64769	MEAF6		40255020	0.99						2		
60559	SPCS3		11345462	0.99						2		
55735	DNAJC11		217035105	0.99							2	2
55379	LRRC59		40254924	0.99							3	
54606	DDX56		9506931	0.99						3	2	
29102	DROSHA		155030234	0.99								2
25980	C20orf4		18034690	0.99						3		
25879	DCAF13		229892270	0.99							2	
23764	MAFF		239048350	0.99						2	2	
11214	AKAP13		21493029	0.99						2	3	
10916	MAGED2		19387846	0.99						2	3	
10744	PTTG2		154354976	0.99						2	2	
10427	SEC24B		112382212	0.99						2		3
10001	MED6		42544155	0.99						2	2	2
9512	PMPCB		94538354	0.99							2	
9025	RNF8		34304336	0.99						2		
7030	TFE3		8659574	0.99						3		
6942	TCF20		31652242	0.99						4		2
6921	TCEB1		5032161	0.99						3	2	
5717	PSMD11		28872725	0.99								2
5042	PABPC3		45238849	0.99						3		2
4719	NDUFS1		316983154	0.99						3		
4172	MCM3		6631095	0.99						4	2	
3609	ILF3		212549553	0.99						2	3	
3298	HSF2		207113147	0.99							2	
2915	GRM5		219842343	0.99								2
817	CAMK2D		212549753	0.99							2	5

Supplementary Table 1 b.

				2573 N-FlagBirA-HeLa_ONLY_Ctrl_B_v1(N-FlagBirA) 460	2574 N-FlagBirA-HeLa_ONLY_Ctrl_B_v2(N-FlagBirA) 460	2575 N-FlagBirA-HeLa_ONLY_Ctrl_C_v1(N-FlagBirA) 460	2576 N-FlagBirA-HeLa_ONLY_Ctrl_C_v2(N-FlagBirA) 460	2546 N-FlagBirA-HeLa_Myc_WT_B_v1(N-FlagBirA) 4609	2547 N-FlagBirA-HeLa_Myc_WT_B_v2(N-FlagBirA) 4609	2548 N-FlagBirA-HeLa_Myc_WT_C_v1(N-FlagBirA) 4609	2549 N-FlagBirA-HeLa_Myc_WT_C_v2(N-FlagBirA) 4609	MYC SAINT
Gene ID	Gene Name	LocusTag	Protein ID	Total Peptide	Total Peptide	Total Peptide	Total Peptide	Total Peptide	Total Peptide	Total Peptide	Total Peptide	Score
948469	birA	b3973	16131807	1023	644	742	451	251	216	175	153	
2316	FLNA	XX-FW83128A1.1	116063573	462	383	363	212	172	135	151	134	
79026	AHNAK		61743954	402	321	352	209	34	27	21	19	
5091	PC		106049292	300	233	203	130	870	788	707	640	0.01
2194	FASN		41872631	138	118	106	62	26	23	11	11	
5095	PCCA	RP11-151A6.1	65506442	131	94	63	43	289	286	257	233	
3304	HSPA1B	DAAP-21F2.7	167466173	130	101	108	76	275	232	232	200	
5096	PCCB		119943100	111	91	61	51	238	218	214	185	
5903	RANBP2		150418007	101	86	66	29	16	18	14	12	
3312	HSPA8		5729877	73	59	59	44	134	113	117	112	
7431	VIM	RP11-124N14.1	62414289	63	57	57	40	73	71	67	61	
4926	NUMA1		71361682	61	49	16	13	91	83	67	70	
22974	TPX2	RP11-243J16.10-002	20127519	60	40	53	28	98	81	74	83	
3305	HSPA1L	DADB-333F21.5	124256496	59	42	44	27	134	116	104	93	0.03
25909	AHCTF1	MSTP108	262359929	59	38	28	19	41	44	39	33	
7175	TPR		114155142	57	43	29	20	113	91	76	55	
1373	CPS1		169790915	56	37	38	24	118	104	96	76	
8467	SMARCA5		21071058	49	40	20	9	74	64	61	61	
4288	MKI67	RP11-380J17.2	103472005	46	29	28	18	167	129	110	114	1
3329	HSPD1		31542947	45	41	39	19	120	114	93	83	0.68
4869	NPM1		10835063	44	35	26	18	116	93	92	77	0.63
10694	CCT8		48762932	44	43	42	18	28	23	23	20	
9031	BAZ1B		14670392	41	39	26	10	75	58	50	48	
3326	HSP90AB1	RP1-302G2.1	20149594	39	35	36	21	57	43	45	45	
6949	TCOF1		207113160	39	30	31	22	32	29	26	26	
2017	CTTN		20357552	39	27	36	18	4				
4134	MAP4		197276600	39	27	33	18					
8363	HIST1H4J	RP1-160A22.4	11415030	37	24	11	9	30	32	29	20	
56922	MCCC1		116805327	36	30	29	12	146	131	116	112	1
1915	EEF1A1	RP11-505P4.2	4503471	34	23	23	15	47	42	38	37	
4841	NONO		224028244	33	24	32	15	84	78	69	62	0.78
1983	EIF5		21361337	33	19	22	10	2	3			
71	ACTG1		316659409	33	45	33	28	49		84	31	
1108	CHD4		51599156	31	20	17	5	133	100	92	80	1
22985	ACIN1		259906020	31	23	13	5	15	17	9	7	
11331	PHB2		221307584	30	25	16	9	44	42	39	31	
9774	BCLAF1		117938251	30	24	11	10	25	26	27	21	
5905	RANGAP1		4506411	30	22	23	10	3				
3313	HSPA9		24234688	29	20	18	9	100	90	87	78	1
57175	CORO1B		14149734	28	15	18	4	4	3		3	
4853	NOTCH2		24041035	28							18	
3320	HSP90AA1		153792590	27	28	23	13	45	36	30	31	
2317	FLNB		105990514	27	22	23	6					
1975	EIF4B		50053795	27	18	13	6	9	9	4	4	
9972	NUP153		24430146	27	19	23	16	25	18	13	16	
3190	HNRNPK	RP11-575L7.1	14165435	27	25	24	14	39	34	37	29	
64087	MCCC2		11545863	26	26	16	9	75	63	67	64	0.99
3310	HSPA6		34419635	25	18	22	17	41	30	44	31	
498	ATP5A1		4757810	25	20	19	9	66	61	58	56	1
84790	TUBA1C		14389309	24	29	26	16		25	25	57	
8021	NUP214	RP11-544A12.7	33946327	24	19	14	4				2	
4627	MYH9	RP1-68O2.1	12667788	24	13	7		13	13	5	6	
4361	MRE11A		5031923	24	17	22	8	39	29	27	24	
11083	DIDO1	RP5-885L7.1	301129165	24	21	12	6	43	42	43	25	0.01
3012	HIST1H2AE		10645195	23	9	9	9					
3054	HCFC1		98986457	23	15	13	5	133	108	98	105	1
3192	HNRNPU	RP11-11N7.3	14141161	23	19	15	6	30	24	26	26	
23451	SF3B1		54112117	22	16	11	6	69	56	48	41	1
3315	HSPB1		4504517	22	17	14	6	39	38	36	32	0.02
1938	EEF2		4503483	22	20	19	12	32	33	27	28	
5411	PNN		33356174	21	15	11	2	59	58	47	43	1
55226	NAT10		221316723	21	13	14	7	37	34	29	26	0.05
23607	CD2AP		11321634	21	15	10	6					
1973	EIF4A1		4503529	21	16	8	5	33	37	33	34	0.13
5052	PRDX1		320461711	20	19	11	6	26	20	21	16	
3309	HSPA5		16507237	20	19	19	7	27	27	14	17	
23524	SRRM2	HSPC075	118572613	20	14	4		26	23	21	23	0.33
23367	LARP1		39725634	20	15	12	8					
10155	TRIM28		5032179	19	14	17	6	35	34	32	27	0.02
203068	TUBB	DAAP-285E11.4	29788785	19	17	11	16	23	30	21	19	
9188	DDX21		50659095	19	11	5	7	60	50	46	40	1
302	ANXA2		209862831	19	21	23	12	34	30	20	19	
5514	PPP1R10	DADB-129D20.4	25777671	19	7	12	6	57	40	34	37	0.99
1665	DHX15		68509926	17	11	8		41	38	33	34	0.99
9967	THRAP3		167234419	17	11	4		23	16	13	10	0.13
22998	LIMCH1		163310741	17	14	16	9					
8407	TAGLN2	RP11-48O20.1	4507357	17	21	24	7	34	24	24	27	

7284	TUFM		34147630	16	15	7	3	53	49	35	34	1
26958	COPG2		109134349	16	8	21	10					
10992	SF3B2		55749531	16	8	12	8	66	57	52	39	1
26057	ANKRD17		38683807	16	10	8						
1981	EIF4G1		302699237	16	14	13	3	4	5	2	2	
7417	VDAC2	RP11-375G3.1	296317337	16	8	6	3	26	23	17	20	0.54
10009	ZBTB33		296179376	16	11	17	10	64	51	51	49	1
4670	HNRNPM		14141152	15	13	6	2	49	36	34	31	1
3178	HNRNPA1		14043070	15	9	8	3	27	24	29	22	0.96
8607	RUVBL1		4506753	15	15	14	6	48	46	38	35	1
6188	RPS3	OK/SW-cl.26	15718687	15	8	11	9	28	25	24	21	0.82
23450	SF3B3		54112121	15	13	10	8	35	33	27	26	1
9793	CKAP5		57164942	15	9	29	6	6	5			
6128	RPL6		16753227	15	9	10	2	36	25	26	21	0.95
23350	U2SURP		122937227	15	12	6	5	24	24	20	20	0.09
6421	SFPQ		4826998	14	16	17	10	37	25	25	31	0.46
9531	BAG3		14043024	14	7	6	2	8	5	7	9	
3181	HNRNPA2B1		14043072	14	6	4	4	23	19	18	18	0.79
22827	PUF60		17298690	14	11	12	3	7	7	3	4	
26135	SERBP1	CGI-55	66346679	14	16	12	4	10	14	11	10	
1660	DHX9		100913206	14	7	7	5	29	32	24	24	1
9879	DDX46		41327773	14	8	10	10	13	14	13	12	
9878	TOX4		7662274	13	11	13	3	37	33	27	26	0.98
23394	ADNP		12229217	13	11	7		84	54	63	55	1
9782	MATR3		21626466	13	10	6	2	28	20	15	15	0.57
292	SLC25A5		156071459	13	10	11	5	41	36	29	32	1
4000	LMNA	RP11-54H19.1	27436946	13	9			12	20	18	14	0.88
3183	HNRNPC		117189975	13	11	2		12	14	10	11	0.04
6520	SLC3A2		61744477	13	8	11	9	7	12	4	5	
5718	PSMD12		4506221	13	4	5	3	4	4	5	4	
10197	PSME3		30410794	13	6	10	2	17	13	15	14	0.01
6203	RPS9	XXbac-BCX105G6.6	14141193	13	9	5		18	20	17	12	0.82
871	SERPINH1	PIG14	32454741	13	11	13	6	44	29	29	20	1
506	ATP5B		32189394	13	10	8	4	35	32	28	29	1
10576	CCT2		311771535	13	8	7	6	18	17	12	11	0.01
5901	RAN	OK/SW-cl.81	5453555	12	10	10	6	26	25	28	19	0.98
9343	EFTUD2		217272892	12	8	4	3	35	23	15	13	0.99
9092	SART1		10863889	12	14	11	4	58	52	41	40	1
25929	GEMIN5		157739942	12	7	4						
221037	JMJD1C		118600981	12	9	7	3	82	63	72	59	1
23020	SNRNP200		40217847	12				45	44	27	31	1
10808	HSPH1	RP11-173P16.1	42544159	12	13	16	5	32	31	29	30	0.48
1654	DDX3X		301171467	12	8	11	4	17	15	13	5	
5701	PSMC2		4506209	12	7	7	3	5	3		5	
6137	RPL13	OK/SW-cl.46	15431295	12	11	6	6	17	19	16	19	0.14
55660	PRPF40A	HSPC225	151301228	11	5	3		11	11	8	10	0.47
7112	TMPO		73760405	11	13	10	18	14	12	9	6	
6194	RPS6	RP11-513M16.6	17158044	11	8	9	7	20	20	21	18	0.68
3066	HDAC2		293336691	11	14	4	2	33	25	28	28	1
11052	CPSF6		162329583	11	9	3	4	8	9	5	4	
1982	EIF4G2	OK/SW-cl.75	289577080	11	3	7			3	2		
58513	EPS15L1		10864047	11	7	7	2					
10291	SF3A1		5032087	11	7	3		11	11	8	10	0.03
64062	RBM26	PRO1777	31652264	11	4	5	4	13	9	12	16	0.25
9184	BUB3		4757880	11	5	5	3	5	8	5	5	
23476	BRD4		19718731	11	8	11	6	25	21	18	24	0.93
11335	CBX3		15082258	10	9	5	3	17	16	15	11	0.27
9733	SART3		7661952	10	4	4		13	11	11	8	0.47
7453	WARS		47419914	10	6	10	3					
6124	RPL4		16579885	10	10	7		31	23	24	24	1
800	CALD1		15149463	10	7	10	6					
56829	ZC3HAV1	PRO1677	27477136	10	6	4	6					
3726	JUNB		4504809	10	6	7	6	16	17	19	12	0.47
29889	GNL2		7019419	10	7	6		37	24	28	26	1
57532	NUFIP2	PIG1	32698730	10	3	9	6					
3006	HIST1H1C		4885375	10	7	11	8	12	12	16	15	
57092	PCNP		9966827	9	4	7	3	10	10	5	9	
3337	DNAJB1		5453690	9	7	12	2	22	20	22	24	0.78
10399	GNB2L1	HLC7	5174447	9	7	5	3	19	19	20	14	1
6627	SNRPA1		50593002	9		5	3			3		
10726	NUDC		5729953	9	7	4		2				
6122	RPL3	OK/SW-cl.32	4506649	9	8	7	3	25	18	25	16	1
9898	UBAP2L	RP11-205M9.4	188497756	9	2	6	2			2		
3837	KPNB1		19923142	9	4	3		8		6	5	0.09
6129	RPL7		15431301	9	4	3	2	18	16	10	16	1
6187	RPS2		15055539	9	7	4	3	13	10	9	9	0.43
81608	FIP1L1		201023339	9	6	5	2	20	23	16	16	1
10606	PAICS		119220557	9	8	10	3	4	5	3	3	
1655	DDX5		4758138	9	9	9	7	33	28	27	22	1
10146	G3BP1		38327552	9	3	3		2	3			
4678	NASP	RP4-697E16.3	27262628	9	9	6	2	4	4		2	
731751	LOC731751		113430845	9				39	26	25		1
3191	HNRNPL	P/OKcl.14	52632383	9	5	5	6	20	11	12	11	0.5
29894	CPSF1		56676371	9	6	2		16	12	13	11	0.81
51474	LIMA1	PP624	165905589	8	7	5						
7341	SUMO1	OK/SW-cl.43	4507801	8	3	3		2				
6629	SNRPB2		38149981	8	6	4	2	4	4	4	4	
10131	TRAP1		155722983	8	7	2	4	15	13	11	12	0.64
5478	PPIA		10863927	8	4	4		17	17	18	16	0.99
988	CDC5L	RP1-319D22.1	11067747	8	4	4		35	25	21	28	1
9221	NOLC1		148596949	8				4	3	6		0.09
27125	AFF4	HSPC092	7656879	8		4		13	14	13	12	0.93
9685	CLINT1		307078123	8	9	7	3	3	3	3	2	
10594	PRPF8		91208426	8	8			70	56	54	49	1
54439	RBM27		168229174	8		9		40	33	29	27	1
11051	NUDT21		5901926	8	3	5	2	14	11	9	9	0.67
10971	YWHAQ		5803227	8								
5250	SLC25A3	OK/SW-cl.48	4505775	8	7	2		20	10	13	14	0.9
157313	CDCA2		44681484	8	6	5		17	19	15	16	0.96
11273	ATXN2L		27262645	8	9	8	4					
3930	LBR	PRO0650	37595750	8	5	6	3	9	7	6	3	

708	C1QBP		4502491	8	6	4		20	16	11	14	0.98
4733	DRG1		4758796	8	4	4		2	2	5	4	
2597	GAPDH	CDABP0047	7669492	8	5		3	14	12	7	9	0.8
9787	DLGAP5		226371667	8	10	7	4	12	10	17	14	0.09
5339	PLEC		41322908	8	4	3		10	8	10	7	0.5
80218	NAA50		13376735	8	3	5	4					
6599	SMARCC1		188536047	7	3			42	24	31	31	1
7153	TOP2A		19913406	7				5	5	10	5	0.71
2037	EPB41L2	RP3-324N14.1	313661408	7	5	8	5					
10801	09-Sep		116256489	7	5	3	5					
5928	RBBP4		207029415	7		2	2	7	4	2		0.36
7001	PRDX2		32189392	7		5	2	11	12	10	9	0.81
4691	NCL		55956788	7	6	7		29	32	26	31	1
23404	EXOSC2	RP11-57C19.4	19923403	7	6	4		3	2		2	
8402	SLC25A11		21361114	7	4	6		20	19	13	14	1
8878	SQSTM1		214830438	7	5	3		14	11	10	7	0.85
6202	RPS8	RP11-269F19.3	4506743	7	4	6	4	18	15	19	21	1
6228	RPS23		4506701	7	4	4	2	12	13	12	14	0.99
2969	GTF2I		14670350	7	11	7	3	38	36	26	33	1
26064	RAI14		224809468	7	5	5						
3838	KPNA2		4504897	7	2			19	9	7	6	0.96
126353	C19orf21		27735067	7	5	4		2		2		
509	ATP5C1	RP11-401E9.1	4885079	7	4	2		10	18	13	10	0.98
27339	PRPF19		7657381	7	9	10	2	38	29	32	24	1
4676	NAP1L4		5174613	7		5						
11176	BAZ2A		91176325	7	8	3		23	19	18	13	0.99
22938	SNW1		6912676	7	7	5	5	25	27	21	20	1
8968	HIST1H3F		10440560	7	4			11	10	10	11	0.91
57187	THOC2	RP5-1189B24.5	125656165	7	3			15	12	12	8	0.97
57805	KIAA1967		24432106	7				44	32	28	23	1
3703	STT3A		22749415	6	2			6	5		2	0.17
9584	RBM39	RP11-353C18.2	35493811	6	4			19	16	17	17	1
5321	PLA2G4A		23943920	6	8	4	2					
3608	ILF2	RP11-354A16.1	24234747	6	4	5		15	12	12	9	0.99
7812	CSDE1		194473720	6	7	9	3					
6277	S100A6		7657532	6	2		4	4	4	5	3	0.01
9590	AKAP12		21493022	6	3	7						
6125	RPL5	MSTP030	14591909	6	6	2	2	16	16	17	15	1
6217	RPS16		4506691	6	3			9	10	10	8	0.99
6189	RPS3A		4506723	6	3	3		11	9	13	13	1
59	ACTA2	GIG46	213688375	6								
55609	ZNF280C	RP3-438D16.3	24308171	6				8	8	4	2	0.94
57142	RTN4	My043	24431933	6	5	6	3					
4637	MYL6		17986258	6	4	4		2	2	5	6	
10762	NUP50	CTA-268H5.7	24497447	6	7	2		5	5		5	
9532	BAG2	RP3-496N17.2	4757834	6	4	4		12	11	12	9	0.98
9129	PRPF3		4758556	6	7	4		12	10	10	8	0.5
2023	ENO1		4503571	6	5	4		17	18	17	11	1
9219	MTA2		14141170	6	7	7	4	32	29	25	27	1
5195	PEX14		4758896	6	6	2						
3615	IMPDH2	hCG_2002013	66933016	6		2		9	7	7	4	0.84
6208	RPS14	PRO2640	5032051	6	3	4		12	12	12	10	0.99
81	ACTN4		12025678	6	2	3					2	
6157	RPL27A		4506625	6	4	4	2	3	2	2	3	
23521	RPL13A		6912634	6	4	5		18	14	14	12	1
26986	PABPC1		46367787	6	6			5				0.04
57122	NUP107		9966881	6	4			5	5	3	2	0.23
10236	HNRNPR		156151394	6				16	10	6	7	0.99
3069	HDLBP		42716280	6	6	5	4					
23215	PRRC2C		115298682	6	4	5						
10226	PLIN3		255958282	6		2	4					
55752	11-Sep		8922712	5								
1937	EEF1G	PRO1608	4503481	5	2	7		2				
58517	RBM25		55741709	5	6	3		9	4	3	7	0.34
8473	OGT		32307148	5				39	26	20	22	1
5430	POLR2A		4505939	5								
10914	PAPOLA		32490557	5				3	7	3		0.67
53615	MBD3		4505119	5				9	10	9	7	0.98
56252	YLPM1		146134388	5					2			0.18
54908	CCDC99		157388975	5	4	6	3	30	18	22	20	1
6158	RPL28		13904866	5	4	2		10	10	9	9	0.96
908	CCT6A		4502643	5	2			4	2	4	2	0.04
8480	RAE1	RP4-800J21.2	4506399	5	4	6	3	8	4	4	2	0.01
1025	CDK9	RP11-228B15.5	4502747	5	4			10	7	3	8	0.97
58490	RPRD1B	RP5-1057B20.2	11034845	5				3				0.51
6159	RPL29		4506629	5	5	6	3	4	4	4	4	
3184	HNRNPD		14110414	5	2	4	2	10	9	7	6	0.9
4144	MAT2A		5174529	5	4	5	2					
9859	CEP170		109255228	5	3							
56949	XAB2	PP3898	55770906	5	3	4	5	33	32	19	15	1
26130	GAPVD1	RP11-184B22.1	51093832	5	3							
57496	MKL2		38569480	5	2							
5094	PCBP2		14141166	5	6	3	4	7	5	4	6	
63932	CXorf56		11545813	5	9	4	5	16	16	15	17	0.92
26156	RSL1D1	L12	118498359	5	2		3	4	5	6	5	0.32
27332	ZNF638		21626468	5	7	3		6	5	5	4	
4735	02-Sep		4758158	5	3	3	2					
7385	UQCRC2		50592988	5	4			21	11	17	15	1
10575	CCT4		38455427	5	4	2	2	5	7	2	3	0.18
283489	CHAMP1		255918139	5	7	7		16	8	8	5	0.48
6130	RPL7A	RP11-244N20.10-004	4506661	5	7	6	3	21	20	19	19	1
6597	SMARCA4		192807312	5	5	3		35	32	36	33	1
205860	TRIML2		27734883	5	3	6		14	16	13	11	0.99
6836	SURF4	RP11-244N20.4	19557691	5	3	5	2	11	7	7	4	0.47
51663	ZFR		34101286	5	4	4		12	10	11	11	0.96
1398	CRK		41327710	5				17	14	17	14	1
79882	ZC3H14		231570121	5	5	3		13	16	12	10	0.99
55161	TMEM33		224589127	5	5		4	9	7	8	7	0.37
6651	SON	HSPC310	21040326	5	5			7	9	3	2	0.83
4928	NUP98		21264365	5	8			7	10	3	4	0.47
4008	LMO7	RP11-332E3.2	221316634	5	4	6						

79180	EFHD2	RP3-467K16.3	20149675	5	7	4						
11171	STRAP		148727341	5	9	6	2					
81876	RAB1B		13569962	5	4	5	3	6		5		
7536	SF1		295842332	5		2		7	6	3	4	0.72
10611	PDLIM5	L9	197383077	5		2						
10383	TUBB4B	RP13-122B23.2	5174735	5	4		3	5	11	8	9	0.93
10521	DDX17	RP3-434P1.1	148613856	5		2		11	7	6	6	0.97
652797	LOC652797		310128730	5		6	2				8	0.19
9656	MDC1	DAAP-285E11.7	132626688	5				4	4	2		0.73
7798	LUZP1	RP1-184J9.3	216548085	5	3	2						
832	CAPZB	RP4-657E11.7	4826659	5	9	9	6					
6950	TCP1		57863257	4	2	11	3	26	28	29	28	1
55636	CHD7		54112403	4		2		23	20	20	18	1
8812	CCNK		150417989	4				5	7	3	4	0.87
1857	DVL3		41406097	4	2	2	3	5	2			0.04
6133	RPL9	OK/SW-cl.103	15431303	4	3	5	3	12	12	11	11	0.98
9612	NCOR2		116256445	4				2	2		2	0.55
10262	SF3B4	RP11-196G18.20-002	5032069	4	2			6	4	5	2	0.9
10574	CCT7		261399877	4				15	7	4	7	0.98
4666	NACA	HSD48	163965362	4	3	3	5	5	3			
375	ARF1		4502201	4	2	2		10	7	7	5	0.96
5832	ALDH18A1		21361368	4	2	5		23	23	17	13	1
6879	TAF7		14717407	4	2	2	3	16	11	10	6	1
23248	RPRD2	HSPC099	183396804	4	2	2		11	6	7	5	0.93
6222	RPS18	DADB-159G18.1	11968182	4				5	3	5	4	0.9
5981	RFC1		32528306	4				9	13	7	9	0.99
6634	SNRPD3		4759160	4		2		2				
84271	POLDIP3	RP1-222E13.9	29837655	4				4	6	4	3	0.85
546	ATRX	RP5-875J14.1	20336205	4				2				0.28
11333	PDAP1		7657441	4		4						
6165	RPL35A	GIG33	16117791	4	5	4	3	2	2			
6229	RPS24	RP11-6P10.1	14916501	4	2	2		10	10	10	10	1
100526740	ATP5J2-PTCD1		311893396	4	3	2		6	6	8	5	0.68
1829	DSG2		116534898	4		4	2					
445	ASS1	RP11-618A20.2	16950633	4	4	3		7	8	6	8	0.7
54856	GON4L	RP11-101O6.1	82830424	4				8	11	8	8	0.98
3619	INCENP		102467235	4				11	6	8	7	0.98
222194	RSBN1L		158534055	4	2							
6160	RPL31		148746199	4	2	4	2	9	8	8	8	0.98
7384	UQCRC1		46593007	4	3	2		9	8	2		0.95
10921	RNPS1		18379334	4	5			2	3	4	3	0.05
9045	RPL14		78000181	4	4	2		4	4	4	4	0.03
6210	RPS15A	OK/SW-cl.82	14165469	4	2	4		12	7	6	6	0.99
7094	TLN1	RP11-112J3.1	223029410	4								
1984	EIF5A		219555707	4		5	3	12	5	7	4	0.66
7314	UBB		11024714	4		3			8	6	7	0.83
2908	NR3C1		4504133	4	2			3	6	4	2	0.71
10856	RUVBL2	CGI-46	5730023	4	5	3		25	17	20	15	1
293	SLC25A6	RP11-261P4.6	156071462	4	4	3	3	11	7	6	4	0.55
6184	RPN1		4506675	4	3			15	15	14	8	1
6146	RPL22		4506613	4	3	4	3	4	4	4	4	
6152	RPL24		4506619	4	4	5	4	7	8	7	6	0.06
829	CAPZA1		5453597	4	2	6	2	4	2			
10128	LRPPRC		31621305	4				29	17	21	19	1
3187	HNRNPH1		5031753	4	9	5		9	9	8	8	0.03
4209	MEF2D	RP11-98G7.2	5174545	4	2			3	2	4	4	0.43
8140	SLC7A5		71979932	4	2	4	2					
1736	DKC1		215599015	4	3			8	10	5	5	0.97
9218	VAPA		94721250	4	3	2						
26227	PHGDH	RP4-683H9.1	23308577	4				5	5	4	3	0.83
8165	AKAP1		4502015	4	7							
4927	NUP88		24497453	4	7	4	2					
55833	UBAP2	RP11-176F3.6	22325364	4		2						
8498	RANBP3		194097487	4		3	3					
5469	MED1		28559039	4				16	6	3	3	0.97
10147	SUGP2		224282117	4				18	17	6	3	1
728689	EIF3CL		153791492	4	3	4		6	4	6	5	0.34
10890	RAB10		256222019	4		2		5	3	3	2	0.59
11190	CEP250	RP3-477O4.2	21735548	4			3			5		0.18
7082	TJP1		116875765	3	3	6						
6015	RING1	DADB-100D22.6	51479192	3				4		2	2	0.83
6191	RPS4X		4506725	3	4			23	14	20	15	1
6185	RPN2	RP3-343K2.2	209413738	3				14	12	10	11	1
1503	CTPS		148491070	3								
3040	HBA2		4504345	3								
10389	SCML2	RP5-1129A6.3	5174669	3	3			12	6	8	8	0.99
23468	CBX5		188035908	3	2			4	4	3		0.81
57539	WDR35		55743161	3								
989	07-Sep		148352329	3	5	4	3					
2775	GNAO1		162461738	3						2		0.35
11329	STK38		6005814	3								
5984	RFC4		31881687	3				5	3	2		0.93
142	PARP1	RP11-125A15.2	156523968	3	2	2		2	2			0.04
26750	RPS6KC1		19923723	3								
30836	DNTTIP2		54633315	3	2	2		23	24	22	20	1
9555	H2AFY		20336746	3	2			3	3	2	4	0.71
51552	RAB14	RP11-165P4.4	19923483	3		4	5	6	7	3	4	0.13
23481	PES1		7657455	3				13	11	9	8	1
440	ASNS		168229248	3								
54433	GAR1		15011916	3					2			0.22
10286	BCAS2		5031653	3	3			13	9	9	6	1
3182	HNRNPAB		55956919	3				8	10	8	6	0.99
5245	PHB		4505773	3	2	3		8	10	5	6	0.97
2237	FEN1		4758356	3	6			2		2	5	0.19
6234	RPS28		4506715	3		3						
79657	RPAP3		225735591	3		3		3	2			0.24
9169	SCAF11		117676384	3	5			12	13	9	11	0.98
55299	BRIX1		55770900	3				20	15	17	12	1
84656	GLYR1		40556376	3				5	2	2	3	0.71
51602	NOP58	HSPC120	7706254	3	3	3		23	19	22	13	1
22820	COPG1		11559929	3								

22824	HSPA4L		31541941	3						2		0.35
6142	RPL18A		11415026	3	2	3		11	10	12	9	1
25942	SIN3A		223941782	3				23	18	18	12	1
80335	WDR82	UNQ9342/PRO34047	147904340	3	3			8	6	7	8	0.99
3009	HIST1H1B		4885381	3	3		3	8	7	10	8	0.95
29927	SEC61A1		7019415	3				2	2			0.7
1459	CSNK2A2		4503097	3				9	7	5	3	1
55183	RIF1		295054210	3	5			10	13	6	4	0.98
4001	LMNB1		310689049	3	3			4	3			0.55
9053	MAP7		310750348	3								
26354	GNL3		45593130	3	2			21	17	15	19	1
9112	MTA1		115527080	3				12	12	10	7	1
11168	PSIP1		190014586	3	6	2		11	14	5	4	0.99
5688	PSMA7	RP5-1005F21.4	4506189	3				3	2	4	3	0.93
54815	GATAD2A		164519146	3		2	2	11	13	11	12	1
55749	CCAR1		46852388	3								
5862	RAB2A		4506365	3	6	7	5	7	5	4	4	
10432	RBM14		5454064	3	2			11	6	8	5	0.99
5093	PCBP1		222352151	3	2	4		5	6	4	5	0.41
6143	RPL19		4506609	3	3	4		6	5	6	5	0.72
6132	RPL8		15431306	3	3	2		6	5	6	5	0.92
23636	NUP62		24497603	3								
3301	DNAJA1	RP11-54K16.1	4504511	3	5	4	4	30	25	21	21	1
3017	HIST1H2BD		10800138	3	23	12	7		29	30	31	0.04
3043	HBB		4504349	3								
7419	VDAC3		208879465	3	4	4		10	12	8	8	0.96
83992	CTTNBP2		16975496	3								
5725	PTBP1		14165464	3	3	5	3	24	20	16	11	1
3065	HDAC1	RP4-811H24.2	13128860	3				32	23	23	15	1
10523	CHERP		119226260	3	2			18	10	9	8	1
4673	NAP1L1		21327708	3	3	4	3	4	2	2	2	
51755	CDK12		157817023	3					2	2		0.24
55746	NUP133	RP5-1068B5.4	26051235	3	2	4		3	3			0.03
10492	SYNCRIP	RP1-3J17.2	228008291	3	2	2				5	10	0.86
821	CANX		10716563	3	6	5	2	13	12	7	8	0.97
5709	PSMD3		25777612	3	3	4		13	6	5	5	0.85
493856	CISD2		56605994	3				2				0.35
1933	EEF1B2		11136628	3	3	4	3	3				
10857	PGRMC1		5729875	3				4	3	2		0.66
22948	CCT5		24307939	3				5	4	2	4	0.94
6897	TARS		38202255	3	2	3						
55100	WDR70		8922301	3	2	2		6	3	3	3	0.34
1303	COL12A1	RP1-238D15.1	93141047	3							2	0.35
3692	EIF6	RP4-614O4.1	31563374	2					2	2		0.39
6138	RPL15	TCBAP0781	15431293	2	3	4		22	15	16	17	1
140735	DYNLL2		18087855	2				3	2	2	2	0.55
10452	TOMM40		193083120	2				8	12	5	3	1
83743	GRWD1		237820620	2				4	4	3	3	0.88
6224	RPS20		226246671	2	3	2		7	3	4	7	1
134430	WDR36		21281677	2		3		20	19	19	17	1
25926	NOL11	L14	21361468	2				13	7	11	9	1
60312	AFAP1		197382472	2								
4321	MMP12		73858572	2								
23788	MTCH2	HSPC032	7657347	2					4	3	3	0.89
6144	RPL21	RP11-428O18.5	18104948	2	3	4	3	13	11	11	11	1
6510	SLC1A5		5032093	2	3	3		4	2			0.12
124944	C17orf49		218563737	2	2			2	4	2		0.64
3159	HMGA1	RP11-513I15.2	22208967	2				2		3	4	0.79
1994	ELAVL1		38201714	2								
1452	CSNK1A1		68303572	2				13	15	12	9	1
8218	CLTCL1		242246985	2				4				0.48
9128	PRPF4	RP11-110L1.4	24431950	2				12	7	6	6	1
103	ADAR	RP11-61L14.5	301601658	2				7	8	9	7	1
84433	CARD11		157743265	2								
205564	SENP5	FKSG45	159032029	2								
6154	RPL26		4506621	2	3			9	11	10	8	1
6633	SNRPD2		4759158	2				2	2	2	2	0.38
3376	IARS	RP11-62C3.1	94721239	2	2							
25978	CHMP2B	CGI-84	40254866	2								
10766	TOB2		7706739	2								
11079	RER1	RP4-740C4.2	116812591	2								
7277	TUBA4A		17921989	2				2	2	2		0.65
57414	RHBDD2		94818790	2								
515	ATP5F1	RP11-552M11.5	21361565	2				3	2		2	0.71
55011	PIH1D1		8923598	2								
4809	NHP2L1	CTA-216E10.8	4826860	2	2			2	2	2	2	0.39
51773	RSF1		38788333	2								
6730	SRP68		24497620	2		2						
23160	WDR43		157743245	2				4		3	5	0.97
10592	SMC2	PRO0324	110347418	2								
26092	TOR1AIP1	RP11-533E19.1	39753957	2								
11217	AKAP2	RP11-470J20.3	211971074	2								
904	CCNT1		17978466	2		2		17	14	10	11	1
6136	RPL12		4506597	2		4		12	9	8	7	1
7504	XK		10835267	2								
57186	RALGAPA2	RP11-470C13.2	118600961	2								
5687	PSMA6		23110944	2				2	2	3	3	0.94
206338	AQPEP		194239713	2								
3308	HSPA4		38327039	2								0.45
5356	PLRG1		320118865	2				8	5	4	3	1
10424	PGRMC2		291621647	2	2	2						
6141	RPL18		4506607	2	3	2		4	5	6	6	0.96
23	ABCF1	DADB-129D20.7	10947135	2				5	5	3		0.99
54407	SLC38A2		21361602	2			2	2	2			0.21
7353	UFD1L		29501813	2	2	2		2		2	2	0.04
100291837	LOC100291837		310113908	2		2						
51747	LUC7L3		19923485	2	2			5	7			0.98
359845	FAM101B		33186901	2	2	2						
1265	CNN2		41327730	2								
22992	KDM2A		16306580	2				5			2	0.89
23603	CORO1C		7656991	2		4						

9631	NUP155		24430149	2				4	2	2		0.88
26058	GIGYF2		156766043	2	3	2						
838	CASP5		209870069	2								
7919	DDX39B	DAAP-97M17.4	18375623	2								
494115	RBMXL1	RP4-531M19.2	21361809	2								
10946	SF3A3		5803167	2				3			3	0.91
5711	PSMD5	RP11-27I1.5	4826952	2	3	3						
6135	RPL11	RP11-223J15.3	15431290	2	2	4		8	10	6	7	0.99
23360	FNBP4		158534059	2								
9958	USP15		14149627	2				4		3	2	0.93
2035	EPB41	RP11-242O24.1	260436831	2								
2957	GTF2A1		7706735	2	2	2		2	3	2	2	0.2
8570	KHSRP		154355000	2	5	3	2	3	4			
7203	CCT3	RP11-443G18.6	58761484	2	4			8	7	10	8	0.99
488	ATP2A2		209413709	2		3		12	12	6	7	1
81555	YIPF5	PP12723	32401427	2								
7165	TPD52L2	RP4-591C20.2	40805860	2								
23344	ESYT1		14149680	2								
280717	ALB	BOS_6773	30794280	2								
9524	TECR		24475816	2	2	4		10	8	6	5	0.98
3550	IK		125988409	2	7	4	3	35	35	26	24	1
9295	SRSF11	RP4-677H15.3	300244569	2	2	2	2	7	3	4		0.66
3018	HIST1H2BB		10800140	2				55	32	29	30	1
1073	CFL2		14719392	2		2		3	3	3	3	0.16
83443	SF3B5	RP1-197L1.2	13775200	2		2		5			5	0.94
51495	PTPLAD1		117168248	2		2		5	3	5		0.95
5394	EXOSC10		4505917	2				38	27	21	20	1
10528	NOP56	RP4-686C3.1	32483374	2	2			8	6	4	3	1
25836	NIPBL		47578105	2				6	4	4	4	0.97
6231	RPS26	RP11-151A6.3	15011936	2	2	5		10	8	5	7	1
2107	ETF1		4759034	2					2			0.59
7295	TXN	RP11-427L11.1	50592994	2	2	2			4	2		0.23
10949	HNRNPA0		5803036	2	2	3		16	18	15	15	1
27430	MAT2B	MSTP045	11034825	2	2	2						
6426	SRSF1	OK/SW-cl.3	118582269	2				2				0.3
6175	RPLP0		16933546	2		2		4	5	5	4	0.99
57019	CIAPIN1	CUA001	89274169	2		3						
158358	KIAA2026		148612838		5							
5315	PKM2		33286418		5			17	10	9		0.99
652147	LOC652147		169218225		5	2						
7531	YWHAE		5803225		4			8	6	7	4	0.98
3028	HSD17B10	RP3-339A18.2	4758504		4			7	10	8	8	0.98
317772	HIST2H2AB		28195394		4							
5931	RBBP7	RP11-716A19.5	4506439		4			12	6	9	15	1
23469	PHF3	RP1-22I17.3	7662018		3	2		11	7	5	2	0.97
6867	TACC1		170763517		3	2	2					
22999	RIMS1	RP5-1046G13.1	41054864		3				2			0.35
6139	RPL17		313569768		3			8	5	5	6	0.97
5917	RARS		15149476		3	3						
9667	SAFB2		7661936		3					6		0.48
10963	STIP1		5803181		3							
6134	RPL10	XX-FW83563B9.1	223890243		3			7	6	6	5	0.98
6181	RPLP2		4506671		3							
1457	CSNK2A1		29570791		3			9	5	9	6	1
1936	EEF1D	UNQ601	194239727		3	4	3					
84661	DPY30		14211889		2							
51780	KDM3B		38372909		2			8	8	10	5	1
6232	RPS27	RP11-422P24.3	4506711		2			8	7	6	6	1
143244	EIF5AL1	RP11-342M3.3	153791632		2							
6169	RPL38		4506645		2	2		12	8	6	9	1
6605	SMARCE1		21264355		2	3		19	16	17	15	1
117154	DACH2	RP11-345E19.1	16876441		2							
127602	DNAH14	RP11-328N1.1	223555935		2		3					0.12
341350	OVCH1		110815798		2							
100510073	LOC100510073		310127973		2			5	6		2	0.99
51317	PHF21A	BM-006	156546894		2							
6432	SRSF7		306482694		2							
89122	TRIM4		15011941		2							0.85
84991	RBM17	RP11-414H17.9	14249678		2			11	10	7	5	1
1956	EGFR		29725609		2							
643752	RAP1BL	hCG_1757335	310114787		2			6	6	3	4	0.97
23309	SIN3B		52138513		2			30	26	24	22	1
58477	SRPRB	PSEC0230	284795266		2			2				0.31
27327	TNRC6A		116805348		2							
3725	JUN		4758616		2	3		8	10	6	8	1
6045	RNF2	GS1-120K12.1	6005747		2					6		0.5
4036	LRP2		126012573		2							0.4
117246	FTSJ3	SB92	194097365		2			30	27	28	27	1
31	ACACA		38679960			54	34	10	10	9	9	
32	ACACB		134142062			16		9	7	6	11	0.18
8241	RBM10	CTD-2522E6.5	20127479			7		12	6	6	8	0.94
10382	TUBB4A		21361322			5						
23345	SYNE1	RP1-130E4.2	154277116			5						
9748	SLK		41281453			4	2					
1832	DSP		58530840			4						
6642	SNX1		23111032			4						
701	BUB1B		59814247			4						
54464	XRN1		110624787			4						
261726	TIPRL	RP1-69E11.1	23097250			4						
2181	ACSL3		42794752			4		4	3			0.82
53353	LRP1B		93102379			3						
1287	COL4A5	RP6-24A23.5	15890086			3						
25957	PNISR	HSPC261	154146193			3		2				0.22
90861	HN1L	LA16c-431H6.1	21700763			3						
79649	MAP7D3	RP11-535K18.3	147903302			3						
4999	ORC2		5453830			3		14	10	7	4	1
7159	TP53BP2		112799849			3						
9669	EIF5B		84043963			3						
4659	PPP1R12A		219842212			3						
8533	COPS3		23238222			3						
7529	YWHAB	RP1-148E22.1	21328448			3					2	0.4

23211	ZC3H4		126723060			3						
51503	CWC15	AD-002	116812573			3		4	4	2		0.91
4076	CAPRIN1		42558250			2	2					
6173	RPL36A	RP1-164F3.1	316983124			2						
1968	EIF2S3		4503507			2		8	6	5	2	1
9097	USP14		4827050			2						
10598	AHSA1	HSPC322	6912280			2		4	7		2	0.94
9040	UBE2M		4507791			2	2					
8666	EIF3G		49472822			2						
3833	KIFC1	DAQB-126H3.5	167555110			2		3	9	6	6	0.99
7936	RDBP	DADB-122G4.4	14670268			2		3	4	3	2	0.93
6391	SDHC		4506863			2						
7879	RAB7A		34147513			2		6	8	5	5	0.99
2010	EMD	XX-FW88778H2.1	4557553			2	2	2	2			0.5
92140	MTDH		223555917			2						
6418	SET	RP11-216B9.3	170763498			2		6	3	2	4	0.98
10067	SCAMP3		16445419			2						
8761	PABPC4		208431833			2						
11325	DDX42		45446743			2		4			3	0.93
26586	CKAP2		148664201			2						
9212	AURKB		83776600			2		12	8	7	3	1
9261	MAPKAPK2		32481209			2						
100101267	POM121C		150378545			2		3	3			0.84
2318	FLNC		116805322				4					
57683	ZDBF2		151301098				3	2				0.35
441376	C8orf85		70778889				3			2	2	0.21
7534	YWHAZ		208973238				3	6	5	5	2	1
79869	CPSF7		209862881				3	5	6	4	3	0.94
7169	TPM2	RP11-112J3.4	47519616				3					
150379	PNPLA5	RP3-388M5.5	20304127				2					
7559	ZNF12		110349775				2					
4033	LRMP		42789729				2					
23523	CABIN1		313151181				2					
23283	CSTF2T		14149675				2	3	2			0.36
23011	RAB21		7661922				2					
120892	LRRK2		171846278				2					
7791	ZYX		4508047				2					
3799	KIF5B		4758648				2					
8295	TRRAP		4507691					305	255	224	223	1
57634	EP400		56549696					294	252	251	223	1
1912	PHC2		37595528					87	57	31	27	1
23269	MGA		256017163					65	48	55	42	1
57680	CHD8		282165704					62	44	54	50	1
1662	DDX10		13514831					52	48	49	36	1
55929	DMAP1	RP5-891H21.2	13123776					47	34	36	34	1
56946	C11orf30	GL002	19923559					46	37	38	34	1
80314	EPC1		13376810					44	36	41	36	1
2975	GTF3C1		101943240					42	37	25	31	1
4609	MYC		71774083					42	39	35	34	
23223	RRP12	RP11-452K12.13-007	223278379					34	23	28	20	1
10524	KAT5		36287049					34	28	36	28	1
57062	DDX24		9966805					32	30	23	21	1
86	ACTL6A		30089997					30	23	21	13	1
55388	MCM10	PRO2249	33383235					30	28	31	20	1
55689	YEATS2		33620755					29	28	25	21	1
23272	FAM208A		163838631					29	18	17	15	1
10813	UTP14A	RP4-537K23.16-001	21361348					28	26	29	19	1
6594	SMARCA1	RP3-353H6.2	164419749					28	20	19		1
56897	WRNIP1		18426902					26	28	30	28	1
9875	URB1		194394141					25	22	15	15	1
55791	LRIF1		55743124					24	28	25	19	1
26122	EPC2		194272186					22	19	18	19	1
23560	GTPBP4	RP11-38M7.5	55953087					22	12	19	20	1
2547	XRCC6	CTA-216E10.7	4503841					21	16	13	14	1
10199	MPHOSPH10		31317305					20	13	15	15	1
10376	TUBA1B		57013276					20				0.5
259266	ASPM	RP11-32D17.1	126116596					20	19	13	10	1
64324	NSD1		19923586					19	12	11	11	1
6601	SMARCC2		194363725					19	9	17	14	1
10445	MCRS1		29893564					18	12	18	13	1
654364	NME1-NME2	hCG_2001850	66392203					18	11	11	12	1
4149	MAX		21704263					18	14	13	16	1
23528	ZNF281		6912752					18	18	16	18	1
22803	XRN2	RP11-227D2.1	18860916					18	15	11	13	1
9330	GTF3C3		6912398					18	15	11	6	1
6944	VPS72	RP11-68I18.8	5174715					17	16	14	12	1
54880	BCOR		183396783					17	9	15	14	1
58525	WIZ		151301215					16	11	14	16	1
3096	HIVEP1	RP3-451B15.2	116805342					16	20	10	12	1
2091	FBL		12056465					16	14	15	14	1
79718	TBL1XR1		19913371					16	11	8	16	1
8089	YEATS4		5729838					16	16	15	15	1
79577	CDC73		40018640					16	15	15	14	1
83746	L3MBTL2		20149698					15	12	12	16	1
24144	TFIP11	HSPC006	56788356					15	11	6	4	1
64318	NOC3L		20806097					15	10	13	12	1
1107	CHD3		158420731					15	16	11	11	1
51366	UBR5		15147337					15	8	10	8	1
57167	SALL4		10047144					15	14	10	9	1
132660	LIN54		169234719					15	10	11	11	1
57459	GATAD2B		21218438					14	12	13	9	1
23279	NUP160		54859722					14	6	7	7	0.99
9877	ZC3H11A		114842410					14	10	13	8	1
51575	ESF1	HDCMC28P	18093112					14	12	9	10	1
55210	ATAD3A	RP5-832C2.1	283436222					14	12	16	16	1
220988	HNRNPA3		34740329					13	9	6	3	1
57050	UTP3		9966799					13	8	10	14	1
9349	RPL23		4506605					13	10	10	10	1
9328	GTF3C5	RP11-326L24.5	170763506					13	12	10	11	1
6209	RPS15		4506687					13	9	20	13	1
221504	ZBTB9	DASS-97D12.7	23308693					13	10	7	9	1

54556	ING3	HSPC301	38201655					13	14	12	11	1
24149	ZNF318	HRIHFB2436	120587019					13	9	8	7	1
23013	SPEN	RP1-134O19.1	14790190					13	8	8	5	1
254225	RNF169		148839382					13	10	2	5	1
24148	PRPF6		40807485					13	13	13		1
23186	RCOR1		7661892					13	11	12	8	1
7520	XRCC5		10863945					12		4	5	1
22984	PDCD11		70980549					12	3	5	5	1
10527	IPO7		5453998					12	4	4	4	0.98
10514	MYBBP1A		157694492					12	10	9	13	1
55683	KANSL3		169234787					12	11	6	8	1
10165	SLC25A13		237649019					12	12	7	6	1
6201	RPS7		4506741					12	10	12	12	1
54881	TEX10	RP11-208F1.2	239787838					12	12	7	10	1
51645	PPIL1	CGI-124	7706339					11	10	10	9	1
55661	DDX27	HSPC259	224593278					11	9	15	13	1
57798	GATAD1	tcag7.279	88759346					11	6	4	4	1
2186	BPTF		38788260					11	12	7	8	1
9790	BMS1	AL022344.1	224589071					11	11	12	6	1
4297	MLL	hCG_1732268	308199413					11	12	7	7	1
9329	GTF3C4		156119605					11	6	6	8	1
4839	NOP2		76150623					11	9	11	12	1
723790	HIST2H2AA4		106775678					11	13	11	18	1
643677	CCDC168	hCG_2011852	226246554					11				0.5
103910	MYL12B		15809016					11	8	7	7	1
6117	RPA1		4506583					11	9	10	7	1
7707	ZNF148		145386566					10	11	8	6	1
55646	LYAR	PNAS-5	224591430					10		4	5	1
56987	BBX		18378731					10	4	6	2	1
196528	ARID2		56549668					10	4	3	4	1
9757	MLL4		7662046					10			2	0.99
23246	BOP1		21327667					10	8	7	8	1
55193	PBRM1		30794372					10	9	3	4	1
23165	NUP205		57634534					10	7	5	4	1
7343	UBTF		115529449					10	9	8	8	1
10969	EBNA1BP2	RP5-1034F7.2	237649012					10	11	11	12	1
79915	ATAD5		26080431					10	4	5	2	1
29028	ATAD2	L16	24497618					10	9	9	7	1
5160	PDHA1	RP11-723P2.1	291084742					10	6	4	4	1
7690	ZNF131		84872173					10	4	8	3	1
10724	MGEA5		11024698					10	10	9	7	1
58509	C19orf29		122937392					9	6	5	8	1
9611	NCOR1		22538461					9	9	13	7	1
81930	KIF18A	OK/SW-cl.108	148612831					9	8	5	7	1
57492	ARID1B	RP11-419L10.1	297139703					9			4	0.99
9824	ARHGAP11A		7661858					9	5	6	7	1
23511	NUP188	RP11-167N5.2	62955803					9	6	4	2	1
51639	SF3B14	CGI-110	7706326					9	13	9	10	1
284058	KANSL1		301500643					9	10	6	4	1
10902	BRD8		256223315					9	9	10	11	1
112939	NACC1		16418383					9	10	6	7	1
6878	TAF6		21536359					9	9	10	8	1
10556	RPP30	RP11-320F15.1	157151755					9	6	7	7	1
6880	TAF9	AD-004	4507351					9	13	14	12	1
138474	TAF1L		24429572					9				0.5
51616	TAF9B	RP4-570L12.2	20070280					8	9	12		1
29997	GLTSCR2		239787829					8	5	3	5	1
6877	TAF5		21071067					8	4	3	4	1
9987	HNRPDL		14110407					8	8	3	7	1
26574	AATF	HSPC277	7657013					8	8	6	4	1
10569	SLU7		27477111					8	9	4	7	1
27340	UTP20		120587023					8	7	3	5	1
151246	SGOL2		229892197					8		5	5	1
1460	CSNK2B	DADB-127H9.2	23503295					8	7	7	2	1
55320	MIS18BP1		42415492					8	11	8	7	1
25902	MTHFD1L	RP1-292B18.2	36796743					8	3	3		1
9646	CTR9		7661950					8		6	2	1
5700	PSMC1		24430151					8	6	3	5	1
11091	WDR5		16554627					8	6	6	7	1
1063	CENPF	RP11-262H5.1	55770834					8	5		3	1
2961	GTF2E2		4504195					8		8	5	1
10933	MORF4L1	FWP006	45643135					8	10	8	5	1
54443	ANLN		31657094					8	7	8	4	1
51562	MBIP	BM-015	222080053					8	7	4	4	1
23126	POGZ	RP11-806J18.2	302699211					8	6	6	7	1
9126	SMC3		4885399					8	6	3	5	1
26993	AKAP8L	HRIHFB2018	49472841					8	8	3	3	1
11169	WDHD1		5901892					8	4	3	5	0.99
6294	SAFB		21264343					8	6			1
84916	CIRH1A		186928847					8	8	7	6	1
4236	MFAP1		50726968					7	6	3	6	1
23063	WAPAL	RP11-396M20.1	42734325					7				0.5
9070	ASH2L		157412280					7	4	3	3	1
7150	TOP1	RP3-511B24.1	11225260					7	9	10	11	1
5520	PPP2R2A		294832006					7	3	2		1
56993	TOMM22		9910382					7	7	4	6	1
57649	PHF12		75677357					7	12	8	7	1
9588	PRDX6		4758638					7	4	2	2	0.99
65083	NOL6		18644728					7	8	5	4	1
10919	EHMT2	DAAP-66K18.3	156142197					7	4	2		1
10657	KHDRBS1		5730027					7	5	7		1
11177	BAZ1A	HSPC317	32967603					7	3	2		1
7311	UBA52		4507761					7				0.5
8602	NOP14	RES4-25	55769587					7	6	6	7	1
10939	AFG3L2		300192933					7	6		2	1
55127	HEATR1	RP11-385F5.3	73695475					7	4		3	1
10898	CPSF4		125987603					7	7	5	5	1
55143	CDCA8		8922438					7	4	3	6	1
641	BLM		4557365					7	5	5	2	1
84726	PRRC2B	RP11-334J6.1	149192855					7				0.5
5985	RFC5		194306567					7	3	3		1

22880	MORC2		7662340					6	2	2		0.99
6472	SHMT2		19923315					6	6	6		1
222229	LRWD1		23097240					6	4	3	4	1
23264	ZC3H7B		27881484					6	7	4		0.99
6428	SRSF3		4506901					6	6	5	5	1
3836	KPNA1		222144293					6	6	4	9	1
100529239	RPS10-NUDT3		321117084					6	4	4	4	1
64794	DDX31		17505907					6	2	6	5	1
9493	KIF23		20143967					6	5	5	2	1
26973	CHORDC1		221316566					6				0.5
9352	TXNL1		4759274					6	4	4	4	1
84444	DOT1L		22094135					6				0.99
10042	HMGXB4	RP3-510H16.1	51173873					6	2			0.96
54623	PAF1		42476169					6	5	6	7	1
81887	LAS1L	RP3-475B7.2	13654270					6	5	3	4	1
7290	HIRA		21536485					6	6	6	4	1
57510	XPO5	RP3-337H4.5	22748937					6	7	4	4	1
8886	DDX18		38327634					6	7	8	6	1
8813	DPM1	RP5-914P20.2	4503363					6	7	2	3	1
9123	SLC16A3		109288010					6	4			1
57794	SUGP1		33469964					6	5	5	2	1
91748	C14orf43		112807226					6	5	5	4	1
6155	RPL27		4506623					6	6	4	5	1
6230	RPS25		4506707					6	7	7	5	1
7023	TFAP4		4507447					6	7			1
27043	PELP1		155030232					6	3	4		1
8451	CUL4A	RP11-391H12.1	11140811					6	6		4	1
55602	CDKN2AIP		8923040					6	7	2	5	1
55578	FAM48A	FP757	8923735					6	5	6		1
26054	SENP6	RP1-134M13.1	156105701					6		4	2	1
10915	TCERG1		21327715					6	3		4	1
54934	KANSL2		154426300					6	10	7	7	1
3185	HNRNPF		148470397					6	8	8	6	1
54888	NSUN2		301336155					5	2			0.99
84154	RPF2	RP11-397G5.1	39930469					5	5	3		1
55922	NKRF		291084505					5	6	4	3	1
8243	SMC1A	RP6-29D12.1	30581135					5	4			1
58	ACTA1	RP5-1068B5.2	4501881					5	3		3	1
7227	TRPS1		90652851					5	2	2	2	0.99
84108	PCGF6		58761530					5	5	5	6	1
26155	NOC2L		157694511					5	8	9	7	1
4176	MCM7		33469968					5	6	5	2	0.99
113130	CDCA5		18087845					5	5	4	4	1
11014	KDELR2		5803050					5		2		0.96
3189	HNRNPH3		14141157					5	2	2		0.98
79595	SAP130		19923597					5	2	3		1
64397	ZFP106		11968023					5	7	6		1
191	AHCY		9951915					5	2	3	2	1
25792	CIZ1	RP11-395P17.7	196115141					5		4		1
23517	SKIV2L2		193211480					5	5	2	3	1
9320	TRIP12		10863903					5	3	3		1
10847	SRCAP		146219843					5	7	4	3	1
4798	NFRKB		219802034					5	3			1
7975	MAFK		4505075					5				0.5
5870	RAB6A		19923231					5	5	5	3	1
10412	NSA2	HUSSY-29	7662677					5	4	3	3	1
9439	MED23	RP5-914N13.2	28558969					5	5			1
4175	MCM6		7427519					5	3	3	4	1
1029	CDKN2A		300863096					5	3		4	1
7416	VDAC1		4507879					5	3		2	0.91
388	RHOB		4757764					5				0.5
55035	NOL8	RP11-62C3.9	46048234					5	4		7	1
8061	FOSL1		4885243					5	3		4	1
10153	CEBPZ		42542392					5	8	5	2	1
23028	KDM1A	RP1-184J9.1	58761544					5	5	3		1
29127	RACGAP1		186910300					5	7	4	5	1
4779	NFE2L1		4505379					5	6	3	2	1
11056	DDX52	HUSSY-19	38569505					5				0.5
5929	RBBP5		300796323					5	4			1
23613	ZMYND8	RP5-890O15.1	34335262					5	10	8	5	1
1642	DDB1		148529014					5	3	3		1
23212	RRS1		14719402					5	5	6	5	1
10940	POP1		225007648					4	2	2	2	0.99
7874	USP7		150378533					4	5	4	3	1
38	ACAT1		4557237					4	4	3	2	1
84365	MKI67IP		222352111					4	2	5	2	1
84467	FBN3		56237021					4				0.99
54906	FAM208B		296011010					4	6			1
84318	CCDC77		14150165					4	5	6	3	1
4100	MAGEA1		148276977					4	2	4	3	1
3835	KIF22		6453818					4	4	2	3	0.91
10735	STAG2	RP11-517O1.1	112789526					4	3	3		0.89
79228	THOC6	PSEC0006	215272341					4	3			1
9410	SNRNP40		115298668					4	2			0.99
6790	AURKA	RP5-1167H4.6	38327562					4	2	4	2	0.96
467	ATF3	RP11-338C15.1	4502263					4				0.5
6874	TAF4	RP5-1107C24.1	110832843					4	3	4	2	1
10629	TAF6L		5453844					4	2	2	2	0.99
9425	CDYL		221136753					4	5	3	2	1
51637	C14orf166	CGI-99	7706322					4	2	2		0.99
79954	NOL10		171460958					4	4		4	1
125950	RAVER1	hCG_2033729	123173757					4	3	4	3	1
55621	TRMT1		209862871					4	3		2	1
10952	SEC61B		5803165					4	3			1
64710	NUCKS1	JC7	56118310					4				0.99
54799	MBTD1		158508476					4	4	4	5	1
7184	HSP90B1		4507677					4		2		0.9
79707	NOL9		40217805					4	8	5	6	1
54555	DDX49		31542656					4	5	2	3	1
100505503	RPS17L		312284072					4	2	3	4	1
9585	KIF20B		46049114					4	2			0.99

10112	KIF20A		5032013					4	4			1
9810	RNF40		7662230					4	3			1
6239	RREB1	RP11-69L16.1	270132929					4		5		1
5393	EXOSC9		77812670					4	5	5	4	1
9775	EIF4A3		7661920					4	6	4	4	1
84146	ZNF644		41152093					4	5	6	5	1
56915	EXOSC5		47174864					4		3	3	1
27341	RRP7A	CTA-126B4.5	25092725					4	3	5	3	1
153443	SRFBP1		103471995					4	4	2		1
1871	E2F3	RP1-177P22.2	4503433					4				0.99
55252	ASXL2		153792780					4	5	6	3	1
65993	MRPS34		13027604					4				0.99
51808	PHAX		66392146					4	2	2	3	0.95
4704	NDUFA9		6681764					4	5		5	1
57215	THAP11	HRIHFB2206	40354197					4				0.5
10945	KDELR1		5803048					4	5			1
3839	KPNA3	RP11-432M24.3	34485722					4	4		2	1
4298	MLLT1		21361272					4	3	2	3	1
63967	CLSPN		21735569					4	4			1
55147	RBM23	PP239	116734694					4				0.5
51322	WAC	RP11-48B24.1	18379328					4	4		2	1
9785	DHX38		17999539					4		3	2	1
51574	LARP7	HDCMA18P	109809739					4	3		2	1
4782	NFIC		45505151					4				0.5
26121	PRPF31		221136939					4	3			1
113251	LARP4	PP13296	283046701					4	3	4	3	1
5499	PPP1CA		4506003					4	2	3	7	0.96
221613	HIST1H2AA		25092737					4	3	4		1
8175	SF3A2		21361376					4	4	4	3	0.99
114823	LENG8	XXbac-BCX535A19.3	24308382					4	2	3	2	1
10212	DDX39A		21040371					4	5	5	4	1
4698	NDUFA5		4826848					3	2	2		0.99
4513	COX2		251831110					3				0.99
8289	ARID1A	RP1-50O24.1	21264565					3		2		0.99
10885	WDR3	RP4-776P7.1	5803221					3	2			0.99
7259	TSPYL1		62988322					3				0.5
6638	SNRPN		13027644					3		3		1
84148	KAT8	PP7073	226371636					3	4	5	5	1
1650	DDOST	OK/SW-cl.45	20070197					3	4	2		0.99
112869	CCDC101		19923935					3				1
3070	HELLS	Nbla10143	21914927					3	6			1
10989	IMMT	PIG4	154354962					3	3		2	1
3516	RBPJ		42560223					3				0.5
80153	EDC3	PP844	19923613					3				0.5
158763	ARHGAP36	RP13-102H20.1	40255080					3				0.5
57109	REXO4	RP11-244N20.5	76781492					3		5	4	1
57619	SHROOM3	MSTP013	203098098					3		4		1
64426	SUDS3		75677351					3	2			0.99
9361	LONP1		21396489					3				0.5
6164	RPL34		16117787					3	2	2	4	0.93
9904	RBM19		226497574					3	2	2	2	0.99
79039	DDX54		164419743					3	3	5	3	1
56254	RNF20		34878777					3	3	3	3	1
4904	YBX1		34098946					3	6	2	3	0.99
9589	WTAP	RP1-56L9.4	21361159					3	2	3	2	0.98
6176	RPLP1		4506669					3	2	2	2	0.99
55257	C20orf20		8922764					3	3	4	2	1
9577	BRE		21361171					3		4		1
1284	COL4A2	RP11-90L1.2	116256354					3				0.5
144108	SPTY2D1		51702222					3				0.5
51692	CPSF3		7706427					3				0.5
2778	GNAS	RP4-543J19.4	117938759					3	6		4	0.99
90390	MED30		18087811					3	2	2		0.95
8535	CBX4		55770830					3	2			0.99
1998	ELF2		42544172					3		2	3	1
55870	ASH1L		110349788					3				0.5
51535	PPHLN1	HSPC206	219842247					3	5	7		1
79002	C19orf43		13128992					3	7	2		0.99
1431	CS		38327625					3		2		0.99
7020	TFAP2A	RP1-290I10.1	109389358					3			3	1
64969	MRPS5		13994259					3				0.5
64682	ANAPC1		12056971					3				0.5
10981	RAB32		5803133					3		2		0.99
8621	CDK13		145309300					3				0.5
1104	RCC1	RP4-669K10.2	114796642					3	3			0.92
2355	FOSL2		4885245					3	4	2	2	1
6732	SRPK1	RP3-422H11.1	47419936					3	3	2	2	1
5977	DPF2		5454004					3				0.5
10171	RCL1	RP11-125K10.1	157426877					3	3	3	4	1
116092	DNTTIP1	RP3-447F3.5	16418441					3	3			1
6881	TAF10		5454106					3				0.5
5707	PSMD1		25777600					3	3	3		1
51434	ANAPC7		212549736					3	2	2	4	1
9643	MORF4L2	RP5-1055C14.2	215490021					3			4	1
83759	RBM4B		13899354					3	3		2	1
124245	ZC3H18		255652953					3	2	2		0.99
4931	NVL		45643123					3	3			1
5878	RAB5C		41393545					3	3			1
286826	LIN9		32996737					3	4	3		1
10419	PRMT5		20070220					3	2			0.98
84678	KDM2B		54112380					3	4	5	2	1
117143	TADA1		16596696					3	2			0.99
1523	CUX1		148277064					3	7			0.99
54512	EXOSC4		9506689					3		2	3	0.84
9055	PRC1		40807443					3		3		1
6513	SLC2A1		166795299					3				0.5
6603	SMARCD2	PRO2451	148536864					3	4	4	3	1
23244	PDS5A	PIG54	155030216					3	6		3	1
23347	SMCHD1		148839305					3	6	4	3	1
1877	E4F1		69885084					3				0.5
10423	CDIPT		5453906					3	2	2	2	0.95

6434	TRA2B		4759098					3	6	3	3	1
7171	TPM4		4507651					3	2	6	5	1
10075	HUWE1	RP3-339A18.4	61676188					3				0.5
23016	EXOSC7		189083688					3	3	3		1
29117	BRD7		291219913					3	4			1
27107	ZBTB11		166235167					3	3	2		1
25824	PRDX5	SBBI10	32455260					3	6	3	2	0.96
29128	UHRF1		115430233					3				0.93
92609	TIMM50	PRO1512	48526509					3	6	3	3	1
54982	CLN6		8923532					2				0.49
2108	ETFA		189181759					2	3	3		1
80205	CHD9	AD-013	95147342					2				0.46
5558	PRIM2	RP3-401D24.1	41349495					2	2	2		0.99
51096	UTP18	CDABP0061	118344456					2				0.49
4508	ATP6		251831112					2	2	2		0.99
27044	SND1		77404397					2				0.49
2146	EZH2		21361095					2				0.49
8872	CDC123	RP11-186N15.4	221316620					2				0.99
10469	TIMM44		33636719					2	2	2		0.99
64965	MRPS9		33188463					2	2	2		0.99
51270	TFDP3	RP3-358H7.2	189409125					2				0.49
10465	PPIH		5454154					2				0.99
4728	NDUFS8		4505371					2				0.99
4783	NFIL3		52630429					2				0.49
11021	RAB35		5803135					2	2	2		0.99
55339	WDR33		56243590					2				0.49
25873	RPL36		16117794					2	2	4		0.99
8086	AAAS	GL003	12962937					2	2			0.99
30968	STOML2	HSPC108	7305503					2				0.49
6723	SRM		63253298					2				0.49
10682	EBP		5729810					2				0.46
94104	GCFC1		22035565					2				0.49
10979	FERMT2		201861813					2	2			0.99
359948	IRF2BP2		116734704					2				0.49
10772	SRSF10	RP11-4M23.6	16905517					2	3	2	2	0.99
29105	C16orf80		8392875					2	2	2		0.77
26017	FAM32A	CGI-144	7661696					2	2	2	2	0.77
5351	PLOD1	RP5-1077B9.2	32307144					2				0.49
3939	LDHA	PIG19	207028494					2				0.49
4722	NDUFS3		4758788					2	2			0.92
84321	THOC3		14150171					2	2	3	2	0.99
1856	DVL2		4758216					2				0.49
54499	TMCO1	PNAS-10	24308133					2				0.99
140876	FAM65C		109689705					2			2	0.99
23326	USP22		150010639					2				0.49
55759	WDR12		217330644					2				0.49
54552	GNL3L	RP11-353K22.1	296317324					2				0.49
5422	POLA1		106507301					2				0.49
5691	PSMB3		22538465					2				0.49
84464	SLX4		63252863					2				0.49
6168	RPL37A		4506643					2	4		2	0.99
11047	ADRM1		28373192					2	2		4	0.99
51433	ANAPC5		20127553					2				0.49
3015	H2AFZ		4504255					2		2	2	0.99
8565	YARS		4507947					2	2	3		0.99
6838	SURF6		19557702					2	2	3	3	1
29796	UQCR10	HSPC119	41281885					2				0.49
84759	PCGF1		109240538					2				0.49
25920	COBRA1	RP13-122B23.3	20070260					2				0.49
85395	FAM207A	PRED56	17158023					2	3	4		1
171017	ZNF384		209180475					2				0.49
55131	RBM28		187960109					2	2		3	0.99
6602	SMARCD1		133908629					2		4	3	0.99
51491	NOP16	CGI-117	148747209					2				0.49
4536	ND2		251831108					2				0.99
64834	ELOVL1	CGI-88	13489093					2				0.99
138046	RALYL		154240692					2				0.49
51118	UTP11L	CGI-94	156415994					2				0.99
4292	MLH1		4557757					2			2	0.99
2305	FOXM1		42544161					2				0.49
653252	TIMM23B	RP11-592B15.7	310120500					2	2	2		0.99
8805	TRIM24		47419909					2	2	4	2	0.99
6223	RPS19		4506695					2		8	8	1
118856	MMP21		22218341					2				0.46
10250	SRRM1		42542379					2				0.46
55157	DARS2		40789249					2			3	0.99
55215	FANCI		164607124					2			3	0.99
6871	TADA2A	KL04P	260656022					2				0.49
55726	ASUN		155030185					2		2		0.99
3276	PRMT1		151301219					2				0.46
1629	DBT	RP11-305E17.3	110671329					2	2		2	0.99
26168	SENP3		21361499					2	3			0.99
1434	CSE1L		29029559					2			2	0.99
57602	USP36		122114651					2				0.49
692312	PPAN-P2RY11		310923196					2		4		0.99
88745	RRP36	HSPC253	24308350					2			2	0.99
6156	RPL30		4506631					2		3	2	0.99
7917	BAG6	DADB-70P7.10-021	149158692					2			2	0.99
54892	NCAPG2		116812586					2				0.49
22850	ADNP2		7662346					2				0.49
1778	DYNC1H1		33350932					2				0.99
5702	PSMC3		21361144					2		3	2	0.99
5983	RFC3		108773789					2				0.49
118460	EXOSC6		17402904					2		2		0.99
51340	CRNKL1	RP5-1002M8.1	124256489					2		2		0.92
1717	DHCR7		119943112					2	2	2	2	0.99
23144	ZC3H3		155722994					2				0.99
4605	MYBL2		4505293					2	5	4	4	1
29115	SAP30BP		9994179					2	2	2	2	0.04
23076	RRP1B		57863269					2	6	5		1
996	CDC27		167466175					2				0.49

2070	EYA4	RP11-704J17.4	26667257					2				0.49
5705	PSMC5		24497435					2	4	6		1
2744	GLS		156104878					2		2		0.99
57117	INTS12	SBBI22	21361851					2	3			0.99
11338	U2AF2		6005926					2	2			0.99
8841	HDAC3		13128862					2	3	2		0.99
54971	BANP		109698609					2				0.49
27316	RBMX	RP11-1114A5.1	56699409					2				0.49
1399	CRKL		4885153					2				0.49
2963	GTF2F2		4758488					2	2			0.99
9442	MED27		28558979					2				0.49
29079	MED4	RP11-90M2.2	7661788					2			2	0.99
5706	PSMC6		195539395					2	2			0.94
23310	NCAPD3		45356151					2	2	4	3	1
6837	MED22	RP11-244N20.9	19557695					2				0.49
6389	SDHA		156416003					2	2	2		0.99
5347	PLK1		21359873					2	2			0.99
5518	PPP2R1A		21361399					2				0.49
6749	SSRP1		4507241					2	2	2	3	0.99
23168	RTF1		195976782					2				0.49
6598	SMARCB1		27545326					2	2			0.82
6018	RLF	RP1-39G22.1	157671949					2				0.49
84549	MAK16		31543091					2				0.49
1022	CDK7		4502743					2		4	3	1
54617	INO80		38708321					2	2		4	0.99
6205	RPS11		4506681					2	2		2	0.8
10935	PRDX3		32483377					2	4	2	3	1
51497	TH1L	HSPC130	39812492					2	3	4		1
7027	TFDP1	RP11-230F18.1	6005900					2		2		0.99
51010	EXOSC3	CGI-102	50511939					2	3	3		1
224	ALDH3A2		4557303					2				0.49
301	ANXA1	RP11-71A24.1	4502101					2	2	2		0.8
23435	TARDBP	RP4-635E18.2	6678271					2	3	2	2	0.95
57082	CASC5		74048514					2				0.49
55720	TSR1		39780588					2		2		0.92
81565	NDEL1		13540600					2				0.99
9933	KIAA0020	RP11-526D20.2	109948283					2	2	2	2	0.99
5501	PPP1CC		4506007					2	9	2	2	1
8667	EIF3H		4503515					2		2		0.99
4774	NFIA	RP5-902P15.1	224465182					2	2	2	2	0.99
7415	VCP		6005942					2	2	2		0.95
5073	PARN		197333695					2	3			0.99
25855	BRMS1		17530785					2				0.49
10607	TBL3		19913369					2	2	2	2	0.99
83855	KLF16		13994287					2	4		2	0.99
64425	POLR1E		11968047					2	4	3		1
400506	C16orf88		111607441					2		2	2	0.99
9968	MED12		110347429					2	4			0.99
7532	YWHAG		21464101					2	2			0.99
51003	MED31	CGI-125	7705592					2	2	2	2	0.99
7514	XPO1		4507943					2	2		2	0.99
1191	CLU	AAG4	283806712					2	2			0.99
53981	CPSF2		34101288					2				0.46
57332	CBX8		10190682					2	2	2		0.99
23530	NNT	hCG_17428	122939153					2	4		3	1
6635	SNRPE		4507129					2	2	2	3	0.55
84946	LTV1		21361875					2			2	0.99
3251	HPRT1		4504483					2	2	2		0.99
55272	IMP3		8922794					2	2			0.99
29789	OLA1	PTD004	58761500					2		5	2	0.99
60	ACTB		4501885						39			0.5
7133	TNFRSF1B		4507577						9	18		1
5861	RAB1A		4758988						8		5	1
10623	POLR3C		21359969						6	5		1
6872	TAF1		20357585						6		2	0.96
10016	PDCD6		7019485						6			0.5
2079	ERH		4758302						4		4	0.95
2923	PDIA3		21361657						4			0.5
6942	TCF20	RP4-669P10.13-001	31652242						4		2	0.99
54904	WHSC1L1	DC28	13699811						4	4		1
22913	RALY	RP1-64K7.1	21396480						4	3		1
4172	MCM3	RP1-108C2.3	6631095						4	2		0.99
84833	USMG5	PD04912	14249376						4			0.5
347733	TUBB2B	RP11-506K6.1	29788768						4			0.5
55187	VPS13D		54607139						3			0.5
357	SHROOM2		4502175						3			0.5
123624	AGBL1		120444924						3		3	1
8930	MBD4		4505121						3	3	3	1
54606	DDX56		9506931						3	2		0.99
4719	NDUFS1		316983154						3			0.99
5139	PDE3A		70608155						3			0.5
56852	RAD18		256818821						3			0.5
23152	CIC		112421108						3			0.5
196385	DNAH10	hCG_1811879	198442844						3			0.99
7030	TFE3		8659574						3			0.99
51332	SPTBN5		170016061						3			0.42
43847	KLK14		91823048						3			0.5
10049	DNAJB6		17388799						3			0.5
6727	SRP14		149999611						3			0.5
1468	SLC25A10		20149598						3	3		1
6921	TCEB1		5032161						3	2		0.99
5042	PABPC3	RP11-165I9.5	45238849						3		2	0.99
84246	MED10	L6	49227854						3			0.5
5352	PLOD2		33636742						3			0.49
25980	C20orf4	CGI-23	18034690						3			0.99
80152	CENPT		126722969						3			0.5
675	BRCA2	RP11-298P3.4	119395734						2			0.49
221092	HNRNPUL2		118601081						2			0.49
80145	THOC7		156151381						2	3		0.99
9923	ZBTB40		139394556						2			0.49
10916	MAGED2	RP1-14O9.1	19387846						2	3		0.99

60559	SPCS3	UNQ1841/PRO3567	11345462						2			0.99
56164	STK31		14602443						2			0.49
6728	SRP19		4507213						2			0.49
4705	NDUFA10		4758768						2			0.49
121053	C12orf45		116256458						2			0.49
23764	MAFF	CTA-447C4.1	239048350						2	2		0.99
11214	AKAP13		21493029						2	3		0.99
291	SLC25A4		55749577						2			0.49
1195	CLK1		241666392						2			0.49
64783	RBM15		319996623						2			0.99
84263	HSDL2	RP11-32M23.1	308044580						2			0.49
345651	ACTBL2		63055057						2			0.4
10001	MED6		42544155						2	2	2	0.99
1017	CDK2		166362719						2			0.5
3609	ILF3		212549553						2	3		0.99
10290	SPEG		157785645						2			0.49
688	KLF5	RP11-505F3.5	14251215						2			0.49
10427	SEC24B		112382212						2		3	0.99
221937	FOXK1		82546824						2	2		0.99
400569	MED11	HSPC296	48717372						2			0.49
93661	CAPZA3		15277417						2			0.99
51406	NOL7	RP1-223E5.1	13569843						2			0.49
23312	DMXL2		119120894						2			0.49
6617	SNAPC1		4507101						2			0.49
136319	MTPN		21956645						2			0.49
23378	RRP8	hucep-1	12758125						2			0.49
7448	VTN		88853069						2			0.46
9025	RNF8		34304336						2			0.99
2193	FARSA		4758340						2			0.49
27154	BRPF3	RP3-524E15.1	148727368						2			0.49
81545	FBXO38	SP329	45505155						2			0.49
57018	CCNL1	BM-001	9945320						2			0.46
7090	TLE3		157384982						2			0.49
26009	ZZZ3		29789072						2			0.49
11244	ZHX1		63079680						2			0.46
64769	MEAF6	RP3-423B22.2	40255020						2			0.99
10744	PTTG2		154354976						2	2		0.99
22890	ZBTB1		182509178						2			0.49
51512	GTSE1	RP5-1163J1.2	253970412						2			0.49
100134189	LOC100134189		169178509						2			0.49
23682	RAB38		11641237						2			0.49
8668	EIF3I	RP4-675E8.1	4503513						2			0.49
10614	HEXIM1		5453682						2			0.46
8450	CUL4B		121114302							7		0.5
339488	TFAP2E		194353976							4		0.5
728642	CDK11A	RP1-283E3.2	148763345							4		0.5
4130	MAP1A		95147555							4		0.5
10897	YIF1A		170932464							3		0.5
55379	LRRC59	PRO1855	40254924							3		0.99
1454	CSNK1E	RP1-5O6.1	23199991							3		0.5
253143	PRR14L	RP4-694E4.2	209969819							3		0.99
7011	TEP1		21536371							3		0.5
6902	TBCA		4759212							3		0.5
56926	NCLN		51873031							3		0.5
4638	MYLK		116008188							3		0.5
55153	SDAD1		153791300							3		0.5
440829	SHISA8	C22:CTA-250D10.9	113429443							3		0.5
11283	CYP4F8		6005737							2		0.49
79747	ADGB	RP11-497D6.1	168823435							2		0.49
29072	SETD2	HSPC069	197313748							2		0.46
10775	POP4		5729986							2		0.49
2033	EP300	RP1-85F18.1	50345997							2		0.49
5686	PSMA5		23110942							2		0.49
55735	DNAJC11	RP1-126A5.3	217035105							2	2	0.99
23478	SEC11A		7657609							2		0.46
57631	LRCH2	RP6-204F4.4	154091020							2		0.49
79339	OR51B4		158081769							2		0.49
80063	ATF7IP2		38569430							2		0.99
65117	RSRC2		38146107							2		0.49
29911	HOOK2		154354985							2		0.49
5708	PSMD2		25777602							2		0.92
79084	WDR77	HKMT1069	13129110							2		0.46
6840	SVIL	RP11-534G20.1	150417971							2		0.49
9512	PMPCB		94538354							2		0.99
817	CAMK2D		212549753							2	5	0.99
5610	EIF2AK2		208431827							2		0.5
6147	RPL23A		17105394							2	2	0.95
23597	ACOT9	CGI-16	81295404							2		0.49
83849	SYT15	RP11-38L15.7	31881667							2		0.49
5110	PCMT1		226530908							2		0.49
404281	YY2		156119609							2		0.49
25879	DCAF13	HSPC064	229892270							2		0.99
55206	SBNO1		269846807							2		0.49
10923	SUB1		217330646							2	2	0.38
9689	BZW1		41281429							2		0.49
27288	RBMXL2		153252068							2		0.49
2182	ACSL4		12669909							2		0.4
4541	ND6		251831118							2		0.49
3298	HSF2		207113147							2		0.99
1021	CDK6		223718134							2		0.49
5591	PRKDC		126032350								23	0.5
90737	PAGE5	RP11-382F24.4	19743940								10	1
7273	TTN		291045225								8	0.5
653889	LOC653889		169172639								7	0.5
4542	MYO1F		27544941								4	0.5
10248	POP7	tcag7.985	153791431								4	0.5
64978	MRPL38	HSPC262	169636418								3	0.99
5162	PDHB		156564403								3	1
55138	FAM90A1		144953905								3	0.5
57673	BEND3		122937295								2	0.49
10474	TADA3		19743894								2	0.49

23424	TDRD7		112293287									2	0.49
11180	WDR6		197927448									2	0.49
9750	FAM65B		14277690									2	0.49
5717	PSMD11		28872725									2	0.99
10428	CFDP1		5453567									2	0.49
2915	GRM5		219842343									2	0.99
6161	RPL32	PP9932	4506635									2	1
2130	EWSR1	AC002059.7	253970500									2	0.49
3097	HIVEP2		110347463									2	0.46
84181	CHD6	RP4-620E11.2	29244924									2	0.46
29102	DROSHA		155030234									2	0.99
